# Modular Synthesis and Biological Investigation of 5-Hydroxymethyl Dibenzyl Butyrolactones and Related Lignans

**DOI:** 10.3390/molecules23123057

**Published:** 2018-11-22

**Authors:** Samuel J. Davidson, Lisa I. Pilkington, Nina C. Dempsey-Hibbert, Mohamed El-Mohtadi, Shiying Tang, Thomas Wainwright, Kathryn A. Whitehead, David Barker

**Affiliations:** 1 School of Chemical Sciences, University of Auckland, Aucklamd 1010, New Zealand; sdav134@aucklanduni.ac.nz (S.J.D.); lisa.pilkington@auckland.ac.nz (L.I.P.); 2 School of Healthcare Science, Manchester Metropolitan University, Manchester M1 5GD, UK; N.Dempsey-Hibbert@mmu.ac.uk (N.C.D.-H.); MOHAMED.EL-MOHTADI@stu.mmu.ac.uk (M.E.-M.); SHIYING.TANG@stu.mmu.ac.uk (S.T.); thomas.wainwright@stu.mmu.ac.uk (T.W.); K.A.Whitehead@mmu.ac.uk (K.A.W.); 3 The MacDiarmid Institute for Advanced Materials and Nanotechnology, Wellington 6140, New Zealand

**Keywords:** lignans, dibenzyl butyrolactones, anti-proliferative, acyl-Claisen, stereoselective synthesis

## Abstract

Dibenzyl butyrolactone lignans are well known for their excellent biological properties, particularly for their notable anti-proliferative activities. Herein we report a novel, efficient, convergent synthesis of dibenzyl butyrolactone lignans utilizing the acyl-Claisen rearrangement to stereoselectively prepare a key intermediate. The reported synthetic route enables the modification of these lignans to give rise to 5-hydroxymethyl derivatives of these lignans. The biological activities of these analogues were assessed, with derivatives showing an excellent cytotoxic profile which resulted in programmed cell death of Jurkat T-leukemia cells with less than 2% of the incubated cells entering a necrotic cell death pathway.

## 1. Introduction

**Figure 1:**
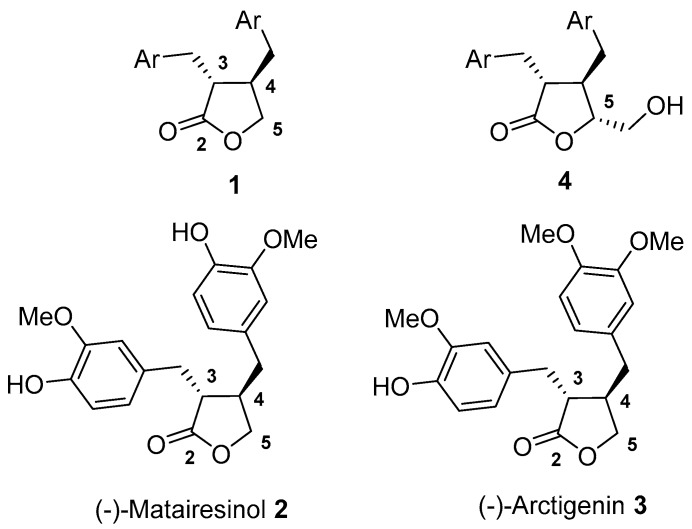
General structures of butyrolactone lignan **1**, natural dibenzylbutyrolactone lignans, (−)-matairesinol **2** and (−)-artigenin **3**, and 5-hydroxymethyl analogues **4**.

Dibenzyl butyrolactone lignans **1** are a class of lignans which have been reported to exhibit a range of biological activities, including, but not limited to neuroprotective [1], anti-cancer [2,3], anti-inflammatory [2,4], and anti-aging effects (see Figure 1) [5]. Perhaps the most notable of these biological properties is their reported potent anti-proliferative activities; examples of this class include (−)-matairesinol **2** and (−)-arctigenin **3** which, along with their synthesized derivatives, have been shown to exhibit excellent activity against various cancer cell lines, including pancreatic, breast, endometrial, colorectal, lung, and bladder cancers [6,7,8,9,10,11,12].

Owing to their anti-cancer properties and their classification as drug-like compounds [13] extensive work has gone into the study of these compounds and their related analogues to explore and establish structure–activity relationships and the possible use of these lignans as lead compounds for therapeutics. Whilst previous work has explored the synthesis of these lignans and analogues thereof [14,15,16], mainly focusing on changing the substituents on the aryl rings [17], one area that has not been extensively investigated is the synthesis of C-5 substituted analogues of these butyrolactone lignans, represented by **4**.

**Figure 2:**
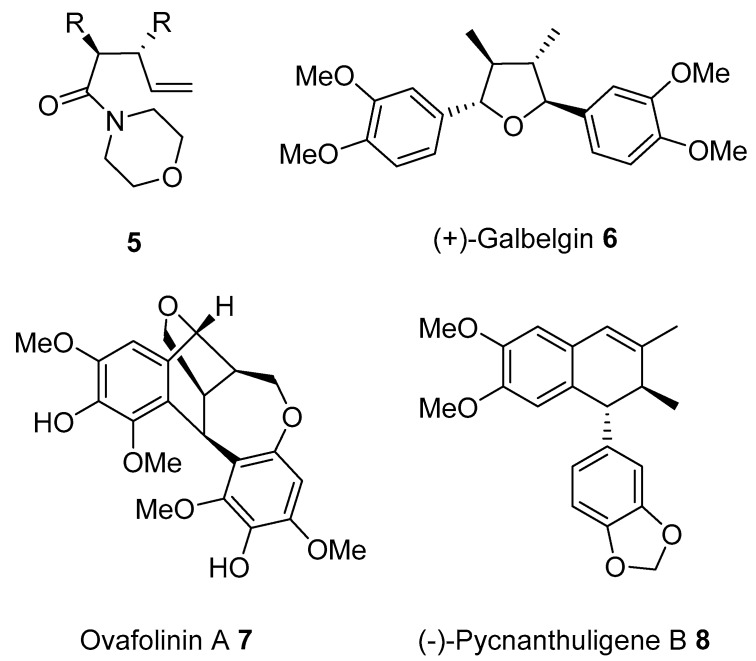
Use of amide **5**, the product of an acyl-Claisen rearrangement to access a number of lignan scaffolds and natural products **6**–**8**.

We have previously shown that the acyl-Claisen rearrangement can be used to prepare disubstituted morpholine pentenamides **5** with high diastereoselectivity at the C-3 and C-4 positions which correspond to the benzyl groups in the lactone scaffold (Figure 2) [18,19,20,21,22]. Furthermore, in our efforts to a prepare a number of different lignan scaffolds [18,19,20,21,22,23,24,25,26,27,28,29,30,31,32,33,34,35,36], we have used amides such as **5** to prepare compounds including tetrahydrofuran lignans (e.g., galbelgin **6**), aryltetralins (e.g., ovafolinin **7**) and aryl dihydronaphthalene lignans (e.g., (−)-pycananthuligene B **8**).

We wished to explore the usage of this methodology to synthesise butyrolactone lignans, as well as probe the effect of adding a substituent at the C-5 position on the biological activity. The route would be convergent and modular, allowing for simple modification of aromatic groups resulting in the synthesis of a number of analogues.

## 2. Results and Discussion

**Scheme 1:**
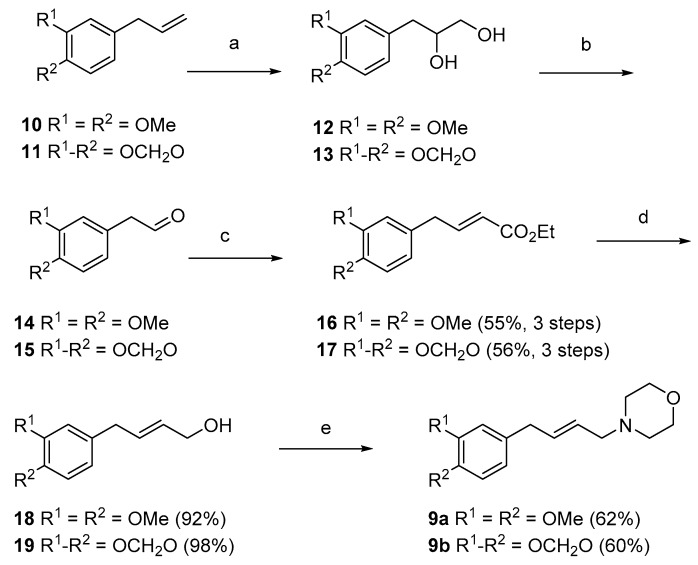
(**a**) OsO_4_ (0.1–0.3 mol%), *N*-methylmorpholine-*N*-oxide (3 eq.), *^t^*BuOH/H_2_O (1:1), 4 days; (**b**) NaIO_4_ (1.2 eq.), MeOH/H_2_O (3:1), 0.5–2 h; (**c**) Ph_3_PCHCO_2_Et (1.1 eq.), CH_2_Cl_2_, 16 h; (**d**) **18:** DIBAL-H (3 eq.), CH_2_Cl_2_, −78 °C, 10 min, **19:** DIBAL-H (2.2 eq.), toluene, −10 °C, 10 min; (**e**) Et_3_N (3 eq.), MsCl (1.2 eq.), morpholine (1.5 eq.), CH_2_Cl_2_, 0 °C, 2–18 h.

In order to utilise the acyl-Claisen rearrangement to prepare the desired lactones, the corresponding allylic morpholines and acid chlorides first needed to be synthesised. Allylic morpholines **9a** and **9b** were synthesised in five steps from 4-allyl-1,2-dimethoxybenzene **10** and safrole **11** (Scheme 1), respectively. Firstly, allylic benzenes **10** and **11** were dihydroxylated using catalytic osmium tetroxide giving **12** and **13**, followed by periodate cleavage to give aldehydes **14** and **15**. Aldehydes **14** and **15** were immediately used in a Wittig reaction with (carbethoxymethylene)-triphenylphosphorane to exclusively give the *E*-isomer of α,β-unsaturated esters **16** and **17**, in 55% and 56% yields, respectively, over three steps. The esters **16** and **17** were then reduced to allylic alcohols **18** and **19** using di-*iso*-butyl aluminium hydride (DIBAL-H) in excellent yields. Alcohols **18** and **19** were then converted to the corresponding allylic morpholines **9a** and **9b**, by first generating a mesylate in situ*,* which then underwent substitution to give allylic morpholines **9a** and **9b**.

**Scheme 2:**
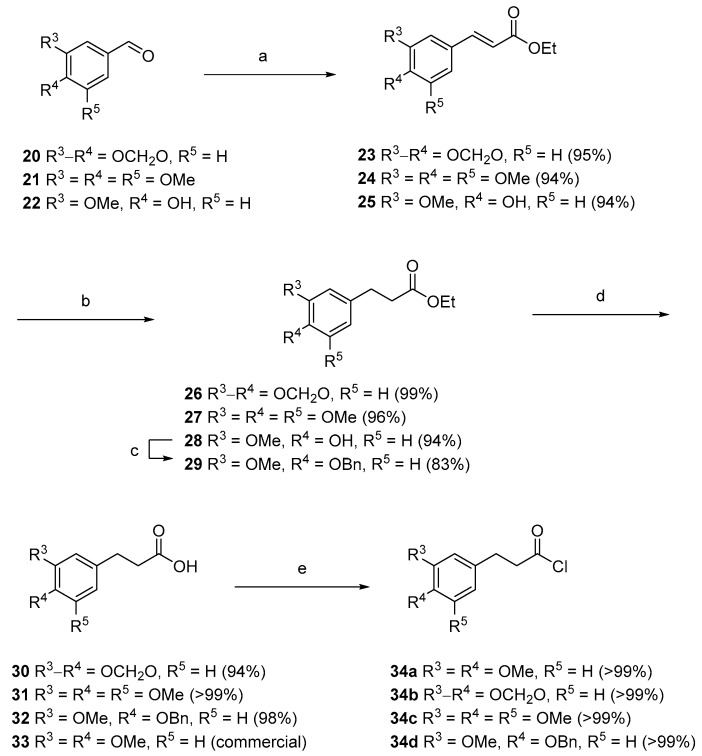
(**a**) Ph_3_PCHCO_2_Et (1.1 eq.), CH_2_Cl_2_, 3–20 h; (**b**) H_2_, Pd/C (10% *w*/*w*), ethyl acetate, 1–2 h; (**c**) BnBr, K_2_CO_3_, CH_3_CN, 65 h; (**d**) NaOH (4 eq.), MeOH/H_2_O, 2.5 h; (**e**) (COCl)_2_ (2 eq.), CH_2_Cl_2_, 1.5–4 h.

The required acid chlorides were then synthesised in four or five steps from commercially available benzaldehydes—piperonal **20**, 3,4,5-trimethoxybenzaldehyde **21** and vanillin **22** (Scheme 2). Benzaldehydes **20**–**22** first underwent a Wittig reaction with (carbethoxymethylene)triphenylphosphorane to give α,β-unsaturated esters **23**–**25** which were then hydrogenated using Pd on Carbon (10% *w*/*w*), giving saturated esters **26**–**28** in 88–94% yield over two steps. The phenol in **28** was protected as the benzyl ether, **29**, in 83% yield. Esters **26**, **27**, and **29** were hydrolysed using NaOH in methanol/water to the corresponding carboxylic acids **30**, **31**, and **32**, respectively, in 94–99% yields. Finally, chlorination of acids **30**–**32**, along with commercially available 3,4-dimethoxyphenyl propionic acid **33**, using oxalyl chloride gave acid chlorides **34a**–**d** in quantitative yields.

**Scheme 3:**
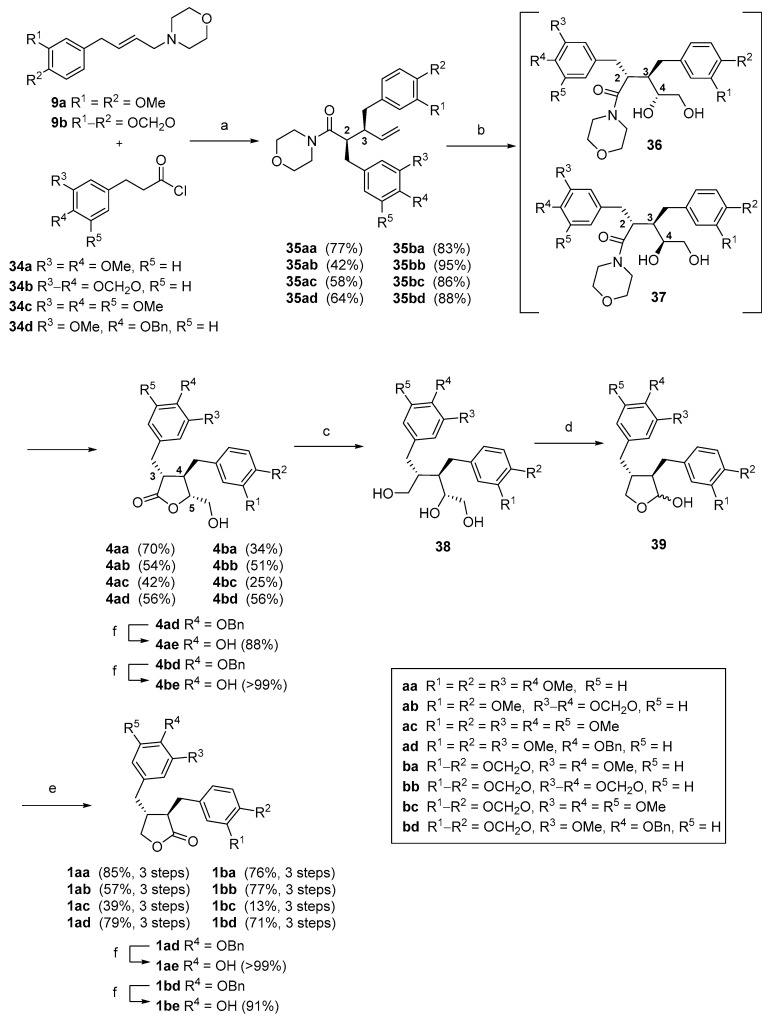
(**a**) TiCl_4_·2THF (100 mol%), *^i^*Pr_2_NEt (1.5 eq.), acid chloride (1.2 eq.), CH_2_Cl_2_, 18–24 h; (**b**) OsO_4_ (8 mol %), NMO (3 eq.), *^t^*BuOH/H_2_O (1:1), 3–7 days; (**c**) LiAlH_4_ (1.5 eq.), THF, 0.5–2 h; (**d**) NaIO_4_ (1.2 eq.), MeOH/H_2_O (3:1), 0.25–1 h; (**e**) Ag_2_CO_3_/Celite (2 eq.), toluene, reflux, 2–3 h; (**f**) H_2_, Pd/C (10% *w*/*w*), MeOH, 10 min.

Acyl-Claisen rearrangements were undertaken using two allylic morpholines **9a** and **9b** which were reacted individually with the four acid chlorides **34a**–**d**, using TiCl_4_·2THF as the Lewis acid, providing eight morpholine amides **35aa**–**bd** in 42–95% yields. All amides **35aa**–**bd** were obtained as single diastereomers with a *syn-*configuration between the C-2 and C-3 substituents (Scheme 3). All amides **35aa**–**bd** then underwent dihydroxylation using osmium tetroxide and *N*-methylporpholine *N*-oxide (NMO) to give cyclized 5-hydroxymethyllactones **4aa**–**bd**.

**Figure 3:**
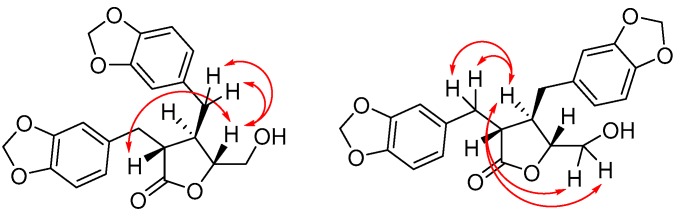
Selected NOESY correlations showing *trans,trans*-relationship of hydroxymethyl lactone lignan analogue **4bb**.

**Scheme 4:**
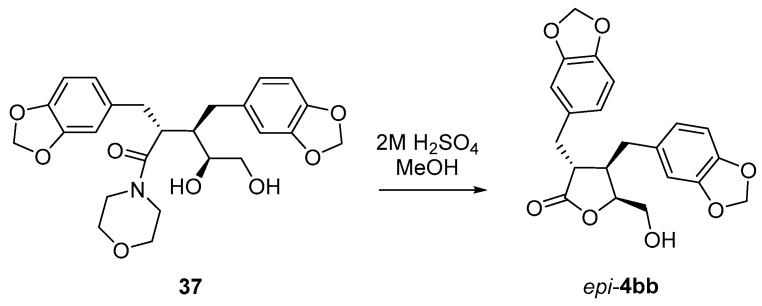
Synthesis of *epi*-**4bb**.

In all cases it was observed that only the 3,4-*trans*-4,5*-trans*-lactone was obtained. This configuration was confirmed through NOESY NMR analysis, depicted in Figure 3 with **4bb**. We propose that only this isomer was obtained due to the preferential cyclisation of the 3,4-*anti* diol **36**, leaving the polar uncyclised 3,4-*syn* diols **37** which were difficult to isolate. Upon dihydroxylation of amide **35bb** at a larger scale and following isolation of lactone **4bb** by column chromatography, a small sample of the corresponding uncyclised diol **37** was able to be isolated. This diol **37** was subsequently cyclised using 2 M H_2_SO_4_ in methanol to give the corresponding C-5 epimer, *epi*-**4bb**, confirming this hypothesis (Scheme 4).

Finally, to deprotect the benzyl-protected lactones **4ad** and **4bd** to their respective alcohols, they were subjected to hydrogenolysis to give **4ae** and **4be** in excellent yields. Transformation of C-5 hydroxymethyl analogues **4** into dibenzylbutryolactone lignans **1** was achieved via reduction using LiAlH_4_, to the corresponding triols **38aa**–**bd**, followed by periodate cleavage, forming lactols **39aa**–**bd**. These lactols **39aa**–**bd** were then oxidised using Fetizon’s reagent [37,38] to give racemic samples of dibenzyl butyrolactone lignans **1aa**–**bd**, including known natural products arcitin **1aa**, bursehernin **1ab**, (3R*,4R*)-3-(3″,4″-dimethoxybenzyl)-4-(3′,4′,5′-trimethoxybenzyl)dihydrofuran-2(3*H*)-one **1ac**, kusunokinin **1ba**, hinokinin **1bb,** and isoyatein **1bc**. Additionally, phenolic lignans, buplerol **1ae**, and haplomyrfolin **1be** were produced by the debenzylation of **1ad** and **1bd**, respectively.

**Figure 4:**
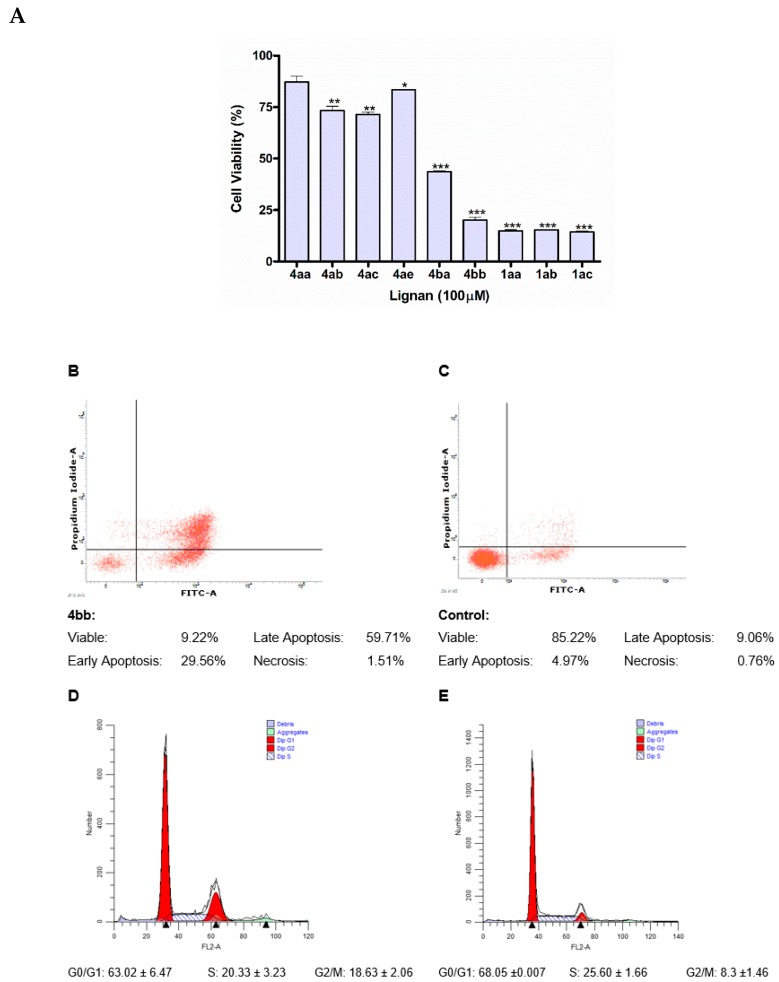
(**A**) Cell survival (by a measure of metabolic activity) of Jurkat T-cell leukaemia cells incubated with 100 µM of lignans and lignan analogues for 48 h. The data represents means of triplicate experiments and is shown as means ± SEM (*n* = 3). The positive control (not shown) had a growth of 100%. Significance of the compound activity compared to the control is expressed: (*) *p*-value <0.05; (**) *p*-value <0.01; (***) *p*-value <0.001. (**B**) Dotplot showing the viability of Jurkat T-leukaemia cells after incubation with 100 µM **4bb** for 24 h followed by labelling with annexin V/propidium iodide and analysis using flow cytometry. Cells in the bottom-left quadrant represent viable cells, bottom-right quadrant are positive for annexin V and are in early apoptosis, top-right quadrant are double positive for annexin V and propidium iodide and are in late apoptosis, and top-left quadrant are only positive for propidium iodide and are undergoing necrosis. (**C**) Negative control showing the viability of vehicle-(DMSO) treated Jurkat T-leukaemia cells. (**D**) Cell cycle analysis of unsynchronized cells incubated in the presence of 100 µM **4bb** or **E:** vehicle for 24 h. DNA content of the cells was determined by flow cytometry. Percentage of cells in each stage of the cell cycle (average of three replicates ± SD is reported).

Several of the synthesised compounds were then tested for their anti-microbial and cytotoxic activities. All tested compounds were found to be inactive against *Staphlycoccus aureus* and *Escherichia*. *coli*, showing no to little antimicrobial activity, while the compounds were shown to exhibit antiproliferative effects against Jurkat T-leukaemia cells, while also showing effects on cell cycle progression (Figure 4). While the synthesised naturally-occurring dibenzyl butyrolactones, arcitin **1aa**, bursehernin **1ab**, and (3R*,4R*)-3-(3″,4″-dimethoxybenzyl)-4-(3′,4′,5′-trimethoxybenzyl)dihydrofuran-2(3*H*)-one **1ac,** boasted the best activities, 5-hydroxymethyl analogue **4bb** had similar potency. Compound **4bb** was shown to have the best activity of all of the 5-hydroxymethyl analogues tested, inducing apoptosis, evidenced by the presence of cells in the early and predominantly in the late apoptotic cell cycle (Figure 4). Additionally the compounds demonstrated an effect on cell cycle progression. A significantly greater number of 4N cells were present following treatment with compound **4bb** in particular causing a significant increase in 4N cells (Figure 4D,E). During the cell cycle, DNA is replicated in the S-phase, going from 2N in G_1_, to 4N by the end of this phase. The DNA content in cells then remains at 4N during G_2_ and M phases, before cytokinesis at the M-phase. The observation that there was in increase in 4N cells indicates that it is likely these cells have arrested in G2/M and will not re-enter next G_1_-phase after this mitotic slippage. This is in-line with published cell cycle data following treatment with other lignans [39,40]. Furthermore, our compounds showed minimal levels of necrosis, less than 2% (except **4ba** with 7%), suggesting that the cells are in fact entering programmed cell death cycles, which is considered the most effective and non-inflammatory mechanism of cancer-cell death.

In conclusion, the synthesis of dibenzyl butyrolactone lignans utilising the acyl-Claisen rearrangement has been accomplished and represent a new, modular, and convergent method towards the synthesis of this class of natural products. Furthermore, this route gives rise to the previously-unexplored 5-hydroxymethyl derivatives **4** of these natural products. The biological activities of this new set of derivatives were assessed, with one derivative in particular, **4bb**, showing a superior cytotoxic profile and resulting in cell cycle arrest and programmed cell death of Jurkat T-leukaemia cells with less than 2% of the incubated cells entering a necrotic cell death pathway.

## 3. Experimental Section

### 3.1. General Methods

All reactions were carried out with oven-dried glassware and under a nitrogen atmosphere in dry, freshly distilled solvents unless otherwise noted. Diisopropylethylamine was distilled from CaH_2_ and stored over activated 4Å molecular sieves. All melting points for solid compounds, given in degrees Celsius (°C), were measured using a Reicher–Kofler block and are uncorrected. Infrared (IR) spectra were recorded using a Perkin Elmer Spectrum1000 FT-IR spectrometer. The NMR spectra were recorded on a 400 MHz spectrometer. Chemical shifts are reported relative to the solvent peak of chloroform (δ 7.26 for ^1^H and δ 77.16 ± 0.06 for ^13^C). The ^1^H-NMR data was reported as position (δ), relative integral, multiplicity (s, singlet; d, doublet; dd, doublet of doublets; ddd, doublet of doublet of doublets; dt, doublet of triplets; dq, doublet of quartets; t, triplet; td, triplet of doublets; q, quartet; m, multiplet), coupling constant (*J*, Hz), and the assignment of the atom. The ^13^C-NMR data were reported as position (δ) and assignment of the atom. The NMR assignments were performed using COSY, HSQC and HMBC experiments. High-resolution mass spectroscopy (HRMS) was carried out by electrospray ionization (ESI) on a MicroTOF-Q mass spectrometer. Fetizon’s reagent was prepared following a literature procedure [41]. Unless noted, chemical reagents were used as purchased.

### 3.2. Synthetic Methods

#### 3.2.1. General Procedure A: Acyl-Claisen

To a stirred suspension of TiCl_4_·2THF (1 mmol) in CH_2_Cl_2_ (5 mL), under an atmosphere of nitrogen, was added a solution of allylic morpholine (1 mmol) in CH_2_Cl_2_ (2.5 mL) followed by dropwise addition of *^i^*Pr_2_NEt (1.5 mmol). After stirring for 10 min a solution of acid chloride (1.2 mmol) in CH_2_Cl_2_ (2.5 mL) was added dropwise and the resultant mixture stirred for the specified time. The reaction mixture was quenched with aqueous NaOH (12 mL, 1 M) and the aqueous phase extracted with CH_2_Cl_2_ (3 × 10 mL). The combined organic extracts were washed with brine (6 mL), dried (MgSO_4_), the solvent removed in vacuo and the crude product purified by column chromatography.

#### 3.2.2. General Procedure B: Dihydroxylation

To a stirred solution of morpholine pentenamide (1 mmol) in *^t^*BuOH/H_2_O (1:1, 20 mL) or *^t^*BuOH/H_2_O/THF (1:1:1, 30 mL) was added NMO (3 mmol). A solution of OsO_4_ (0.08 mmol, 2.5% *w*/*v* in *^t^*BuOH) was then added dropwise and the resultant mixture stirred for the specified time. The mixture was quenched with saturated aqueous Na_2_SO_3_ (30 mL) and stirred for a further 1 h. The aqueous phase was extracted with ethyl acetate (3 × 20 mL), the combined organic extracts washed with aqueous KOH (5 mL, 1 M), dried (MgSO_4_), the solvent removed in vacuo and the crude product purified by column chromatography.

#### 3.2.3. General Procedure C: Lithium Aluminum Hydride Reduction

To a stirred suspension of LiAlH_4_ (1.4 mmol) in THF (10 mL), under an atmosphere of nitrogen at 0 °C, was added a solution of lactone (1 mmol) in THF (10 mL) and the mixture stirred for the specified time. After warming to room temperature, the mixture was quenched with the addition of water (30 mL) and the aqueous phase extracted with ethyl acetate (3 × 40 mL). The combined organic extracts were washed with brine (25 mL), dried (MgSO_4_), and the solvent removed in vacuo.

#### 3.2.4. General Procedure D: Periodate Cleavage

To a stirred solution of triol (1 mmol) in MeOH/H_2_O (3:1, 50 mL) was added NaIO_4_ (1.2 mmol) and the resultant mixture stirred for the specified time. The reaction mixture was quenched with brine (40 mL) and extracted with ethyl acetate (3 × 80 mL). The organic layers were combined, washed with water (2 × 40 mL), dried (MgSO_4_), and solvent removed in vacuo to give the crude product which was purified by column chromatography if necessary.

#### 3.2.5. General Procedure E: Fétizon’s Oxidation

To a stirred solution of lactol (1 mmol) in toluene (60 mL), under an atmosphere of nitrogen, was added Fétizon′s reagent (2 mmol) and heated at reflux for the specified time. The reaction mixture was allowed to cool and filtered, the solvent removed in vacuo and the crude product purified by column chromatography.

#### 3.2.6. General Procedure F: Benzyl Deprotection

To a stirred solution of benzyl ether (1 mmol) in MeOH (30 mL) was added 10% palladium on carbon (20% *w*/*w*) and the resultant mixture stirred under and atmosphere of hydrogen for the specified time. The reaction mixture was filtered through celite, washed with methanol (3 × 20 mL), the solvent removed in vacuo and the crude product purified by column chromatography if necessary (The ^1^H and ^13^C-NMR spectra of compounds in the Supplemental Materials).

*(E)-Ethyl 4-(3′,4′-dimethoxyphenyl)but-2-enoate* (**16**). To a stirred solution of NMO (7.9 g, 67.3 mmol) in H_2_O/*^t^*BuOH (1:1, 80 mL) was added 4-allyl-1,2-dimethoxybenzene **10** (3.86 mL, 22.4 mmol). A solution of OsO_4_ (0.6 mL, 0.059 mmol, 2.5% *w*/*v* in *^t^*BuOH) was then added dropwise and the resulting mixture stirred at room temperature for 4 days. The mixture was then quenched with saturated aqueous Na_2_SO_3_ (100 mL) and stirred for 1 h. The mixture was extracted with ethyl acetate (3 × 50 mL), the organic layers combined, washed with aqueous KOH (1 M, 20 mL), and dried (MgSO_4_). Solvent was removed in vacuo to give **12** (4.8 g, quant.) as a white solid which was used without further purification. To a stirred solution of diol **12** (4.8 g, 22.8 mmol) in methanol/H_2_O (3:1, 100 mL) was added NaIO_4_ (5.9 g, 27.4 mmol) and stirred for 30 min. The reaction mixture was then quenched with addition of brine (50 mL) and extracted with ethyl acetate (3 × 40 mL). The organic extracts were combined, washed with water (2 × 20 mL), and dried (MgSO_4_). Solvent was removed in vacuo to give **14** (2.68 g, 65%) as a pale-yellow oil which was used without further purification. To a stirred solution of 2-(3,4-dimethoxyphenyl)acetaldehyde **14** (2.68 g, 14.8 mmol) in CH_2_Cl_2_ (100 mL), under an atmosphere of nitrogen, was added (carbethoxymethylene)triphenylphosphorane (5.7 g, 16.3 mmol) and the resulting mixture stirred for 16 h. Solvent was removed in vacuo and the crude product purified by column chromatography (3:1, hexanes, ethyl acetate) to give the title compound **16** (3.13 g, 84%) as a colourless oil. R_f_ = 0.56 (2:1 hexanes, ethyl acetate). δ_H_ (400 MHz; CDCl_3_) 1.27 (3H, t, *J* = 7.2 Hz, 1-OCH_2_C*H*_3_), 3.45 (2H, dd, *J* = 1.5, 6.7 Hz, 4-H), 3.86 (6H, s, 3′, 4′-H), 4.17 (2H, q, *J* = 7.2 Hz, 1-OC*H*_2_CH_3_), 5.80 (1H, td, *J* = 1.6, 15.5 Hz, 2-H), 6.67 (1H, d, *J* = 1.9 Hz, 2′-H), 6.71 (1H, dd, *J* = 1.9, 8.1 Hz, 6′-H), 6.81 (1H, d, *J* = 8.1 Hz, 5′-H), 7.07 (1H, td, *J* = 6.7, 15.5 Hz, 3-H). δ_C_ (100 MHz; CDCl_3_) 14.3 (1-OCH_2_*C*H_3_), 38.1 (C-4), 55.9, 56.0 (3′, 4′-OCH_3_), 60.3 (1-OCH_2_CH_3_), 111.5 (C-5′), 112.1 (C-2′), 120.8 (C-6′), 122.2 (C-2), 130.2 (C-1′), 147.6 (C-3), 147.9 (C-4′), 149.1 (C-3′), 166.6 (C-1). Values are in agreement with literature data [42].

*(E)-4-(3′,4′-Dimethoxyphenyl)but-2-en-1-ol* (**18**). To a stirred solution of ester **16** (1.0 g, 4.0 mmol) in CH_2_Cl_2_ (20 mL), under an atmosphere of nitrogen at −78 °C, was added DIBAL (12 mL, 1 M in cyclohexane) and the resulting mixture stirred for 10 min. The reaction mixture was quenched with addition of 2 M HCl until gas evolution ceased, the organic phase separated and the aqueous phase further extracted with CH_2_Cl_2_ (3 × 10 mL). The organic layers were combined then washed with water (10 mL) and dried (MgSO_4_). Solvent was removed in vacuo and the crude product purified by column chromatography (1:1 hexanes, ethyl acetate) to give the title compound **18** (0.76 g, 92%) as a colourless oil. R_f_ = 0.18 (2:1, hexanes, ethyl acetate). δ_H_ (400 MHz; CDCl_3_) 3.30 (2H, d, *J* = 6.6 Hz, 4-H), 3.82 (3H, s, 4′-OCH_3_), 3.83 (3H, s, 3′-OCH_3_), 4.08 (2H, d, *J* = 5.6 Hz, 1-H), 5.64–5.69 (1H, m, 2-H), 5.78–5.83 (1H, m, 3-H), 6.68 (1H, s, 2′-H), 6.69 (1H, d, *J* = 8.0 Hz, 6′-H), 6.77 (1H, d, *J* = 8.0 Hz, 5′-H). δ_C_ (100 MHz; CDCl_3_) 38.2 (C-4), 55.8 and 55.9 (3′ and 4′-OCH_3_), 63.3 (C-1), 111.4 (C-5′), 112.0 (C-2′), 120.4 (C-6′), 130.2 (C-2), 131.6 (C-3), 132.7 (C-1′), 147.4 (C-4′), 148.9 (C-3′). IR: ν_MAX_ (film)/cm^-1^; 3391 (broad), 2933, 2835, 1591, 1512, 1463, 1417, 1258, 1232, 1137, 1025, 971, 852, 806, 762. HRMS (ESI^+^) Found [M + Na]^+^ 231.0995; C_12_H_16_NaO_3_ requires 231.0992.

*(E)-4-(4-(3′,4′-Dimethoxyphenyl)but-2-en-1-yl)morpholine* (**9a**). To a stirred solution of alcohol **18** (0.73 g, 3.5 mmol) in CH_2_Cl_2_ (20 mL), under an atmosphere of nitrogen at 0 °C, was added Et_3_N (1.5 mL, 10.5 mmol) and stirred for 5 min. MsCl (0.48 mL, 4.2 mmol) was added and stirred for 10 min. Morpholine (0.50 mL, 5.3 mmol) was added and the mixture brought to room temperature and stirred for 2 h. Saturated aqueous NaHCO_3_ (20 mL) and water (4 mL) was then added and the aqueous layer further extracted with CH_2_Cl_2_ (3 × 20 mL). The organic layers were then combined, dried (MgSO_4_) and the solvent removed in vacuo. The crude product was purified by column chromatography (1:1 hexanes, ethyl acetate) to give the title compound 9a (0.60 g, 62%) as a colourless oil. R_f_ = 0.31 (1:2 hexanes, ethyl acetate). δ_H_ (400 MHz; CDCl_3_) 2.41–2.44 (4H, m, O(CH_2_C*H*_2_)_2_N), 2.96 (2H, d, *J* = 6.8 Hz, 1-H), 3.30 (2H, d, *J* = 6.7 Hz, 4-H), 3.68–3.71 (4H, m, O(C*H*_2_CH_2_)_2_N), 3.83 (6H, s, 3′, 4′-OCH_3_), 5.52–5.57 (1H, m, 3-H), 5.71–5.78 (1H, m, 2-H), 6.67–6.70 (2H, m, 2′ and 6′-H), 6.78 (1H, d, *J* = 7.9 Hz, 5′-H). δ_C_ (100 MHz; CDCl_3_) 38.5 (C-4), 53.6 (O(CH_2_*C*H_2_)_2_N), 55.8, 56.0 (3′, 4′-OCH_3_), 61.1 (C-1), 67.0 (O(*C*H_2_CH_2_)_2_N), 111.4 (C-5′), 111.9 (C-2′), 120.3 (C-6′), 127.1 (C-3), 132.8 (C-1′), 133.8 (C-2), 147.5 (C-4′), 149.0 (C-3′). IR: ν_MAX_ (film)/cm^−1^; 2934, 2851, 1591, 1453, 1260, 1138, 1028, 976, 864, 805, 763. HRMS (ESI^+^) Found [M + H]^+^ 278.1762; C_16_H_24_NO_3_ requires 278.1751.

*(E)-Ethyl 4-(3′,4′-methylenedioxyphenyl)but-2-enoate* (**17**). To a stirred solution of NMO (8.67 g, 74.0 mmol) in H_2_O/*^t^*BuOH (1:1, 80 mL) was added safrole **11** (4.0 mL, 27 mmol). A solution of OsO_4_ (0.75 mL, 0.074 mmol, 2.5% *w*/*v* in *^t^*BuOH) was added dropwise and the resultant mixture stirred at room temperature for 17 h. The reaction mixture was quenched with saturated aqueous Na_2_SO_3_ (100 mL) and stirred for 1 h. The mixture was extracted with ethyl acetate (3 × 50 mL), the organic layers were combined, washed with aqueous KOH (1 M, 20 mL) and dried (MgSO_4_). Solvent was removed in vacuo to give diol **13** (5.2 g, quant.) as a white solid which was used without further purification. To a stirred solution of diol **13** (5.2 g, 27 mmol) in methanol/H_2_O (3:1, 100 mL) was added NaIO_4_ (6.8 g, 32 mmol) and stirred for 2 h. The mixture was then quenched with addition of brine (50 mL) and extracted with ethyl acetate (3 × 50 mL). The organic extracts were combined, washed with water (2 × 20 mL), brine (10 mL), and dried (MgSO_4_). Solvent was removed in vacuo to give aldehyde **15** (4.4 g, quant.) as a yellow oil which was used without further purification. To a stirred solution of 2-(3,4-methylenedioxyphenyl)acetaldehyde **15** (4.4 g, 27 mmol) in CH_2_Cl_2_ (50 mL), under an atmosphere of nitrogen, was added (carbethoxymethylene)triphenylphosphorane (10.4 g, 30 mmol) and the resulting mixture stirred for 16 h. Solvent was removed in vacuo and the crude product purified by column chromatography (19:1, hexanes, ethyl acetate) to give the title compound 17 (3.54 g, 56%) as a colourless oil. R_f_ = 0.73 (2:1 hexanes, ethyl acetate). δ_H_ (400 MHz; CDCl_3_) 1.27 (3H, t, *J* = 7.2 Hz, 1-OCH_2_C*H*_3_), 3.42 (2H, dd, *J* = 6.6, 1.6 Hz, 4-H), 4.17 (2H, q, *J* = 7.2 Hz, 1-OC*H*_2_CH_3_), 5.78 (1H, dt, *J* = 15.5, 1.6 Hz, 2-H), 5.93 (2H, s, OCH_2_O), 6.61 (1H, dd, *J* = 8.0, 2.0 Hz, 6′-H), 6.64 (1H, d, *J* = 2.0 Hz, 2′-H), 6.74 (1H, d, *J* = 8.0 Hz, 5′-H), 7.04 (1H, dt, *J* = 15.5, 6.6 Hz, 3-H). δ_C_ (100 MHz; CDCl_3_) 14.4 (1-OCH_2_*C*H_3_), 38.2 (C-4), 60.4 (1-O*C*H_2_CH_3_), 101.1 (OCH_2_O), 108.5 (C-5′), 109.4 (C-2′), 121.9 (C-6′), 122.4 (C-2), 131.5 (C-1′), 146.5 (C-4′), 147.5 (C-3), 148.0 (C-3′), 166.6 (C-1). Values are in agreement with literature data [43].

*(E)-4-(3′,4′-Methylenedioxyphenyl)but-2-en-1-ol* (**19**). To a stirred solution of ester **17** (3.2 g, 13.7 mmol) in toluene (100 mL), under an atmosphere of nitrogen at −10 °C, was added DIBAL (30 mL, 1 M in toluene) and the resultant mixture stirred for 10 min. The reaction mixture was quenched with addition of 2 M HCl until gas evolution ceased, the organic layer was separated and the aqueous phase further extracted with CH_2_Cl_2_ (3 × 50 mL). The organic layers were combined, washed with brine (30 mL) and dried (MgSO_4_). Solvent was removed in vacuo and the crude product purified by column chromatography (3:1 hexanes, ethyl acetate) to give the title compound **19** (2.59 g, 98%) as a pale yellow oil. R_f_ = 0.42 (hexanes, ethyl acetate). δ_H_ (400 MHz; CDCl_3_) 1.41 (1H, br s, 1-OH), 3.30 (2H, d, *J* = 6.6 Hz, 4-H), 4.12 (2H, br d, *J* = 4.5 Hz, 1-H), 5.64–5.72 (1H, m, 2-H), 5.77–5.85 (1H, m, 3-H), 5.92 (2H, s, OCH_2_O), 6.63 (1H, dd, *J* = 7.9, 1.9 Hz, 6′-H), 6.67 (1H, d, *J* = 1.9 Hz, 2′-H), 6.73 (1H, d, 7.9 Hz, 5′-H). δ_C_ (100 MHz; CDCl_3_) 38.4 (C-4), 63.6 (C-1), 101.0 (OCH_2_O), 108.3 (C-5′), 109.2 (C-2′), 121.4 (C-6′), 130.4, 131.8 (C-2, 3), 133.9 (C-1′), 146.0, 147.8 (C-3′, 4′). Values are in agreement with literature data [43].

*(E)-4-(4-(3′,4′-Methylenedioxyphenyl)but-2-en-1-yl)morpholine* (**9b**). To a stirred solution of alcohol **19** (1.66 g, 8.6 mmol) in CH_2_Cl_2_ (15 mL), under an atmosphere of nitrogen at 0 °C, was added Et_3_N (3.6 mL, 25.9 mmol) and stirred for 5 min. MsCl (1.2 mL, 10.4 mmol) was added and stirred for 10 min. Morpholine (1.3 mL, 13.8 mmol) was added and the mixture brought to room temperature and stirred for 18 h. Saturated aqueous NaHCO_3_ (25 mL) and water (5 mL) was added and the aqueous layer further extracted with CH_2_Cl_2_ (3 × 30 mL). The organic layers were combined, dried (MgSO_4_) and the solvent removed in vacuo. The crude product was purified by column chromatography (2:1 hexanes, ethyl acetate) to give the title compound **9b** (1.4 g, 60%) as a pale yellow oil. R_f_ = 0.39 (1:2 hexanes, ethyl acetate). δ_H_ (400 MHz; CDCl_3_) 2.43 (4H, br t, *J* = 4.7 Hz, NC*H*_2_CH_2_O), 2.96 (2H, d, *J* = 6.5 Hz, 1-H), 3.28 (2H, d, *J* = 7.0 Hz, 4-H), 3.71 (4H, t, *J* = 4.7 Hz, NCH_2_C*H*_2_O), 5.49–5.56 (1H, m, 2-H), 5.69–5.76 (1H, m, 3-H), 5.91 (2H, d, *J* = 2.0 Hz, OCH_2_O), 6.61 (1H, dd, *J* = 7.5, 2.0 Hz, 6′-H), 6.65 (1H, d, *J* = 2.0 Hz, 2′-H), 6.72 (1H, d, *J* = 7.5 Hz, 5′-H). δ_C_ (100 MHz; CDCl_3_) 38.7 (C-4), 53.7 (N*C*H_2_CH_2_O), 61.2 (C-1), 67.1 (NCH_2_*C*H_2_O), 100.9 (OCH_2_O), 108.3 (C-5′), 109.1 (C-2′), 121.4 (C-6′), 127.4 (C-2), 133.7 (C-3), 134.1 (C-1′), 146.0 (C-4′), 147.8 (C-3′). IR: ν_MAX_ (film)/cm^−1^; 2855, 1739, 1488, 1242, 1115, 1036, 926, 864, 736. HRMS (ESI^+^) Found [M + H]^+^ 262.1428; C_15_H_20_NO_3_ requires 262.1438.

*(E)-Ethyl-3-(3′,4′-methylenedioxyphenyl)prop-2-enoate* (**23**). To a stirred solution of piperonal **20** (5.0 g, 33 mmol) in CH_2_Cl_2_ (100 mL), under an atmosphere of nitrogen, was added (carbethoxymethylene)triphenylphosphorane (12.8 g, 37.0 mmol) and the resulting mixture stirred for 20 h. Solvent was then removed in vacuo and the crude product purified by column chromatography (3:1, hexanes, ethyl acetate) to give the title compound **23** (6.97 g, 95%) as a white solid. R_f_ = 0.68 (2:1 hexanes, ethyl acetate). Melting point: 62–64 °C. δ_H_ (400 MHz; CDCl_3_) 1.32 (3H, t, *J* = 7.2 Hz, 1-OCH_2_C*H*_3_), 4.25 (2H, q, *J* = 7.2 Hz, 1-OC*H*_2_CH_3_), 6.00 (2H, s, -OCH_2_O-), 6.25 (1H, d, *J* = 15.9 Hz, 2-H), 6.80 (1H, d, *J* = 8.0 Hz, 5′-H), 7.00 (1H, dd, *J* = 1.4, 8.0 Hz, 6′-H), 7.02 (1H, d, *J* = 1.4 Hz, 6′-H), 7.58 (1H, d, *J* = 15.9 Hz, 3-H). δ_C_ (100 MHz; CDCl_3_) 14.5 (1-OCH_2_*C*H_3_), 60.5 (1-OCH_2_CH_3_), 101.7 (-OCH_2_O-), 106.6 (C-5′), 108.7 (C-2′), 116.4 (C-2), 124.5 (C-6′), 129.1 (C-1′), 144.4 (C-3), 148.5 (C-4′), 149.7 (C-3′), 167.3 (C-1). Values are in agreement with literature data [44].

*(E)-Ethyl-3-(3′,4′,5′-trimethoxyphenyl)prop-2-enoate* (**24**). To a stirred solution of 3,4,5-trimethoxybenzaldehyde **21** (3.0 g, 15.3 mmol) in CH_2_Cl_2_ (100 mL), under an atmosphere of nitrogen, was added (carbethoxymethylene)triphenylphosphorane (5.9 g, 16.8 mmol) and the resulting mixture stirred for 3 h. Solvent was then removed in vacuo and the crude product purified by column chromatography (3:1, hexanes, ethyl acetate) to give the title compound **24** (4.0 g, 94%) as a white solid. R_f_ = 0.52 (2:1 hexanes, ethyl acetate). Melting point: 64–66 °C. δ_H_ (400 MHz; CDCl_3_) 1.34 (3H, t, *J* = 7.2 Hz, 1-OCH_2_C*H*_3_), 3.87 (3H, s, 4′-OCH_3_), 3.88 (6H, s, 3′-OCH_3_), 4.26 (2H, q, *J* = 7.2 Hz, 1-OC*H*_2_CH_3_), 6.34 (1H, d, *J* = 15.9 Hz, 2-H), 6.75 (2H, s, 2′-H), 7.60 (1H, d, *J* = 15.9 Hz, 3-H). δ_C_ (100 MHz; CDCl_3_) 14.5 (1-OCH_2_*C*H_3_), 56.3 (3′-OCH_3_), 60.6 (1-OCH_2_CH_3_), 61.1 (4′-OCH_3_), 105.3 (C-2′), 117.7 (C-2), 130.1 (C-1′), 140.2 (C-4′), 144.7 (C-3), 153.6 (C-3′), 167.1 (C-1). Values are in agreement with literature data [45].

*3-(3′,4′,5′-Trimethoxyphenyl)propionic acid* (**31**). To a stirred solution of **24** (5.4 g, 19.4 mmol) in ethyl acetate (30 mL) was added 10% palladium on activated carbon (0.54 g, 10% *w*/*w*). The solution was flushed with an atmosphere of hydrogen and stirred for 2 h. The reaction mixture was then filtered through a plug of celite and washed with ethyl acetate, solvent was then removed in vacuo to give saturated ester **27** (5.23 g, 96%) which was then used without further purification.

To a stirred solution of ester **27** (5.1 g, 17.9 mmol) in methanol (30 mL) was added aqueous NaOH (72 mL, 1 M, 4 eq.) and stirred for 20 min. The mixture was then extracted with CH_2_Cl_2_ (10 mL) and the aqueous layer acidified with aqueous 2 M HCl. The aqueous phase was then extracted with ethyl acetate (3 × 50 mL), dried (MgSO_4_) and solvent removed in vacuo to give the title compound **31** (4.6 g, quant.) as a white solid. R_f_ = 0.15 (2:1 hexanes, ethyl acetate). Melting point: 104–105 °C. δ_H_ (400 MHz; CDCl_3_) 2.68 (2H, t, *J* = 7.8 Hz, 2-H), 2.90 (2H, t, *J* = 7.8 Hz, 3-H), 3.82 (3H, s, 4′-OCH_3_), 3.84 (6H, s, 3′-OCH_3_), 6.43 (2H, s, 2′-H). δ_C_ (100 MHz; CDCl_3_) 31.1 (C-2), 35.8 (C-3), 56.2 (3′-OCH_3_), 61.0 (4′-OCH_3_), 105.4 (C-2′), 136.0 (C-1′), 136.7 (C-4′), 153.4 (C-3′), 178.8 (C-1). Values are in agreement with literature data [46].

*3-(3′,4′-Methylenedioxyphenyl)propionic acid* (**30**). To a stirred solution of **23** (6.92 g, 31.4 mmol) in ethyl acetate (30 mL) was added 10% palladium on activated carbon (0.69 g, 10% *w/w*). The solution was flushed with an atmosphere of hydrogen and stirred for 1 h. The reaction mixture was then filtered through a plug of celite and washed with ethyl acetate, solvent was then removed in vacuo to give saturated ester **26** (6.9 g, 99%) which was then used without further purification.

To a stirred solution of ester **26** (6.74 g, 30.0 mmol) in methanol (30 mL) was added aqueous NaOH (121 mL, 1 M, 4 eq.) and stirred for 2.5 h. The mixture was then extracted with ethyl acetate (10 mL) and the aqueous layer acidified with aqueous 2 M HCl. The aqueous phase was then extracted with ethyl acetate (3 × 50 mL), dried (MgSO_4_) and solvent removed in vacuo to give the title compound **30** (5.5 g, 94%) as a white solid. R_f_ = 0.44 (2:1 hexanes, ethyl acetate). Melting point: 80–82°C. δ_H_ (400 MHz; CDCl_3_) 2.64 (2H, t, *J* = 7.7 Hz, 2-H), 2.88 (2H, t, *J* = 7.7 Hz, 3-H), 5.93 (2H, s, -OCH_2_O-), 6.66 (1H, dd, *J* = 7.9, 1.4 Hz, 6′-H), 6.70 (1H, d, *J* = 1.4 Hz, 2′-H), 6.74 (1H, d, *J* = 7.9 Hz, 5′-H). δ_C_ (100 MHz; CDCl_3_) 30.5 (C-2), 36.1 (C-3), 101.0 (-OCH_2_O-), 108.4 (C-2′), 108.9 (C-5′), 121.2 (C-6′), 134.1 (C-1′), 146.2 (C-3′), 147.8 (C-4′), 179.1 (C-1). Values are in agreement with literature data [47].

*3-(3′-Methoxy-4′-benzyloxyphenyl)propionic acid* (**32**). To a stirred solution of vanillin **22** (3.0 g, 19.7 mmol) in CH_2_Cl_2_ (100 mL), under an atmosphere of nitrogen, was added (carbethoxymethylene)triphenylphosphorane (7.56 g, 21.7 mmol) and the resulting mixture stirred for 18 h. Solvent was then removed in vacuo and the crude product purified by column chromatography (2:1, hexanes, ethyl acetate) to give a 2:1 mixture of E and Z isomers of unsaturated ester **25** (4.13 g, 94%) as a yellow oil which was used immediately.

To a stirred solution of unsaturated ester **25** (4.13 g, 18.6 mmol) in ethyl acetate (30 mL) was added 10% palladium on activated carbon (0.4 g, 10% *w*/*w*). The solution was flushed with an atmosphere of hydrogen and stirred for 2 h. The reaction mixture was then filtered through a plug of celite and washed with ethyl acetate, solvent was then removed in vacuo to give saturated ester **28** (3.9 g, 94%) as a yellow oil which was then used without further purification. To a stirred solution of phenol **28** (3.75 g, 16.7 mmol) in acetonitrile (40 mL), under an atmosphere of nitrogen, was added K_2_CO_3_ (6.9 g, 50.0 mmol) and stirred for 10 min. Benzyl bromide (6.0 mL, 50.0 mmol) was then added and the resulting mixture allowed to stir for 65 h. The reaction mixture was then quenched with addition of water (50 mL) and extracted with CH_2_Cl_2_ (3 × 30 mL). The organic phases were combined, washed with water (2 × 10 mL) and dried (MgSO_4_). Solvent was then removed in vacuo and the crude product purified by column chromatography (9:1 hexanes, ethyl acetate) to give benzyl ether **29** (4.38 g, 83%) as a colourless oil which was used immediately. To a stirred solution of ester **29** (4.3 g, 13.7 mmol) in methanol (30 mL) was added aqueous NaOH (55 mL, 1 M, 4 eq.) and stirred for 2.5 h. The mixture was then acidified with aqueous 2 M HCl, extracted with ethyl acetate (3 × 50 mL), dried (MgSO_4_) and solvent removed in vacuo to give the title compound **32** (3.85 g, 98%) as a white solid. R_f_ = 0.30 (2:1 hexanes, ethyl acetate). Melting point: 99–100°C. δ_H_ (400 MHz; CDCl_3_) 2.66 (2H, t, *J* = 7.7 Hz, 2-H), 2.90 (2H, t, *J* = 7.7 Hz, 3-H), 3.88 (3H, s, 3′-OCH_3_), 5.13 (2H, s, 7′-H), 6.68 (1H, dd, *J* = 8.2, 2.0 Hz, 6′-H), 6.76 (1H, d, *J* = 2.0 Hz, 2′-H), 6.81 (1H, d, *J* = 8.2 Hz, 5′-H), 7.27–7.32 (1H, m, 11′-H), 7.34–7.39 (2H, m, 10′-H), 7.41–7.45 (2H, m, 9′-H). δ_C_ (100 MHz; CDCl_3_) 30.4 (C-2), 35.9 (C-3), 56.1 (3′-OCH_3_), 71.3 (C-7′), 112.4 (C-2′), 114.5 (C-5′), 120.3 (C-6′), 127.4 (C-9′), 127.9 (C-11′), 128.7 (C-10′), 133.5 (C-1′), 137.4 (C-8′), 146.9 (C-4′), 149.8 (C-3′), 178.8 (C-1). Values are in agreement with literature data [48].

*3-(3′,4′-Methylenedioxyphenyl)propanoyl chloride* (**34b**). To a stirred solution of carboxylic acid **30** (0.22 g, 1.2 mmol) in CH_2_Cl_2_ (3 mL), under an atmosphere of nitrogen, was added oxalyl chloride (0.2 mL, 2.3 mmol) dropwise and the mixture stirred for 4 h. The solvent was removed in vacuo to give the title compound **34b** (0.24 g, quant.) as a green oil, which was placed under nitrogen and used without further purification.

*3-(3′,4′-Dimethoxyphenyl)propanoyl chloride* (**34a**). To a stirred solution of carboxylic acid **33** (0.24 g, 1.2 mmol) in CH_2_Cl_2_ (5 mL), under an atmosphere of nitrogen, was added oxalyl chloride (0.2 mL, 2.3 mmol) dropwise and the mixture stirred for 2.5 h. The solvent was removed in vacuo to give the title compound **34a** (0.26 g, quant.) as a yellow oil, which was placed under nitrogen and used without further purification.

*3-(3′,4′,5′-Trimethoxyphenyl)propanoyl chloride* (**34c**). To a stirred solution of carboxylic acid **31** (0.25 g, 1.2 mmol) in CH_2_Cl_2_ (3 mL), under an atmosphere of nitrogen, was added oxalyl chloride (0.2 mL, 2.3 mmol) dropwise and the mixture stirred for 1.5 h. The solvent removed in vacuo to give the title compound **34c** (0.27 g, quant.) as a green crystalline solid, which was placed under nitrogen and used without further purification.

*3-(3′,4′-Methylenedioxyphenyl)propanoyl chloride* (**34d**). To a stirred solution of carboxylic acid **32** (0.33 g, 1.2 mmol) in CH_2_Cl_2_ (3 mL), under an atmosphere of nitrogen, was added oxalyl chloride (0.2 mL, 2.3 mmol) dropwise and the mixture stirred for 4 h. The solvent was removed in vacuo to give the title compound **34d** (0.35 g, quant.) as a yellow oil, which was placed under nitrogen and used without further purification.

(2R*,3S*)-2-(3′,4′-Methylenedioxybenzyl)-3-(3″,4″-dimethoxybenzyl)-1-morpholinopent-4-en-1-one (**35ab**). Using general procedure A: Morpholine **9a** (0.57 g, 2.06 mmol), acid chloride **34b** (0.52 g, 2.47 mmol) and reaction time of 24 h. The crude product was purified by column chromatography (2:1 hexanes, ethyl acetate) to give the title compound **35ab** (0.39 g, 42%) as a pale-yellow amorphous solid. R_f_ = 0.58 (1:3, hexanes, ethyl acetate). Melting point: 114–116 °C. δ_H_ (400 MHz; CDCl_3_) 2.57 (1H, dd, *J* = 13.6, 9.0 Hz, 7″-H_A_), 2.66–2.73 (1H, m, 3-H), 2.77–2.85 (2H, m, 7′-H_A_, OC*H_A_*CH_2_N), 2.85–2.94 (4H, m, 2-H, 7′-H_B_, 7″-H_B_, OCH_2_C*H_A_*N), 3.06 (1H, ddd, *J* = 13.3, 7.9, 3.3 Hz, OCH_2_C*H_B_*N), 3.27–3.41 (3H, m, OC*H_C_*C*H_C_*N, OC*H_B_*CH_2_N), 3.53–3.60 (1H, m, O*H_D_*CH_2_N), 3.67–3.75 (1H, m, OCH_2_C*H_D_*N), 3.85 (3H, s, 4″-OCH_3_), 3.86 (3H, s, 3″-OCH_3_), 4.88 (1H, dd, *J* = 16.9, 1.8 Hz, 5-H_A_), 4.98 (1H, dd, *J* = 10.3, 1.8 Hz, 5-H_B_), 5.85 (1H, ddd, *J* = 16.9, 10.3, 9.5 Hz, 4-H), 5.90 (1H, d, *J* = 1.3 Hz, OCH_A_O), 5.91 (1H, d, *J* = 1.3 Hz, OCH_B_O), 6.60 (1H, dd, *J* = 7.8, 1.6 Hz, 6′-H), 6.64 (1H, d, *J* = 1.6 Hz, 2′-H), 6.65–6.68 (2H, m, 2″, 6″-H), 6.70 (1H, d, *J* = 7.8 Hz, 5′-H), 6.77 (1H, d, *J* = 8.7 Hz, 5″-H). δ_C_ (100 MHz; CDCl_3_) 37.4 (C-7′), 38.3 (C-7″), 42.0 (OCH_2_*C*H_CD_N), 46.4 (OCH_2_*C*H_AB_N), 46.6 (C-2), 48.5 (C-3), 56.0 (3′, 4′-OCH_3_), 66.4 (O*C*H_AB_CH_2_N), 67.0 (O*C*H_CD_CH_2_N), 101.0 (OCH_2_O), 108.4 (C-5′), 109.6 (C-2′), 111.1 (C-5″), 112.4 (C-2″), 116.8 (C-5), 121.3 (C-6″), 122.0 (C-6′), 132.3 (C-1″), 133.6 (C-1′), 139.3 (C-4), 146.2 (C-4′), 147.5 (C-4″), 147.7 (C-3′), 148.9 (C-3″), 172.6 (C-1). IR: ν_MAX_ (film)/cm^-1^; 2963, 1631, 1515, 1488, 1442, 1236, 1031, 925, 807, 730. HRMS (ESI^+^) Found [M + H]^+^ 454.2241; C_26_H_32_NO_6_ requires 454.2224.

(2R*,3S*)-2-(3′,4′,5′-Trimethoxybenzyl)-3-(3″,4″-dimethoxybenzyl)-1-morpholinopent-4-en-1-one (**35ac**). Using general procedure A: Morpholine **9a** (0.47 g, 1.7 mmol), acid chloride **34c** (0.53 g, 2.0 mmol) and a reaction time of 19 h. The crude product was purified by column chromatography (1:1 hexanes, ethyl acetate) to give the title compound **35ac** (0.50 g, 58%) as a yellow oil. R_f_ = 0.38 (1:3 hexanes, ethyl acetate). δ_H_ (400 MHz; CDCl_3_) 2.59 (1H, dd, *J* = 13.6, 9.2 Hz, 7″-H_A_), 2.67–2.74 (1H, m, 3-H), 2.78 (1H, ddd, *J* = 11.4, 7.8, 3.0 Hz, NCH_2_C*H_A_*O), 2.82–2.96 (5H, m, 2-H, 7′-H, 7″-H_B_, NC*H_A_*CH_2_O), 3.06 (1H, ddd, *J* = 13.2, 7.8, 3.0 Hz, NC*H_B_*CH_2_O), 3.25–3.40 (3H, m, NC*H_B_*CH_2_O, NC*H_C_*C*H_C_*O), 3.54–3.61 (1H, m, NC*H_D_*CH_2_O), 3.67–3.73 (1H, m, NCH_2_C*H_D_*O), 3.80 (3H, s, 4′-OCH_3_), 3.82 (6H, s, 3′-OCH_3_), 3.85 (3H, s, 4″-OCH_3_), 3.86 (3H, s, 3″-OCH_3_), 4.90 (1H, dd, *J* = 17.0, 1.8 Hz, 5-H_A_), 5.00 (1H, dd, *J* = 10.2, 1.8 Hz, 5-H_B_), 5.87 (1H, ddd, *J* = 17.0, 10.2, 9.1 Hz, 4-H), 6.37 (2H, s, 2′-H), 6.66–6.70 (2H, m, 2″, 6″-H), 6.78 (1H, d, *J* = 8.7 Hz, 5″-H). δ_C_ (100 MHz; CDCl_3_) 38.1 (C-7′), 38.3 (C-7″), 42.0 (N*C*H_CD_CH_2_O), 46.4 (N*C*H_AB_CH_2_O), 46.5 (C-2), 48.7 (C-3), 56.0 (3″, 4″-OCH_3_), 56.3 (3′-OCH_3_), 61.0 (4′-OCH_3_), 66.4 (NCH_2_*C*H_AB_O), 66.9 (NCH_2_*C*H_CD_O), 106.2 (C-2′), 111.1 (C-5″), 112.5 (C-2″), 116.8 (C-5), 121.2 (C-6″), 132.3 (C-1″), 135.6 (C-1′), 136.8 (C-4′), 139.2 (C-4), 147.5 (C-4″), 148.8 (C-3″), 153.3 (C-3′), 172.6 (C-1). IR: ν_MAX_ (film)/cm^-1^; 2940, 1632, 1589, 1459, 1236, 1123, 1028, 913, 735. HRMS (ESI^+^) Found [M + Na]^+^ 522.2474; C_28_H_37_NNaO_7_ requires 522.2462.

(2R*,3S*)-2-(3′,4′-Dimethoxybenzyl)-3-(3″,4″-dimethoxybenzyl)-1-morpholinopent-4-en-1-one (**35aa**). Using general procedure A: Morpholine **9a** (0.53 g, 1.91 mmol), acid chloride **34a** (0.52 g, 2.29 mmol) and a reaction time of 24 h. The crude product was purified by flash chromatography (1:3 hexanes, ethyl acetate) to give the title compound **35aa** (0.63 g, 77% yield) as a pale-yellow amorphous solid. R_f_ = 0.42 (19:1 CH_2_Cl_2_, methanol). Melting point: 98–101 °C. δ_H_ (400 MHz; CDCl_3_) 2.55–2.63 (1H, m, 7″-H_A_), 2.85–2.93 (1H, m, 7″-H_B_), 2.67–2.85 (3H, m, 3-H, OCH_2_C*H*_AB_N), 3.29-3.37 (4H, m, OCH_2_C*H*_CD_N, OC*H*_AB_CH_2_N), 2.85–3.06 (3H, m, 2-H, 7′-H), 3.50–3.67 (2H, m, OC*H*_CD_CH_2_N), 3.83, 3.84, 3.85, 3.86 (12H, s, 3′, 4′, 3″, 4″-OCH_3_), 4.89 (1H, dd, *J* = 17.1, 1.7 Hz, 5-H), 4.99 (1H, dd, *J* = 10.3, 1.9 Hz, 5-H), 5.82–5.91 (1H, m, 4-H), 6.67–6.69 (4H, m, 2′, 6′, 2″, 6″-H), 6.75–6.78 (2H, m, 5′, 5″-H). δ_C_ (100 MHz; CDCl_3_) 37.2 (C-2), 38.2 (C-7″), 41.9, 46.5 (OCH_2_*C*H_2_N), 46.2 (C-7′), 48.5 (C-3), 55.8, 55.9 (3′, 4′, 3″, 4″-OCH_3_), 66.3, 66.8 (O*C*H_2_CH_2_N), 111.0, 111.3 (C-5′, 5″), 112.4, 112.6 (C-2′, 2″), 116.6 (C-5), 120.9, 121.2 (C-6′, 6″), 132.2, 132.3 (C-1′, 1″), 139.2 (C-4), 147.3, 147.6 (4′, 4″-OCH_3_), 148.7, 148.8 (3′, 3″-OCH_3_), 172.6 (C-1). IR: ν_MAX_ (film)/cm^−1^; 2935, 1628, 1591, 1462, 1260, 1155, 1027, 912, 857, 765. HRMS (ESI^+^) Found [M + H]^+^ 470.2537; C_27_H_36_NO_6_ requires 470.2537

(2R*,3S*)-2-(3′-Methoxy-4′-benzyloxybenzyl)-3-(3″,4″-dimethoxybenzyl)-1-morpholino-pent-4-en-1-one (**35ad**). Using general procedure A: Morpholine **9a** (0.47 g, 1.7 mmol), acid chloride **34d** (0.62 g, 2.0 mmol) and a reaction time of 22 h. The crude product was purified by column chromatography (2:1 hexanes, ethyl acetate) to give the title compound **35ad** (0.59 g, 64%) as a yellow oil.

R_f_ = 0.58 (1:3, hexanes, ethyl acetate). δ_H_ (400 MHz; CDCl_3_) 2.57 (1H, dd, *J* = 13.5, 9.0 Hz, 7″-H_A_), 2.62–2.68 (1H, m, 3-H), 2.68–2.74 (1H, m, OC*H_A_*CH_2_N), 2.75–2.82 (1H, m, OCH_2_C*H_A_*N), 2.83–2.92 (4H, m, 2-H, 7′-H, 7″-H_B_), 2.99 (1H, ddd, *J* = 13.3, 7.6, 3.2 Hz, OCH_2_C*H_B_*N), 3.20–3.32 (3H, m, OC*H_B_*CH_2_N, OC*H_C_*C*H_C_*N), 3.50–3.55 (1H, m, OC*H_D_*CH_2_N), 3.61–3.67 (1H, m, OCH_2_C*H_D_*N), 3.84 (3H, s, 3′-OCH_3_), 3.85 (3H, s, 4″-OCH_3_), 3.85 (3H, s, 3″-OCH_3_), 4.88 (1H, dd, *J* = 17.1, 1.9 Hz, 5-H_A_), 4.97 (1H, dd, *J* = 10.3, 1.9 Hz, 5-H_B_), 5.13 (1H, s, 7‴-H), 5.85 (1H, ddd, *J* = 17.1, 10.3, 9.0 Hz, 4-H), 6.59 (1H, dd, *J* = 8.1, 1.9 Hz, 6′-H), 6.65–6.68 (2H, m, 2″-H, 6″-H), 6.69 (1H, d, *J* = 1.9 Hz, 2′-H), 6.74 (1H, d, *J* = 8.1 Hz, 5′-H), 6.77 (1H, d, *J* = 8.5 Hz, 5″-H), 7.25–7.30 (1H, m, 4‴-H), 7.32–7.37 (2H, m, 3‴-H), 7.38–7.42 (2H, m, 2‴-H). δ_C_ (100 MHz; CDCl_3_) 37.4 (C-7′), 38.3 (C-7″), 41.9 (OCH_2_*C*H_CD_N), 46.3 (OCH_2_*C*H_AB_N), 46.5 (C-2), 48.6 (C-3), 56.0, 56.2 (3′, 3″, 4″-OCH_3_), 66.4 (O*C*H_AB_CH_2_N), 66.9 (O*C*H_CD_CH_2_N), 71.2 (C-7‴), 111.1 (C-5″), 112.4 (C-2″), 113.3 (C-2′), 114.6 (C-5′), 116.7 (C-5), 120.9 (C-6′), 121.3 (C-6″), 127.3 (C-2‴), 127.9 (C-4‴), 128.7 (C-3‴), 132.4 (C-1″), 133.1 (C-1′), 137.3 (C-1‴), 139.3 (C-4), 146.7 (C-4′), 147.5 (C-4″), 148.8 (C-3″), 149.7 (C-3′), 172.7 (C-1). IR: ν_MAX_ (film)/cm^−1^; 2936, 1736, 1633, 1513, 1454, 1261, 1140, 1028, 915, 733. HRMS (ESI^+^) Found [M + Na]^+^ 568.2671; C_33_H_39_NNaO_6_ requires 568.2670.

(2R*,3S*)-2-(3′,4′-Dimethoxybenzyl)-3-(3″,4″-methylenedioxybenzyl)-1-morpholinopent-4-en-1-one (**35ba**). Using general procedure A: Morpholine **9b** (0.25 g, 0.96 mmol), acid chloride **34a** (0.26 g, 1.2 mmol) and a reaction time of 21 h. The crude product was purified by column chromatography (1:1 hexanes, ethyl acetate) to give the title compound **35ba** (0.36 g, 83%) as a yellow oil.

R_f_ = 0.50 (1:3 hexanes, ethyl acetate). δ_H_ (400 MHz; CDCl_3_) 2.56 (1H, dd, *J* = 13.4, 9.0 Hz, 7″-H_A_), 2.62–2.70 (1H, m, 3-H), 2.75–2.94 (6H, m, 2-H, 7′-H, 7″-H_B_, NC*H_A_*C*H_A_*O), 3.05 (1H, ddd, *J* = 13.6, 7.9, 3.1 Hz, NC*H_B_*CH_2_O), 3.28–3.41 (3H, m, NCH_2_C*H_B_*O, NC*H_C_*C*H_C_*O), 3.51–3.57 (1H, m, NCH_2_C*H_D_*O), 3.58–3.64 (1H, m, NC*H_D_*CH_2_O), 3.83 (3H, s, 3′-H), 3.84 (3H, s, 4′-H), 4.89 (1H, dd, *J* = 17.2, 1.9 Hz, 5-H_A_), 4.99 (1H, dd, *J* = 10.2, 1.9 Hz, 5-H_B_), 5.86 (1H, ddd, *J* = 17.2, 10.2, 9.1 Hz, 4-H), 5.92 (1H, d, *J* = 1.4 Hz, OCH_A_O), 5.92 (1H, d, *J* = 1.4 Hz, OCH_B_O), 6.58 (1H, dd, *J* = 7.9, 1.6 Hz, 6″-H), 6.64 (1H, d, *J* = 1.6 Hz, 2″-H), 6.66–6.70 (2H, m, 2′, 6′-H), 6.71 (1H, d, *J* = 7.9 Hz, 5″-H), 6.76 (1H, d, *J* = 8.1 Hz, 5′-H). δ_C_ (100 MHz; CDCl_3_) 37.3 (C-7′), 38.5 (C-7″), 42.0 (N*C*H_AB_CH_2_O), 46.3 (N*C*H_CD_CH_2_O), 46.5 (C-2), 48.8 (C-3), 56.1 (3′, 4′-OCH_3_), 66.4 (NCH_2_*C*H_AB_O), 66.9 (NCH_2_*C*H_CD_O), 101.0 (OCH_2_O), 108.1 (C-5″), 109.6 (C-2″), 111.4 (C-5′), 112.7 (C-2′), 116.8 (C-5), 121.0 (C-6′), 122.1 (C-6″), 132.4 (C-1′), 133.7 (C-1″), 139.2 (C-4), 145.9 (C-4″), 147.6 (C-4′), 147.8 (C-3″), 149.0 (C-3′), 172.7 (C-1). IR: ν_MAX_ (film)/cm^−1^; 2908, 1740, 1630, 1515, 1441, 1237, 1029, 923, 730. HRMS (ESI^+^) Found [M + Na]^+^ 476.2042; C_26_H_31_NNaO_6_ requires 476.2044.

(2R*,3S*)-2-(3′,4′-Methylenedioxybenzyl)-3-(3″,4″-methylenedioxybenzyl)-1-morpholinopent-4-en-1-one (**35bb**). Using general procedure A: Morpholine **9b** (0.5 g, 1.91 mmol), acid chloride **34b** (0.49 g, 2.30 mmol) and a reaction time of 30 min. The crude product was purified by column chromatography (1:1 hexanes, ethyl acetate) to give the title compound **35bb** (0.798 g, 95%) as a pale-yellow solid. R_f_ = 0.68 (1:3 hexanes, ethyl acetate). Melting point: 131–133 °C. δ_H_ (400 MHz; CDCl_3_) 2.54 (1H, dd, *J* = 13.5, 8.9 Hz, 7″-H_A_), 2.61–2.69 (1H, m, 3-H), 2.78–2.93 (6H, m, 2-H, 7′-H, 7″-H_B_, NC*H_A_*C*H_A_*O), 3.06 (1H, ddd, *J* = 13.2, 7.8, 3.1 Hz, NC*H_B_*CH_2_O), 3.29–3.41 (3H, m, NCH_2_C*H_B_*O, NC*H_C_*C*H_C_*O), 3.53–3.61 (1H, m, NCH_2_C*H_D_*O), 3.66–3.74 (1H, m, NC*H_D_*CH_2_O), 4.89 (1H, dd, *J* = 17.0, 1.9 Hz, 5-H_A_), 4.99 (1H, dd, *J* = 10.2, 1.9 Hz, 5-H_B_), 5.85 (1H, ddd, *J* = 17.0, 10.2, 9.1 Hz, 4-H), 5.90 (1H, d, *J* = 1.4 Hz, 3′-OCH_A_O), 5.91 (1H, d, *J* = 1.4 Hz, 3′-OCH_B_O), 5.92 (1H, d, *J* = 1.5 Hz, 3″-OCH_A_O), 5.93 (1H, d, *J* = 1.5 Hz, 3″-OCH_B_O), 6.55–6.61 (2H, m, 6′, 6″-H), 6.62–6.64 (2H, m, 2′, 2″-H), 6.70, 6.71 (2 × 1H, 2 × d, *J* = 8.0 Hz, 5′, 5″-H). δ_C_ (100 MHz; CDCl_3_) 37.4 (C-7′), 38.5 (C-7″), 42.0 (N*C*H_CD_CH_2_O), 46.4 (N*C*H_AB_CH_2_O), 46.5 (C-2), 48.8 (C-3), 66.4 (NCH_2_*C*H_AB_O), 67.0 (NCH_2_*C*H_CD_O), 101.0 (2 × OCH_2_O), 108.2, 108.4 (C-5′, 5″), 109.6 (C-2′, 2″), 116.8 (C-5), 122.1 (C-6′, 6″), 133.6 (C-1′, 1″), 139.2 (C-4), 145.9, 146.2 (C-4′, 4″), 147.6, 147.7 (C-3′, 3″), 172.6 (C-1). IR: ν_MAX_ (film)/cm^−1^; 2897, 1630, 1487, 1440, 1244, 1036, 925, 808, 730. HRMS (ESI^+^) Found [M + Na]^+^ 460.1722; C_25_H_27_NNaO_6_ requires 460.1731.

(2R*,3S*)-2-(3′,4′,5′-Trimethoxybenzyl)-3-(3″,4″-methylenedioxybenzyl)-1-morpholinopent-4-en-1-one (**35bc**). Using general procedure A: Morpholine **9b** (0.25 g, 0.96 mmol), acid chloride **34c** (0.27 g, 1.2 mmol) and a reaction time of 18 h. The crude product was purified by column chromatography (1:1 hexanes, ethyl acetate) to give the title compound **35bc** (0.40 g, 86%) as a pale-yellow solid. R_f_ = 0.55 (1:3 hexanes, ethyl acetate). Melting point: 104–106 °C. δ_H_ (400 MHz; CDCl_3_) 2.56 (1H, dd, *J* = 13.4, 9.0 Hz, 7″-H_A_), 2.62–2.70 (1H, m, 3-H), 2.75–2.95 (6H, m, 2-H, 7′-H, 7″-H_B_, NC*H_A_*C*H_A_*O), 3.06 (1H, ddd, *J* = 13.2, 7.7, 3.0 Hz, NC*H_B_*CH_2_O), 3.25–3.40 (3H, m, NCH_2_C*H_B_*O, NC*H_C_*C*H_C_*O), 3.54–3.60 (1H, m, NCH_2_C*H_D_*O), 3.65–6.71 (1H, m, NC*H_D_*CH_2_O), 3.80 (3H, s, 4′-OCH_3_), 3.82 (6H, s, 3′-OCH_3_), 4.90 (1H, dd, *J* = 17.2, 1.9 Hz, 5-H_A_), 5.00 (1H, dd, *J* = 10.2, 1.9 Hz, 5-H_B_), 5.85 (1H, ddd, *J* = 17.2, 10.2, 9.0 Hz, 4-H), 5.92 (1H, d, *J* = 1.4 Hz, OCH_A_O), 5.93 (1H, d, *J* = 1.4 Hz, OCH_B_O), 6.36 (2H, s, 2′-H), 6.59 (1H, dd, *J* = 7.9, 1.6 Hz, 6″-H), 6.65 (1H, d, *J* = 1.6 Hz, 2″-H), 6.72 (1H, d, *J* = 7.9 Hz, 5″-H). δ_C_ (100 MHz; CDCl_3_) 38.1 (C-7′), 38.5 (C-7″), 42.0 (N*C*H_CD_CH_2_O), 46.4 (C-2, N*C*H_AB_CH_2_O), 48.9 (C-3), 56.4 (3′-OCH_3_), 61.1 (4′-OCH_3_), 66.4 (NCH_2_*C*H_AB_O), 67.0 (NCH_2_*C*H_CD_O), 101.0 (OCH_2_O), 106.2 (C-2′), 108.2 (C-5″), 109.6 (C-2″), 116.9 9 (C-5), 122.1 (C-6″), 133.6 (C-1″), 135.6 (C-1′), 136.9 (C-4′), 139.1 (C-4), 145.9 (C-4″), 147.7 (C-3″), 153.3 (C-3′), 172.6 (C-1). IR: ν_MAX_ (film)/cm^−1^; 2922, 1632, 1589, 1490, 1240, 1120, 1036, 925, 730. HRMS (ESI^+^) Found [M + Na]^+^ 506.2145; C_27_H_33_NNaO_7_ requires 506.2149.

(2R*,3S*)-2-(3′-Methoxy-4′-benzyloxybenzyl)-3-(3″,4″-methylenedioxybenzyl)-1-morpholinopent-4-en-1-one (**35bd**). Using general procedure A: Morpholine **9b** (0.25 g, 0.96 mmol), acid chloride **34d** (0.35 g, 1.2 mmol) and a reaction time of 18 h. The crude product was purified by column chromatography (1:1 hexanes, ethyl acetate) to give the title compound **35bd** (0.45 g, 88%) as a yellow oil.

R_f_ = 0.67 (1:3 hexanes, ethyl acetate). δ_H_ (400 MHz; CDCl_3_) 2.54 (1H, dd, *J* = 13.5, 8.9 Hz, 7″-H_A_), 2.61–2.70 (2H, m, 3-H, NCH_2_C*H_A_*O), 2.73–2.91 (5H, m, 2-H, 7′-H, 7″-H_B_, NC*H_A_*CH_2_O), 2.99 (1H, ddd, *J* = 13.2, 7.7, 3.0 Hz, NC*H_B_*CH_2_O), 3.20–3.35 (3H, m, NCH_2_C*H*_B_O, NC*H_C_*C*H_C_*O), 3.53 (1H, ddd, *J* = 11.0, 5.5, 2.5 Hz, NCH_2_C*H_D_*O), 3.62 (1H, ddd, *J* = 13.0, 5.5, 2.5 Hz, NC*H_D_*CH_2_O), 3.84 (3H, s, 3′-OCH_3_), 4.88 (1H, dd, *J* = 17.0, 1.9 Hz, 5-H_A_), 4.98 (1H, dd, *J* = 10.2, 1.9 Hz, 5-H_B_), 5.13 (2H, s, 7‴-H), 5.84 (1H, ddd, *J* = 17.0, 10.2, 9.1 Hz, 4-H), 5.91 (1H, d, *J* = 1.4 Hz, OCH_A_O), 5.92 (1H, d, *J* = 1.4 Hz, OCH_B_O), 6.57 (1H, dd, *J* = 8.0, 1.9 Hz, 6″-H), 6.59 (1H, dd, *J* = 8.2, 1.8 Hz, 6′-H), 6.64 (1H, d, *J* = 1.8 Hz, 2″-H), 6.69 (1H, d, *J* = 1.9 Hz, 2″-H), 6.71 (1H, d, *J* = 8.0 Hz, 5″-H), 6.75 (1H, d, *J* = 8.2 Hz, 5′-H), 7.25–7.30 (1H, m, 4‴-H), 7.32–7.37 (2H, m, 3‴-H), 7.38–7.43 (2H, m, 2‴-H). δ_C_ (100 MHz; CDCl_3_) 37.4 (C-7′), 38.5 (C-7″), 41.9 (N*C*H_CD_CH_2_O), 46.3 (N*C*H_AB_CH_2_O), 46.4 (C-2), 48.8 (C-3), 56.2 (3′-OCH_3_), 66.3 (NCH_2_*C*H_AB_O), 66.9 (NCH_2_*C*H_CD_O), 71.2 (C-7‴), 100.9 (OCH_2_O), 108.1 (C-5″), 109.6 (C-2″), 113.2 (C-2′), 114.5 (C-5′), 116.7 (C-5), 121.0 (C-6′), 122.1 (C-6″), 127.3 (C-2‴), 127.9 (C-4‴), 128.6 (C-3‴), 133.1 (C-1′) 133.6 (C-1″), 137.3 (C-1‴), 139.2 (C-4), 145.9 (C-4″), 146.7 (C-4′), 147.6 (C-3″), 149.7 (C-3′), 172.6 (C-1). IR: ν_MAX_ (film)/cm^−1^; 2920, 1630, 1489, 1231, 1114, 1034, 913, 729. HRMS (ESI^+^) Found [M + Na]^+^ 552.2354; C_32_H_35_NNaO_6_ requires 552.2357.

(3R*,4R*)-3-(3′,4′-Methylenedioxybenzyl)-4-(3″,4″-dimethoxybenzyl)-5-(hydroxymethyl)dihydrofuran-2(3H)-one (**4ab**). Using general procedure B: Amide **35ab** (0.38 g, 0.84 mmol) in *^t^*BuOH/H_2_O and a reaction time of 3 days. The crude product was purified by column chromatography (1:1 hexanes, ethyl acetate) to give the title compound **4ab** (180 mg, 54%) as a white foam. R_f_ = 0.50 (19:1 CH_2_Cl_2_, methanol). δ_H_ (400 MHz; CDCl_3_) 1.79 (1H, t, *J* = 6.4 Hz, 6-OH), 2.36–2.44 (1H, m, 4-H), 2.51 (1H, dd, *J* = 13.7, 7.9 Hz, 7″-H_A_), 2.58 (1H, dd, *J* = 13.7, 6.6 Hz, 7″-H_B_), 2.68 (1H, ddd, *J* = 9.3, 7.0, 5.5 Hz, 3-H), 2.85 (1H, dd, *J* = 14.0, 7.0 Hz, 7′-H_A_), 2.92 (1H, dd, *J* = 14.0, 5.5 Hz, 7′-H_B_), 3.15 (1H, ddd, *J* = 12.5, 6.4, 5.1 Hz, 6-H_A_), 3.54 (1H, ddd, *J* = 12.5, 6.4, 2.5 Hz, 6-H_B_), 3.83 (3H, s, 3″-OCH_3_), 3.85 (3H, s, 4″-OCH_3_), 4.19 (1H, ddd, *J* = 8.0, 5.1, 2.5 Hz, 5-H), 5.92 (1H, d, *J* = 1.5 Hz, OCH*_A_*H_B_O), 5.93 (1H, d, *J* = 1.5 Hz, OCH_A_*H_B_*O), 6.47 (1H, d, *J* = 2.0 Hz, 2″-H), 6.57–6.60 (2H, m, 6′ and 6″-H), 6.61 (1H, d, *J* = 1.5 Hz, 2′-H), 6.71 (1H, d, *J* = 7.8 Hz, 5′-H), 6.77 (1H, d, *J* = 8.1 Hz, 5″-H). δ_C_ (100 MHz; CDCl_3_) 35.3 (C-7′), 38.7 (C-7″), 41.6 (C-4), 47.6 (C-3), 56.0 (3″-OCH_3_, 4″-OCH_3_), 63.2 (C-6), 84.1 (C-5), 101.2 (OCH_2_O), 108.3 (C-5′), 109.7 (C-2′), 111.4 (C-5″), 112.0 (C-2″), 121.0 (C-6″), 122.5 (C-6′), 130.3 (C-1″), 131.6 (C-1′), 146.6 (C-4′), 148.0 (C-4″), 148.1 (C-3′), 149.3 (C-3″), 177.7 (C-2). IR: ν_MAX_ (film)/cm^−1^; 3496 (broad), 2936, 2254, 1760, 1515, 1489, 1442, 1239, 1025, 909, 809, 766. HRMS (ESI^+^) Found [M + Na]^+^ 423.1427; C_22_H_24_NaO_7_ requires 423.1414.

(3R*,4R*)-3,4-bis(3′,4′-Dimethoxybenzyl)-5-(hydroxymethyl)dihydrofuran-2(3H)-one (**4aa**). Using general procedure B: Amide **35aa** (0.29 g, 0.61 mmol), in *^t^*BuOH/H_2_O and a reaction time of 6 days. The crude product was purified by flash chromatography (1:1 hexanes, ethyl acetate) to give the title compound **4aa** (0.18 g, 70%) as a colourless oil. R_f_ = 0.32 (19:1 CH_2_Cl_2_, methanol). δ_H_ (400 MHz; CDCl_3_) 2.39–2.44 (1H, m, 4-H), 2.53 (1H, dd, *J* = 13.7, 7.3 Hz, 7″-H_A_), 2.58 (1H, dd, *J* = 13.7, 6.5 Hz, 7″-H_B_), 2.64 (1H, br s, 6-OH), 2.71 (1H, ddd, *J* = 9.3, 6.7, 5.7 Hz, 3-H), 2.88 (1H, dd, *J* = 14.0, 6.7 Hz, 7′-H_A_), 2.94 (1H, dd, *J* = 14.0, 5.5 Hz, 7′-H_B_), 3.16 (1H, dd, *J* = 12.6, 4.9 Hz, 6-H_A_), 3.53 (1H, dd, *J* = 12.6, 2.4 Hz, 6-H_B_), 3.81, 3.83, 3.84 (12H, s, 3′, 4′, 3″, 4″-OCH_3_), 4.15 (1H, ddd, *J* = 8.0, 4.9, 2.4 Hz, 5-H), 6.49 (1H, d, *J* = 1.9 Hz, 2″-H), 6.57 (1H, dd, *J* = 8.1, 1.9 Hz, 6″-H), 6.66–6.68 (2H, m, 2′, 6′-H), 6.73–6.80 (2H, m, 5′, 5″-H). δ_C_ (100 MHz; CDCl_3_) 35.0 (C-7′), 38.5 (C-7″), 41.6 (C-4), 47.5 (C-3), 55.8 (3′, 4′, 3″, 4″-OCH_3_), 62.9 (C-6), 84.0 (C-5), 111.2, 111.4 (C-5′, 5″), 112.1 (C-2″), 112.6 (C-2′), 120.9 (C-6″), 121.4 (C-6′), 130.4 (C-1′, 1″), 147.9 (C-4′, 4″), 149.0 (C-3′, 3″), 178.0 (C-2). IR: ν_MAX_ (film)/cm^−1^; 3505 (br), 2938, 1761, 1591, 1514, 1465, 1259, 1156, 1025, 910, 808, 766, 647. HRMS (ESI^+^) Found [M + H]^+^ 417.1909; C_23_H_29_O_7_ requires 417.1908.

(3R*,4R*)-3-(3′,4′,5′-Trimethoxybenzyl)-4-(3″,4″-dimethoxybenzyl)-5-(hydroxymethyl)dihydrofuran-2(3H)-one (**4ac**). Using general procedure B: Amide **35ac** (0.45 g, 0.90 mmol) in *^t^*BuOH/H_2_O/THF and a reaction time of 3 days. The crude product was purified by column chromatography (1:1 hexanes, ethyl acetate) to give the title compound **4ac** (0.17 g, 42%) as a pale-yellow solid.

R_f_ = 0.31 (19:1 CH_2_Cl_2_, methanol). Melting point: 141–142 °C. δ_H_ (400 MHz; CDCl_3_) 1.68 (1H, t, *J* = 6.5 Hz, 6-OH), 2.38–2.46 (1H, m, 4-H), 2.55 (1H, dd, *J* = 13.8, 8.2 Hz, 7″-H_A_), 2.65 (1H, dd, *J* = 13.8, 5.9 Hz, 7″-H_B_), 2.72 (1H, ddd, *J* = 9.7, 6.3, 5.7 Hz, 3-H), 2.90 (1H, dd, *J* = 14.0, 6.3 Hz, 7′-H_A_), 2.95 (1H, dd, *J* = 14.0, 5.7 Hz, 7′-H_B_), 3.15 (1H, ddd, *J* = 12.4, 5.1, 5.4 Hz, 6-H_A_), 3.54 (1H, ddd, *J* = 12.4, 6.5, 2.5 Hz, 6-H_B_), 3.82 (6H, s, 4′, 3″-OCH_3_), 3.83 (6H, s, 3′-OCH_3_), 3.85 (3H, s, 4″-OCH_3_), 4.20 (1H, ddd, *J* = 8.2, 5.1, 2.5 Hz, 5-H), 6.38 (2H, s, 2′-H), 6.49 (1H, d, *J* = 2.0 Hz, 2″-H), 6.58 (1H, dd, *J* = 8.1, 2.0 Hz, 6″-H), 6.76 (1H, d, *J* = 8.1 Hz, 5″-H). δ_C_ (100 MHz; CDCl_3_) 35.7 (C-7′), 38.6 (C-7″), 41.8 (C-4), 47.7 (C-3), 56.0, 56.1 (3″, 4″-OCH_3_), 56.3 (3′-OCH_3_), 61.0 (4′-OCH_3_), 63.2 (C-6), 83.9 (C-5), 106.5 (C-2′), 111.5 (C-5″), 112.2 (C-2″), 121.0 (C-6″), 130.3 (C-1″), 133.7 (C-1′), 137.2 (C-4′), 148.2 (C-4″), 149.3 (C-3″), 153.5 (C-3′), 177.7 (C-2). IR: ν_MAX_ (film)/cm^−1^; 3527 (br), 2938, 1761, 1590, 1514, 1237, 1126, 1026, 735. HRMS (ESI^+^) Found [M + Na]^+^ 469.1839; C_24_H_30_NaO_8_ requires 469.1833.

(3R*,4R*)-3-(3′-Methoxy-4′-benzyloxybenzyl)-4-(3″,4″-dimethoxybenzyl)-5-(hydroxymethyl)dihydrofuran-2(3H)-one (**4ad**). Using general procedure B: Amide **35ad** (0.59 g, 1.1 mmol) in *^t^*BuOH/H_2_O and a reaction time of 7 days. The crude product was purified by column chromatography (1:1 hexanes, ethyl acetate) to give the title compound **4ad** (0.30 g, 56%) as a cloudy oil. R_f_ = 0.27 (19:1 CH_2_Cl_2_, methanol). δ_H_ (400 MHz; CDCl_3_) 1.57 (1H, t, *J* = 6.5 Hz, 6-OH), 2.34–2.42 (1H, m, 4-H), 2.50 (1H, dd, *J* = 13.5, 8.0 Hz, 7″-H_A_), 2.59 (1H, dd, *J* = 13.5, 6.0 Hz, 7″-H_B_), 2.70 (1H, ddd, *J* = 9.7, 6.2, 5.6 Hz, 3-H), 2.90 (1H, dd, *J* = 14.1, 6.2 Hz, 7′-H_A_), 2.94 (1H, dd, *J* = 14.1, 5.6 Hz, 7′-H_B_), 3.10 (1H, ddd, *J* = 12.5, 6.5, 5.2 Hz, 6-H_A_), 3.48 (1H, ddd, *J* = 12.5, 6.5, 2.7 Hz, 6-H_B_), 3.80 (3H, s, 3″-OCH_3_), 3.86 (6H, s, 3′, 4″-OCH_3_), 4.18 (1H, ddd, *J* = 8.3, 5.2, 2.7 Hz, 5-H), 5.12 (2H, s, 7‴-H), 6.46 (1H, d, *J* = 2.0 Hz, 2″-H), 6.56 (1H, dd, *J* = 8.0, 2.0 Hz, 6″-H), 6.61 (1H, dd, *J* = 8.1, 2.0 Hz, 6′-H), 6.72 (1H, d, *J* = 2.0 Hz, 2′-H), 6.75 (1H, d, *J* = 8.0 Hz, 5″-H), 6.79 (1H, d, *J* = 8.1 Hz, 5′-H), 7.25–7.30 (1H, m, 4‴-H), 7.31–7.36 (2H, m, 3‴-H), 7.39–7.42 (2H, m, 2‴-H). δ_C_ (100 MHz; CDCl_3_) 35.1 (C-7′), 38.6 (C-7″), 41.7 (C-4), 47.6 (C-3), 56.0 (3″, 4″-OCH_3_), 56.2 (3′-OCH_3_), 63.3 (C-6), 71.3 (C-7‴), 84.0 (C-5), 111.5 (C-5″), 112.1 (C-2″), 113.3 (C-2′), 114.3 (C-5′), 121.0 (C-6″), 121.6 (C-6′), 127.4 (C-2‴), 128.0 (C-4‴), 128.7 (C-3‴), 130.3 (C-1″), 131.1 (C-1′), 137.2 (C-1‴), 147.2 (C-4′), 148.2 (C-4″), 149.3 (C-3″), 150.0 (C-3′), 177.8 (C-2). IR: ν_MAX_ (film)/cm^−1^; 3523 (br), 2935, 1761, 1514, 1261, 1025, 911, 730. HRMS (ESI^+^) Found [M + Na]^+^ 515.2023; C_29_H_32_NaO_7_ requires 515.2040.

(3R*,4R*,5S*)-4-(3″,4″-Dimethoxybenzyl)-3-(4′-hydroxy-3′-methoxybenzyl)-5-(hydroxymethyl)dihydrofuran-2(3H)-one (**4ae**). Using general procedure F: Benzyl ether **4ad** (0.27 g, 0.55 mmol) gave the title compound **4ae** (0.19 g, 88%) as a yellow solid. R_f_ = 0.43 (19:1 CH_2_Cl_2_, methanol). Melting point: 183–185 °C. δ_H_ (400 MHz; CDCl_3_) 1.63 (1H, t, *J* = 6.5 Hz, 6-OH), 2.34–2.43 (1H, m, 4-H), 2.53 (1H, dd, *J* = 13.8, 8.1 Hz, 7″-H_A_), 2.62 (1H, dd, *J* = 13.8, 6.1 Hz, 7″-H_B_), 2.69 (1H, dt, *J* = 9.5, 6.0 Hz, 3-H), 2.92 (2H, d, *J* = 6.0 Hz, 7′-H), 3.13 (1H, ddd, *J* = 12.5, 6.5, 5.3 Hz, 6-H_A_), 3.51 (1H, ddd, *J* = 12.5, 6.5, 2.5 Hz, 6-H_B_), 3.82 (3H, s, 3′-OCH_3_), 3.84 (3H, s, 3″-OCH_3_), 3.85 (3H, s, 4″-OCH_3_), 4.19 (1H, ddd, *J* = 8.0, 5.3, 2.5 Hz, 5-H), 5.52 (1H, s, 4′-OH), 6.46 (1H, d, *J* = 2.0 Hz, 2″-H), 6.57 (1H, dd, *J* = 8.1, 2.0 Hz, 6″-H), 6.63 (1H, dd, *J* = 8.0, 1.9 Hz, 6′-H), 6.66 (1H, d, *J* = 1.9 Hz, 2′-H), 6.76 (1H, d, *J* = 8.1 Hz, 5″-H), 6.83 (1H, d, *J* = 8.0 Hz, 5′-H). δ_C_ (100 MHz; CDCl_3_) 35.1 (C-7′), 38.6 (C-7″), 41.6 (C-4), 47.7 (C-3), 56.0, 56.1 (3′, 3″, 4″-OCH_3_), 63.4 (C-6), 84.1 (C-5), 111.5 (C-5″), 111.9 (C-2′), 112.1 (C-2″), 114.4 (C-5′), 121.0 (C-6″), 122.3 (C-6′), 129.7 (C-1′), 130.4 (C-1″), 144.7 (C-4′), 146.8 (C-3′), 148.2 (C-4″), 149.3 (C-3″), 177.8 (C-2). IR: ν_MAX_ (film)/cm^−1^; 3438 (br), 2937, 1755, 1514, 1236, 1155, 1025, 907, 723. HRMS (ESI^+^) Found [M + Na]^+^ 425.1564; C_22_H_26_NaO_7_ requires 425.1571.

(3R*,4R*)-3,4-bis(3′,4′-Methylenedioxybenzyl)-5-(hydroxymethyl)dihydrofuran-2(3H)-one (**4bb**). Using general procedure B: Morpholine amide **35bb** (0.322 g, 0.74 mmol) in *^t^*BuOH/H_2_O/THF and a reaction time of 5 days. The crude product was then purified by column chromatography (2:1 hexanes, ethyl acetate) to give the title compound **4bb** (0.145 g, 51%) as a pale-yellow oil.

R_f_ = 0.59 (1:3 hexanes, ethyl acetate). δ_H_ (400 MHz; CDCl_3_) 1.72 (1H, br, 6-OH), 2.32–2.41 (1H, m, 4-H), 2.47 (1H, dd, *J* = 13.7, 8.1 Hz, 7″-H_A_), 2.56 (1H, dd, *J* = 13.7, 6.2 Hz, 7″-H_B_), 2.65 (1H, ddd, *J* = 9.0, 7.5, 5.3 Hz, 3-H), 2.85 (1H, dd, *J* = 14.0, 7.5 Hz, 7′-H_A_), 2.96 (1H, dd, *J* = 14.0, 5.3 Hz, 7′-H_B_), 3.15 (1H, dd, *J* = 12.6, 4.9 Hz, 6-H_A_), 3.54 (1H, dd, *J* = 12.6, 2.5 Hz, 6-H_B_), 4.18 (1H, ddd, *J* = 7.7, 4.9, 2.5 Hz, 5-H), 5.93–5.95 (4H, m, 2 × OCH_2_O), 6.45–6.49 (2H, m, 2″, 6″-H), 6.60 (1H, dd, *J* = 7.8, 1.7 Hz, 6′-H), 6.63 (1H, d, *J* = 1.7 Hz, 2′-H), 6.70 (1H, d, *J* = 7.8 Hz, 5″-H), 6.73 (1H, d, *J* = 7.8 Hz, 5′-H). δ_C_ (100 MHz; CDCl_3_) 35.4 (C-7′), 38.9 (C-7″), 41.8 (C-4), 47.6 (C-3), 63.3 (C-6), 83.9 (C-5), 101.1, 101.2 (2 × OCH_2_O), 108.4 (C-5′), 108.6 (C-5″), 109.2 (C-2″), 109.6 (C-2′), 121.9 (C-6″), 122.4 (C-6′), 131.5 (C-1′, 1″), 146.6 (C-4′, 4″), 148.0, 148.1 (C-3′, 3″), 177.6 (C-2). IR: ν_MAX_ (film)/cm^−1^; 3432 (br), 2922, 1760, 1503, 1490, 1444, 1247, 1038, 927, 811. HRMS (ESI^+^) Found [M + H]^+^ 385.1279; C_21_H_21_O_7_ requires 385.1282.

(3R*,4R*)-3-(3′,4′-Dimethoxybenzyl)-4-(3″,4″-methylenedioxybenzyl)-5-(hydroxymethyl)dihydrofuran-2(3H)-one (**4ba**). Using general procedure B: Morpholine amide **35ba** (0.336 g, 0.74 mmol) in *^t^*BuOH/H_2_O/THF and a reaction time of 4 days. The crude product was then purified by column chromatography (1:3 hexanes, ethyl acetate) to give the title compound **4ba** (0.103 g, 34%) as a pale yellow oil.

R_f_ = 0.48 (1:3 hexanes, ethyl acetate). δ_H_ (400 MHz; CDCl_3_) 1.68 (1H, t, *J* = 6.6 Hz, 6-OH), 2.33–2.42 (1H, m, 4-H), 2.48 (1H, dd, *J* = 13.7, 7.9 Hz, 7″-H_A_), 2.56 (1H, dd, *J* = 13.7, 6.3 Hz, 7″-H_B_), 2.68 (1H, ddd, *J* = 9.3, 6.9, 5.4 Hz, 3-H), 2.89 (1H, dd, *J* = 14.0, 6.9 Hz, 7′-H_A_), 2.96 (1H, dd, *J* = 14.0, 5.4 Hz, 7′-H_B_), 3.15 (1H, ddd, *J* = 12.5, 6.6, 5.2 Hz, 6-H_A_), 3.52 (1H, ddd, *J* = 12.5, 6.6, 2.6 Hz, 6-H_B_), 3.85 (3H, s, 3′-OCH_3_), 3.86 (3H, s, 4′-OCH_3_), 4.18 (1H, ddd, *J* = 7.9, 5.2, 2.6 Hz, 5-H), 5.93 (1H, d, *J* = 1.4 Hz, OCH_A_O), 5.94 (1H, d, *J* = 1.4 Hz, OCH_B_O), 6.44 (1H, d, *J* = 1.6 Hz, 2″-H), 6.47 (1H, dd, *J* = 7.8, 1.6 Hz, 6″-H), 6.67 (1H, d, *J* = 2.2 Hz, 2′-H), 6.68–6.72 (2H, m, 6′, 5″-H), 6.79 (1H, d, *J* = 8.0 Hz, 5′-H). δ_C_ (100 MHz; CDCl_3_) 35.2 (C-7′), 38.8 (C-7″), 41.7 (C-4), 47.6 (C-3), 56.0 (3′, 4′-OCH_3_), 63.4 (C-6), 83.8 (C-5), 101.3 (OCH_2_O), 108.5 (C-5′), 109.2 (C-2″), 111.3 (C-5″), 112.5 (C-2′), 121.6 (C-6′), 121.9 (C-6″), 130.3 (C-1′), 131.5 (C-1″), 146.7 (C-4″), 148.1 (C-4′, 3″), 149.2 (C-3′), 177.7 (C-2). IR: ν_MAX_ (film)/cm^−1^; 3472 (br), 2933, 1760, 1516, 1490, 1242, 1157, 1028, 925, 810, 730. HRMS (ESI^+^) Found [M + Na]^+^ 423.1423; C_22_H_24_NaO_7_ requires 423.1414.

(3R*,4R*)-3-(3′,4′,5′-Trimethoxybenzyl)-4-(3″,4″-methylenedioxybenzyl)-5-(hydroxymethyl)dihydrofuran-2(3H)-one (**4bc**). Using general procedure B: Morpholine amide **35bc** (0.372 g, 0.77 mmol) in *^t^*BuOH/H_2_O/THF and a reaction time of 4 days. The crude product was then purified by column chromatography (1:3 hexanes, ethyl acetate) to give the title compound **4bc** (0.084 g, 25%) as a pale-yellow oil.

R_f_ = 0.38 (1:3 hexanes, ethyl acetate). δ_H_ (400 MHz; CDCl_3_) 1.69 (1H, t, *J* = 6.6 Hz, 6-OH), 2.36–2.45 (1H, m, 4-H), 2.53 (1H, dd, *J* = 13.8, 7.6 Hz, 7″-H_A_), 2.59 (1H, dd, *J* = 13.8, 6.8 Hz, 7″-H_B_), 2.70 (1H, ddd, *J* = 9.5, 6.7, 5.4 Hz, 3-H), 2.87 (1H, dd, *J* = 14.0, 6.7 Hz, 7′-H_A_), 2.93 (1H, dd, *J* = 14.0, 5.4 Hz, 7′-H_B_), 3.22 (1H, ddd, *J* = 12.7, 6.6, 5.0 Hz, 6-H_A_), 3.58 (1H, ddd, *J* = 12.7, 6.6, 2.5 Hz, 6-H_B_), 3.82 (3H, s, 4′-OCH_3_), 3.84 (6H, s, 3′-OCH_3_), 3.85 (3H, s, 4″-OCH_3_), 4.19 (1H, ddd, *J* = 7.9, 5.0, 2.5 Hz, 5-H), 5.94 (1H, d, *J* = 1.4 Hz, OCH_A_O), 5.94 (1H, d, *J* = 1.4 Hz, OCH_B_O), 6.37 (2H, s, 2′-H), 6.46 (1H, d, *J* = 1.8 Hz, 2″-H), 6.48 (1H, dd, *J* = 7.9, 1.8 Hz, 6″-H), 6.70 (1H, d, *J* = 7.9 Hz, 5″-H). δ_C_ (100 MHz; CDCl_3_) 36.0 (C-7′), 38.8 (C-7″), 41.9 (C-4), 47.6 (C-3), 56.3 (3′-OCH_3_), 61.1 (4′-OCH_3_), 63.3 (C-6), 83.8 (C-5), 101.3 (OCH_2_O), 106.5 (C-2′), 108.5 (C-5″), 109.2 (C-2″), 121.9 (C-6″), 131.4 (C-1″), 133.6 (C-1′), 137.1 (C-4′), 146.7 (C-4″), 148.2 (C-3″), 153.5 (C-3′), 177.7 (C-2). IR: ν_MAX_ (film)/cm^−1^; 3475 (br), 2941, 1760, 1591, 1490, 1445, 1244, 1127, 1036, 926. HRMS (ESI^+^) Found [M + Na]^+^ 453.1519; C_23_H_26_NaO_8_ requires 453.1520.

(3R*,4R*)-3-(3′-Methoxy-4′-benzyloxybenzyl)-4-(3″,4″-methylenedioxybenzyl)-5-(hydroxymethyl)dihydrofuran-2(3H)-one (**4bd**). Using general procedure B: Morpholine amide **35bd** (0.405 g, 0.77 mmol) in *^t^*BuOH/H_2_O/THF and a reaction time of 5 days. The crude product was then purified by column chromatography (1:3 hexanes, ethyl acetate) to give the title compound **4bd** (0.205 g, 56%) as a pale-yellow oil.

R_f_ = 0.58 (1:3 hexanes, ethyl acetate). δ_H_ (400 MHz; CDCl_3_) 1.64 (1H, t, *J* = 6.6 Hz, 6-OH), 2.31–2.40 (1H, m, 4-H), 2.46 (1H, dd, *J* = 13.7, 7.9 Hz, 7″-H_A_), 2.53 (1H, dd, *J* = 13.7, 6.3 Hz, 7″-H_B_), 2.67 (1H, ddd, *J* = 9.2, 7.2, 5.3 Hz, 3-H), 2.87 (1H, dd, *J* = 14.0, 7.2 Hz, 7′-H_A_), 2.95 (1H, dd, *J* = 14.0, 5.3 Hz, 7′-H_B_), 3.13 (1H, ddd, *J* = 12.6, 6.6, 5.1 Hz, 6-H_A_), 3.48 (1H, ddd, *J* = 12.6, 6.6, 2.6 Hz, 6-H_B_), 3.86 (3H, s, 3′-OCH_3_), 4.17 (1H, ddd, *J* = 7.8, 5.1, 2.6 Hz, 5-H), 5.13 (2H, s, 7‴-H), 5.93 (1H, d, *J* = 1.4 Hz, OCH_A_O), 5.94 (1H, d, *J* = 1.4 Hz, OCH_B_O), 6.43 (1H, d, *J* = 1.6 Hz, 2″-H), 6.45 (1H, dd, *J* = 7.9, 1.6 Hz, 6″-H), 6.64 (1H, dd, *J* = 8.2, 2.0 Hz, 6′-H), 6.68–6.70 (2H, m, 2′, 5″-H), 6.81 (1H, d, *J* = 8.2 Hz, 5′-H), 7.27–7.30 (1H, m, 4‴-H), 7.32–7.36 (2H, m, 3‴-H), 7.40–7.44 (2H, m, 2‴-H). δ_C_ (100 MHz; CDCl_3_) 35.3 (C-7′), 38.8 (C-7″), 41.8 (C-4), 47.6 (C-3), 56.1 (3′-OCH_3_), 63.4 (C-6), 71.3 (C-7‴), 83.8 (C-5), 101.3 (OCH_2_O), 108.5 (C-5″), 109.2 (C-2″), 113.0 (C-2′), 114.4 (C-5′), 121.5 (C-6′), 121.9 (C-6″), 127.5 (C-2‴), 128.0 (C-4‴), 128.7 (C-3‴), 131.0 (C-1′), 131.5 (C-1″), 137.3 (C-1‴), 146.7 (C-4″), 147.2 (C-4′), 148.1 (C-3″), 150.0 (C-3′), 177.7 (C-2). IR: ν_MAX_ (film)/cm^−1^; 3471 (br), 2940, 1743, 1504, 1490, 1366, 1230, 1036, 926, 735. HRMS (ESI^+^) Found [M + Na]^+^ 499.1729; C_28_H_28_NaO_7_ requires 499.1727.

(3R*,4R*,5S*)-4-(3″,4″-Methylenedioxybenzyl)-3-(4′-hydroxy-3′-methoxybenzyl)-5-(hydroxymethyl)dihydrofuran-2(3H)-one (**4be**). Using general procedure F: Benzyl ether **4bd** (0.02 g, 0.04 mmol) and a reaction time of 1 h. The crude product was then purified by column chromatography (1:3 hexanes, ethyl acetate) to give the title compound **4be** (0.017 g, quant.) as a colourless oil. R_f_ = 0.52 (1:3 hexanes, ethyl acetate). δ_H_ (400 MHz; CDCl_3_) 1.74 (1H, br, 6-OH), 2.33–2.42 (1H, m, 4-H), 2.48 (1H, dd, *J* = 13.7, 8.0 Hz, 7″-H_A_), 2.57 (1H, dd, *J* = 13.7, 6.2 Hz, 7″-H_B_), 2.67 (1H, ddd, *J* = 9.4, 6.9, 5.5 Hz, 3-H), 2.88 (1H, dd, *J* = 14.0, 6.9 Hz, 7′-H_A_), 2.94 (1H, dd, *J* = 14.0, 5.5 Hz, 7′-H_B_), 3.15 (1H, br d, *J* = 12.6 Hz, 6-H_A_), 3.52 (1H, br d, *J* = 12.6 Hz, 6-H_B_), 3.86 (3H, s, 3′-OCH_3_), 4.18 (1H, ddd, *J* = 8.0, 5.0, 2.5 Hz, 5-H), 5.54 (1H, s, 4′-OH), 5.93 (1H, d, *J* = 1.4 Hz, OCH_A_O), 5.94 (1H, d, *J* = 1.4 Hz, OCH_B_O), 6.45 (1H, d, *J* = 1.9 Hz, 2″-H), 6.47 (1H, dd, *J* = 7.7, 1.9 Hz, 6″-H), 6.63 (1H, dd, *J* = 8.0, 1.9 Hz, 6′-H), 6.67 (1H, d, *J* = 1.9 Hz, 2′-H), 6.70 (1H, d, *J* = 7.7 Hz, 5″-H), 6.84 (1H, d, *J* = 8.0 Hz, 5′-H). δ_C_ (100 MHz; CDCl_3_) 35.3 (C-7′), 38.8 (C-7″), 41.7 (C-4), 47.7 (C-3), 56.1 (3′-OCH_3_), 63.4 (C-6), 83.9 (C-5), 101.3 (OCH_2_O), 108.6 (C-5″), 109.2 (C-2″), 111.8 (C-2′), 114.5 (C-5′), 121.9 (C-6″), 122.3 (C-6′), 129.6 (C-1′), 131.5 (C-1″), 144.7 (C-4′), 146.7 (C-3′), 146.8 (C-4″), 148.1 (C-3″), 177.8 (C-2). IR: ν_MAX_ (film)/cm ^−1^; 3449 (br), 2933, 1754, 1516, 1490, 1246, 1036, 926, 812. HRMS (ESI^+^) Found [M + Na]^+^ 409.1246; C_21_H_22_NaO_7_ requires 409.1258.

*(±)-Arcitin* (**1aa**). Using general procedure C: Lactone **4aa** (0.16 g, 0.39 mmol) and a reaction time of 2 h to give triol **38aa** (0.17 g, quant.) as a colourless oil. Then using general procedure D: Triol **38aa** (0.16 g, 0.37 mmol) and a reaction time of 2.5 h to give lactol **39aa** (0.14 g, 97%) which was used without further purification. Then using general procedure E: Lactol **39aa** (0.054 g, 0.14 mmol) and a reaction time of 3 h. The crude product was purified by column chromatography (1:1, hexanes, ethyl acetate) to give the title compound **1aa** (0.05 g, 88%) as a pale yellow amorphous solid. R_f_ = 0.45 (19:1, CH_2_Cl_2_, methanol). Melting point: 114–116 °C [lit. [49] 113 °C]. δ_H_ (400 MHz; CDCl_3_) 2.45–2.68 (4H, m, 8, 7′, 8′-H), 2.92 (1H, dd, *J* = 14.3, 6.8 Hz, 7-H_A_), 2.97 (1H, dd, *J* = 14.3, 5.5 Hz, 7-H_B_), 3.82 (3H, s, 3′-OCH_3_), 3.83 (3H, s, 3-OCH_3_), 3.85–3.90 (7H, m, 4, 4′-OCH_3_, 9′-H_A_), 4.13 (1H, t, *J* = 7.0 Hz, 9′-H_B_), 6.49 (1H, d, *J* = 1.9 Hz, 2′-H), 6.55 (1H, dd, *J* = 8.1, 1.9 Hz, 6′-H), 6.66 (1H, dd, *J* = 8.1, 1.9 Hz, 6-H), 6.69 (1H, d, *J* = 1.9 Hz, 2-H), 6.75 (1H, d, *J* = 8.1 Hz, 5-H), 6.77 (1H, d, *J* = 8.1 Hz, 5′-H). δ_C_ (100 MHz; CDCl_3_) 34.5 (C-7), 38.2 (C-7′), 41.1 (C-8′), 46.6 (C-8), 55.8, 55.9 (3, 4, 3′, 4′-OCH_3_), 71.2 (C-9′), 111.1 (C-5), 111.4 (C-5′), 111.9 (C-2′), 112.4 (C-2), 120.6 (C-6′), 121.4 (C-6), 130.2 (C-1), 130.5 (C-1′), 147.9 (C-4′), 148.0 (C-4), 149.1 (C-3, 3′), 178.7 (C-9). IR: ν_MAX_ (film)/cm^−1^; 2956, 1753, 1588, 1513, 1257, 1236, 1153, 1137, 1019, 825, 764. HRMS (ESI^+^) Found [M + H]^+^ 387.1806; C_22_H_27_O_6_ requires 387.1802. Values are in agreement with literature data [50].

*(±)-Bursehernin* (**1a**). Using general procedure C: Lactone **4ab** (0.114 g, 0.28 mmol) and a reaction time of 30 min to give triol **38ab** (0.111 g, 97%) as a cloudy oil. Then using general procedure D: Triol **38ab** (0.111 g, 0.27 mmol) and a reaction time of 1 h to give lactol **39ab** (0.093 g, 91%) which was used without further purification. Then using general procedure E: Lactol **39ab** (0.093 g, 0.25 mmol) and a reaction time of 2 h. The crude product was purified by column chromatography (1:1, hexanes, ethyl acetate) to give the title compound **1ab** (0.06 g, 65%) as a pale-yellow oil. R_f_ = 0.66 (19:1, CH_2_Cl_2_, methanol). δ_H_ (400 MHz; CDCl_3_) 2.41–2.62 (4H, m, 8, 7′, 8′-H), 2.88 (1H, dd, *J* = 14.0, 6.9 Hz, 7-H_A_), 2.96 (1H, dd, *J* = 14.0, 5.1 Hz, 7-H_B_), 3.82 (3H, s, 3-OCH_3_), 3.83–3.86 (4H, m, 4-OCH_3_, 9′-H_A_), 4.10 (1H, dd, *J* = 9.1, 6.9 Hz, 9′-H_B_), 5.91 (1H, d, *J* = 1.4 Hz, OCH_A_O), 5.92 (1H, d, *J* = 1.4 Hz, OCH_B_O), 6.42 (1H, d, *J* = 1.5 Hz, 2′-H), 6.44 (1H, dd, *J* = 7.9, 1.5 Hz, 6′-H), 6.66 (1H, d, *J* = 1.9 Hz, 2-H), 6.67–6.70 (2H, m, 6, 5′-H), 6.78 (1H, d, *J* = 8.0 Hz, 5-H). δ_C_ (100 MHz; CDCl_3_) 34.7 (C-7), 38.4 (C-7′), 41.2 (C-8′), 46.6 (C-8), 55.9 (3, 4-OCH_3_), 71.2 (C-9′), 101.1 (OCH_2_O), 108.4 (C-5′), 108.8 (C-2′), 111.2 (C-5), 112.3 (C-2), 121.4 (C-6), 121.6 (C-6′), 130.2 (C-1), 131.7 (C-1′), 146.4 (C-4′), 148.0 (C-3′), 148.1 (C-4), 149.2 (C-3), 178.7 (C-9). IR: ν_MAX_ (film)/cm^−1^; 2907, 1764, 1514, 1489, 1442, 1240, 1025, 923, 808, 730. HRMS (ESI^+^) Found [M + Na]^+^ 393.1317; C_21_H_22_NaO_6_ requires 393.1309. Values are in agreement with literature data [51].

*(±)-4-O-Methyl traxillagenin* (**1ac**). Using general procedure C: Lactone **4ac** (0.119 g, 0.27 mmol) and a reaction time of 45 min to give triol **38ac** (0.11 g, 90%) as a cloudy oil. The using general procedure D: Triol **38ac** (0.11 g, 0.24 mmol) and a reaction time of 15 min. The crude product was purified by column chromatography (1:2 hexanes, ethyl acetate) to give lactol **39ac** (0.06 g, 60%) as a colourless oil. Then using general procedure E: Lactol **39ac** (0.06 g, 0.15 mmol) and a reaction time of 3 h. The crude product purified by column chromatography (1:1, hexanes, ethyl acetate) to give the title compound **1ac** (0.044 g, 73%) as a white solid. R_f_ = 0.61 (19:1, CH_2_Cl_2_, methanol). Melting point: 126 °C. δ_H_ (400 MHz; CDCl_3_) 2.44–2.66 (4H, m, 8, 7′, 8′-H), 2.91 (1H, dd, *J* = 14.1, 6.6 Hz, 7-H_A_), 2.98 (1H, dd, *J* = 14.1, 5.4 Hz, 7-H_B_), 3.79 (6H, s, 3′-OCH_3_), 3.80 (6H, s, 4′-OCH_3_), 3.83 (3H, s, 3-OCH_3_), 3.84 (3H, s, 4-OCH_3_), 3.87 (1H, dd, *J* = 9.2, 7.3 Hz, 9′-H_A_), 4.14 (1H, dd, *J* = 9.2, 7.0 Hz, 9′-H_B_), 6.19 (2H, s, 2′-H), 6.63 (1H, dd, *J* = 8.0, 2.0 Hz, 6-H), 6.70 (1H, d, *J* = 2.0 Hz, 2-H), 6.75 (1H, d, *J* = 8.0 Hz, 5-H). δ_C_ (100 MHz; CDCl_3_) 34.6 (C-7), 39.0 (C-7′), 41.2 (C-8′), 46.7 (C-8), 56.0 (3, 4-OCH_3_), 56.2 (3′-OCH_3_), 60.9 (4′-OCH_3_), 71.3 (C-9′), 105.7 (C-2′), 111.2 (C-5), 112.6 (C-2), 121.4 (C-6), 130.3 (C-1), 133.8 (C-1′), 137.0 (C-4′), 148.1 (C-4), 149.2 (C-3), 153.5 (C-3′), 178.7 (C-9). IR: ν_MAX_ (film)/cm^−1^; 2938, 1764, 1590, 1509, 1460, 1237, 1123, 1014, 731. HRMS (ESI^+^) Found [M + Na]^+^ 439.1716; C_23_H_28_NaO_7_ requires 439.1727. Values are in agreement with literature data [52].

*(±)-4′-O-Benzyl buplerol* (**1ad**). Using general procedure C: Lactone **4ad** (0.505 g, 1.02 mmol) and a reaction time of 3 h to give the triol **38ad** (0.472 g, 93%) as a cloudy oil. Then using general procedure D: Triol **38ad** (0.472 g, 0.95 mmol) and a reaction time of 30 min to give lactol **39ad** (0.416 g, 94%) as a white solid which was used without further purification. Then using general procedure E: Lactol **39ad** (0.416 g, 0.90 mmol) and a reaction time of 1.5 h. The crude product was purified by column chromatography (1:1, hexanes, ethyl acetate) to give the title compound **1ad** (0.374 g, 90%) as a pale-yellow oil. R_f_ = 0.52 (1:1, hexanes, ethyl acetate). δ_H_ (400 MHz; CDCl_3_) 2.42–2.66 (4H, m, 8, 7′, 8′-H), 2.91 (1H, dd, *J* = 14.1, 6.2 Hz, 7-H_A_), 2.95 (1H, dd, *J* = 14.1, 5.7 Hz, 7-H_B_), 3.827, 3.829 (6H, 2 × s, 3, 3′-OCH_3_), 3.85 (3H, s, 4-OCH_3_), 3.83–3.88 (1H, m, 9′-H_A_), 4.11 (1H, dd, *J* = 8.7, 7.0 Hz, 9′-H_B_), 5.12 (2H, s, Ph-CH_2_), 6.48 (1H, dd, *J* = 8.0, 2.0 Hz, 6′-H), 6.51 (1H, d, *J* = 2.0 Hz, 2′-H), 6.64 (1H, dd, *J* = 8.2, 2.0 Hz, 6-H), 6.68 (1H, d, *J* = 2.0 Hz, 2-H), 6.76 (1H, d, *J* = 8.2 Hz, 5-H), 6.77 (1H, d, *J* = 8.0 Hz, 5′-H), 7.27–7.32 (1H, m, Ph-*p*-H), 7.33–7.38 (2H, m, Ph-*m*-H), 7.40–7.44 (2H, m, Ph-*o*-H). δ_C_ (100 MHz; CDCl_3_) 34.6 (C-7), 38.3 (C-7′), 41.2 (C-8′), 46.7 (C-8), 56.0 (3, 3′-OCH_3_), 56.1 (4-OCH_3_), 71.3, 71.4 (C-9′, Ph-CH_2_), 111.3 (C-5), 112.5 (C-2), 112.6 (C-5′), 114.5 (C-5′), 120.7 (C-6′), 121.5 (C-6), 127.4 (Ph-*o*-C), 128.0 (Ph-*p*-C), 128.7 (Ph-*m*-C), 130.3 (C-1), 131.3 (C-1′), 137.3 (Ph-*i*-C), 147.2 (C-4′), 148.1 (C-4), 149.2 (C-3), 149.9 (C-3′), 178.8 (C-9). IR: ν_MAX_ (film)/cm^−1^; 2935, 1763, 1512, 1260, 1233, 1140, 1014, 736, 697. HRMS (ESI^+^) Found [M + Na]^+^ 485.1934; C_28_H_30_NaO_6_ requires 485.1935.

*(±)-Buplerol* (**1ae**). Using general procedure F: Lactone **1ad** (0.336 g, 0.73 mmol) and a reaction time of 3.5 h to give the title compound **1ae** (0.271 g, quant.) as a white solid. R_f_ = 0.33 (1:1, hexanes, ethyl acetate). Melting point: 101–103 °C. δ_H_ (400 MHz; CDCl_3_) 2.42–2.66 (4H, m, 8, 7′, 8′-H), 2.90 (1H, dd, *J* = 14.1, 6.8 Hz, 7-H_A_), 2.97 (1H, dd, *J* = 14.1, 5.3 Hz, 7-H_B_), 3.81 (3H, s, 3-OCH_3_), 3.83 (3H, s, 3′-OCH_3_), 3.86 (4H, m, 4-OCH_3_), 3.87 (1H, dd, *J* = 8.9, 7.1 Hz, 9′-H_A_), 4.13 (1H, dd, *J* = 9.3, 7.1 Hz, 9′-H_B_), 5.51 (1H, s, 4′-OH), 6.43 (1H, d, *J* = 1.9 Hz, 2′-H), 6.52 (1H, dd, *J* = 8.0, 1.9 Hz, 6′-H), 6.64–6.67 (2H, m, 2, 6-H), 6.77 (1H, d, *J* = 8.6 Hz, 5-H), 6.80 (1H, d, *J* = 8.0 Hz, 5′-H). δ_C_ (100 MHz; CDCl_3_) 34.7 (C-7), 38.5 (C-7′), 41.3 (C-8′), 46.7 (C-8), 55.9, 56.0 (3, 3′, 4-OCH_3_), 71.4 (C-9′), 111.1, 111.2 (C-5, 5′), 112.5 (C-2), 114.6 (C-2′), 121.5 (C-6, 6′), 129.9 (C-1′), 130.4 (C-1), 144.6 (C-4′), 146.7 (C-3′), 148.1 (C-4), 149.2 (C-3), 178.9 (C-9). IR: ν_MAX_ (film)/cm^−1^; 3417, 2938, 1760, 1513, 1236, 1148, 1023, 812, 795. HRMS (ESI^+^) Found [M + Na]^+^ 395.1462; C_21_H_24_NaO_6_ requires 395.1465. Values are in agreement with literature data [53].

*(±)-Kusunokinin* (**1ba**). Using general procedure C: Lactone **4ba** (0.082 g, 0.20 mmol) and a reaction time of 1 h to give the triol **38ba** (0.083 g, quant.) as a cloudy oil. Then using general procedure D: Triol **38ba** (0.083 g, 0.20 mmol) and a reaction time of 15 min to give lactol **39ba** (0.064 g, 84%) which was used without further purification. Then using general procedure E: Lactol **39ba** (0.056 g, 0.15 mmol) and a reaction time of 1 h. The crude product was purified by column chromatography (2:1, hexanes, ethyl acetate) to give the title compound **1ba** (0.051 g, 91%) as a colourless oil. R_f_ = 0.48 (1:1, hexanes, ethyl acetate). δ_H_ (400 MHz; CDCl_3_) 2.44–2.65 (4H, m, 8, 7′, 8′-H), 2.84 (1H, dd, *J* = 14.1, 7.0 Hz, 7-H_A_), 2.95 (1H, dd, *J* = 14.1, 5.1 Hz, 7-H_B_), 3.82 (3H, s, 3′-OCH_3_), 3.85 (3H, s, 4′-OCH_3_), 3.87 (1H, dd, *J* = 9.2, 7.2 Hz, 9′-H_A_), 4.14 (1H, dd, *J* = 9.2, 7.0 Hz, 9′-H_B_), 5.92 (1H, d, *J* = 1.4 Hz, OCH_A_O), 5.93 (1H, d, *J* = 1.4 Hz, OCH_B_O), 6.48 (1H, d, *J* = 2.0 Hz, 2′-H), 6.55–6.60 (3H, m, 2, 6, 6′-H), 6.71 (1H, d, *J* = 7.7 Hz, 5-H), 6.76 (1H, d, *J* = 8.2 Hz, 5′-H). δ_C_ (100 MHz; CDCl_3_) 34.9 (C-7), 38.4 (C-7′), 41.3 (C-8′), 46.6 (C-8), 55.9 (3′-OCH_3_), 56.0 (4′-OCH_3_), 71.4 (C-9′), 101.1 (OCH_2_O), 108.3 (C-5), 109.6 (C-2), 111.4 (C-5′), 111.8 (C-2′), 120.8 (C-6′), 122.4 (C-6), 130.6 (C-1′), 131.5 (C-1), 146.6 (C-4), 148.0 (C-3, 4′), 149.2 (C-3′), 178.6 (C-9). IR: ν_MAX_ (film)/cm^−1^; 2908, 1764, 1515, 1489, 1442, 1242, 1024, 912, 809, 729. HRMS (ESI^+^) Found [M + Na]^+^ 393.1301; C_21_H_22_NaO_6_ requires 393.1309. Values are in agreement with literature data [50].

*(±)-Hinokinin* (**1bb**). Using general procedure C: Lactone **4bb** (0.12 g, 0.31 mmol) and a reaction time of 30 min to give the triol **38bb** (0.12 g, quant.) as a cloudy oil. Then using general procedure D: Triol **38bb** (0.121 g, 0.31 mmol) and a reaction time of 10 min to give lactol **39bb** (0.096 g, 86%) which was used without further purification. Then using general procedure E: Lactol **39bb** (0.089 g, 0.25 mmol) and a reaction time of 1 h. The crude product was purified by column chromatography (1:1, hexanes, ethyl acetate) to give the title compound **1bb** (0.08 g, 90%) as a pale-yellow oil. R_f_ = 0.73 (1:1, hexanes, ethyl acetate). δ_H_ (400 MHz; CDCl_3_) 2.41–2.62 (4H, m, 8, 7′, 8′-H), 2.83 (1H, dd, *J* = 14.1, 7.2 Hz, 7-H_A_), 2.98 (1H, dd, *J* = 14.1, 5.0 Hz, 7-H_B_), 3.85 (1H, dd, *J* = 9.2, 7.1 Hz, 9′-H_A_), 4.12 (1H, dd, *J* = 9.2, 6.9 Hz, 9′-H_B_), 5.91–5.94 (4H, m, 2 × OCH_2_O), 6.44–6.47 (2H, m, 2′, 6′-H), 6.59 (1H, dd, *J* = 7.9, 1.8 Hz, 6-H), 6.62 (1H, d, *J* = 1.8 Hz, 2-H), 6.69 (1H, d, *J* = 8.4 Hz, 5′-H), 6.72 (1H, d, *J* = 7.9 Hz, 5-H). δ_C_ (100 MHz; CDCl_3_) 34.9 (C-7), 38.4 (C-7′), 41.4 (C-8′), 46.6 (C-8), 71.2 (C-9′), 101.1 (2 × OCH_2_O), 108.4 (C-5, 5′), 108.9 (C-2′), 109.5 (C-2), 121.6 (C-6′), 122.3 (C-6), 131.5 (C-1), 131.7 (C-1′), 146.4 (C-4), 146.6 (C-4′), 148.0 (C-3, 3′), 178.5 (C-9). IR: ν_MAX_ (film)/cm^−1^; 2901, 1764, 1488, 1441, 1242, 1015, 924, 808, 728. HRMS (ESI^+^) Found [M + Na]^+^ 377.0986; C_20_H_18_NaO_6_ requires 377.0996. Values are in agreement with literature data [54].

*(±)-Isoyatein* (**1bc**). Using general procedure C: Lactone **4bc** (0.076 g, 0.18 mmol) and a reaction time of 1 h to give the triol **38bc** (0.077 g, >99%) as a cloudy oil. Then using general procedure D: Triol **38bc** (0.077 g, 0.18 mmol) and a reaction time of 1 h to give lactol **39bc** (0.057 g, 80%) which was used without further purification. Then using general procedure E: Lactol **39bc** (0.05 g, 0.12 mmol) and a reaction time of 3 h. The crude product was purified by column chromatography (1:1, hexanes, ethyl acetate) to give the title compound **1bc** (0.8 mg, 16%) as a pale-yellow oil. R_f_ = 0.55 (1:1, hexanes, ethyl acetate). δ_H_ (400 MHz; CDCl_3_) 2.46–2.64 (4H, m, 8, 7′, 8′-H), 2.86 (1H, dd, *J* = 14.1, 7.0 Hz, 7-H_A_), 2.98 (1H, dd, *J* = 14.1, 5.1 Hz, 7-H_B_), 3.81 (6H, s, 3′-OCH_3_), 3.82 (3H, s, 4′-OCH_3_), 3.89 (1H, dd, *J* = 9.2, 7.0 Hz, 9′-H_A_), 4.19 (1H, dd, *J* = 9.2, 6.8 Hz, 9′-H_B_), 5.93 (1H, d, *J* = 1.5 Hz, OCH_A_O), 5.94 (1H, d, *J* = 1.5 Hz, OCH_B_O), 6.20 (2H, s, 2′-H), 6.58 (1H, dd, *J* = 7.9, 1.8 Hz, 6-H), 6.61 (1H, d, *J* = 1.8 Hz, 2-H), 6.71 (1H, d, *J* = 7.9 Hz, 5-H). δ_C_ (100 MHz; CDCl_3_) 34.9 (C-7), 39.2 (C-7′), 41.4 (C-8′), 46.6 (C-8), 56.2 (3′-OCH_3_), 61.0 (4′-OCH_3_), 71.4 (C-9′), 101.2 (OCH_2_O), 105.7 (C-2′), 108.3 (C-5), 109.6 (C-2), 122.4 (C-6), 131.5 (C-1), 133.8 (C-1′), 137.0 (C-4′), 146.7 (C-4), 148.1 (C-3), 153.5 (C-3′), 178.5 (C-9). IR: ν_MAX_ (film)/cm^−1^; 2938, 1763, 1590, 1489, 1443, 1241, 1122, 1011, 927, 813, 732. HRMS (ESI^+^) Found [M + Na]^+^ 423.1400; C_22_H_24_NaO_7_ requires 423.1414. Values are in agreement with literature data [55].

*(±)-4′-O-Benzyl haplomyrfolin* (**1bd**). Using general procedure C: Lactone **4bd** (0.18 g, 0.38 mmol) and a reaction time of 20 min to give the triol **38bd** (0.18 g, quant.) as a cloudy oil. Then using general procedure D: Triol **38bd** (0.18 g, 0.38 mmol) and a reaction time of 20 min to give lactol **39bd** (0.13 g, 76%) as a white solid which was used without further purification. Then using general procedure E: Lactol **39bd** (0.13 g, 0.28 mmol) and a reaction time of 2 h. The crude product was purified by column chromatography (3:1, hexanes, ethyl acetate) to give the title compound **1bd** (0.12 g, 94%) as a colourless oil. R_f_ = 0.65 (1:1, hexanes, ethyl acetate). δ_H_ (400 MHz; CDCl_3_) 2.43–2.64 (4H, m, 8, 7′, 8′-H), 2.84 (1H, dd, *J* = 14.1, 7.0 Hz, 7-H_A_), 2.94 (1H, dd, *J* = 14.1, 5.1 Hz, 7-H_B_), 3.83 (3H, s, 3′-OCH_3_), 3.87 (1H, dd, *J* = 9.1, 7.2 Hz, 9′-H_A_), 4.14 (1H, dd, *J* = 9.1, 7.0 Hz, 9′-H_B_), 5.12 (2H, s, 7″-H), 5.91 (1H, d, *J* = 1.4 Hz, OCH_A_O), 5.93 (1H, d, *J* = 1.4 Hz, OCH_B_O), 6.49–6.52 (2H, m, 2′, 6′-H), 6.57 (1H, dd, *J* = 7.9, 1.8 Hz, 6-H), 6.59 (1H, d, *J* = 1.8 Hz, 2-H), 6.70 (1H, d, *J* = 7.9 Hz, 5-H), 6.78 (1H, d, *J* = 8.5 Hz, 5′-H), 7.27–7.32 (1H, m, 4″-H), 7.33–7.38 (2H, m, 3″-H), 7.41–7.45 (2H, m, 2″-H). δ_C_ (100 MHz; CDCl_3_) 34.8 (C-7), 38.4 (C-7′), 41.3 (C-8′), 46.5 (C-8), 56.0 (3′-OCH_3_), 71.2 (C-7″), 71.3 (C-9′), 101.1 (OCH_2_O), 108.3 (C-5), 109.6 (C-2), 112.4 (C-2′), 114.4 (C-5′), 120.7 (C-6′), 122.4 (C-6), 127.4 (C-2″), 128.0 (C-4″), 128.6 (C-3″), 131.2 (C-1), 131.5 (C-1′), 137.2 (C-1″), 146.6 (C-4), 147.1 (C-4′), 148.0 (C-3), 149.9 (C-3′), 178.6 (C-9). IR: ν_MAX_ (film)/cm^−1^; 2907, 1765, 1504, 1489, 1443, 1244, 1140, 1034, 911, 809, 730. HRMS (ESI^+^) Found [M + Na]^+^ 469.1612; C_27_H_26_NaO_6_ requires 469.1622.

*(±)-Haplomyrfolin* (**1be**). Using general procedure F: Lactone **1bd** (0.119 g, 0.27 mmol) and a reaction time of 1.5 h. The crude product was purified by column chromatography (1:1 hexanes, ethyl acetate) to give the title compound **1be** (0.086 g, 91%) as a colourless oil. R_f_ = 0.47 (1:1 hexanes, ethyl acetate). δ_H_ (400 MHz; CDCl_3_) 2.43–2.63 (4H, m, 8, 7′, 8′-H), 2.84 (1H, dd, *J* = 14.1, 7.0 Hz, 7-H_A_), 2.95 (1H, dd, *J* = 14.1, 5.2 Hz, 7-H_B_), 3.83 (3H, s, 3′-OCH_3_), 3.86 (1H, dd, *J* = 9.1, 7.2 Hz, 9′-H_A_), 4.13 (1H, dd, *J* = 9.1, 7.0 Hz, 9′-H_B_), 5.63 (1H, s, 4′-OH), 5.91 (1H, d, *J* = 1.4 Hz, OCH_A_O), 5.92 (1H, d, *J* = 1.4 Hz, OCH_B_O), 6.46 (1H, d, *J* = 1.9 Hz, 2′-H), 6.51 (1H, dd, *J* = 8.0, 1.9 Hz, 6′-H), 6.58 (1H, dd, *J* = 7.8, 1.7 Hz, 6-H), 6.60 (1H, d, *J* = 1.7 Hz, 2-H), 6.70 (1H, d, *J* = 7.8 Hz, 5-H), 6.80 (1H, d, *J* = 8.0 Hz, 5′-H). δ_C_ (100 MHz; CDCl_3_) 34.8 (C-7), 38.3 (C-7′), 41.4 (C-8′), 46.5 (C-8), 55.9 (3′-OCH_3_), 71.3 (C-9′), 101.1 (OCH_2_O), 108.3 (C-5), 109.6 (C-2), 111.2 (C-2′), 114.6 (C-5′), 121.4 (C-6′), 122.4 (C-6), 129.9 (C-1′), 131.5 (C-1), 144.5 (C-4′), 146.5 (C-4), 146.7 (C-3′), 147.9 (C-3), 178.7 (C-9). IR: ν_MAX_ (film)/cm^−1^; 3468, 2921, 1762, 1515, 1489, 1443, 1243, 1035, 907, 725. HRMS (ESI^+^) Found [M + Na]^+^ 379.1151; C_20_H_20_NaO_6_ requires 379.1152. Values are in agreement with literature data [56].

## 4. Biological Assay Methods

### 4.1. Cell Culture

Jurkat E61 cells (ECACC) were maintained at 37 °C in RMPI media (Lonza) supplemented with 10% Foetal Bovine Serum (FBS) (Lonza) (10% RPMI) in a humidified environment of 5% CO_2_ in air. Cells were routinely passaged to maintain a cell density of between 1 × 10^5^ and 1 × 10^6^/mL.

### 4.2. Drug Treatments

Lignans were diluted to stock concentrations of 30 mM in DMSO and further diluted to the working concentration in 10% RPMI. The DMSO diluted to the appropriate concentration was used as the vehicle-control. Cells were seeded at the relevant density per well depending upon the assay to be performed, in 100 µL volume of fresh 10% RPMI. Trypan blue exclusion method was used to assess viability prior to experiments and cell viability was always >95%. Lignans were added at 100 µL/well to the relevant wells. Cells were incubated at 37 °C in a humidified environment of 5% CO_2_ in air for the indicated times. Dead cell controls were included in subsequent viability assays by treating cells with 50 µL/well EtOH (final concentration 50%) for 48 h. Apoptotic controls were included in subsequent apoptosis assays by exposing cells to a heat shock at 43 °C for 2 h. Positive controls for cell cycle analysis were included by treating cells with 0.5 μM camptothecin for 4 h to induce cell cycle arrest.

### 4.3. MTS Assay

Following treatments at a cell density of 1 × 10^5^ cells/well, the samples were centrifuged at 500 *g* for 5 min and the supernatant was removed. A 100 µL/well volume of fresh 10 % RPMI was added. A 20 µL volume of MTS solution (Promega, G1112) was added to each well and the plate was incubated in the dark for 1 h at 37 °C. The absorbance was detected at 490 nm on a Synergy HT plate reader.

### 4.4. Annexin V/PI Assay

Following treatments at a cell density of 1 × 10^5^ cells/well, the samples were centrifuged at 500 *g* for 5 min and the supernatant was removed. Cells were washed in 500 µL DPBS before addition of 100 µL of 1 × Annexin V binding buffer (BD Biosciences). A 5 µL volume of FITC-conjugated Annexin V (BD Biosciences) and 10 µL Propidium Iodide (BD Biosciences) was added and the cells were incubated in the dark for 20 min. Samples were diluted by addition of 400 µL 1 × Annexin V binding buffer before immediate analysis on an Accuri C6 Flow Cytometer (Becton Dickinson, Oxford, UK).

### 4.5. Cell Cycle Analysis

Following treatments at a cell density of 5 × 10^6^/well, cells were centrifuged at 500 *g* for 5 min and the supernatant was removed. The remaining cell pellet was vortexed while simultaneously adding 500 μL of 70% ethanol dropwise, fixing the cells and minimising clumping. The samples were incubated at 4 °C for 30 min, and then centrifuged at 1000 *g* for 5 min. The supernatant was discarded, and the pellet was re-suspended in 500 μL DPBS. The samples were centrifuged again at 1000 *g* for 5 min, and the supernatant was removed a final time. The pellet was resuspended in 50 μL RNase A (100 μg/mL stock; Roche, UK) and 200 μL PI (50 μg/mL stock; Sigma, UK). The samples were analyzed on an Accuri C6 flow cytometer (Becton Dickinson) and data was modelled and interpreted using ModFit Analysis Software, version 5.0 (Verity Software House).

## Supplementary Materials

The following are available online.
