# Supplementary material for: Modular Synthesis and Biological Investigation of 5-Hydroxymethyl Dibenzyl Butyrolactones and Related Lignans
Source: Molecules. 2018 Nov 22;23(12):3057. doi: 10.3390/molecules23123057 (PMC6321111; doi:10.3390/molecules23123057)

Supporting Information:

Modular Synthesis and Biological Investigation of 3,4-dibenzyl-5-(hydroxymethyl)dihydrofuran-2(3*H*)-ones and Related Lignans

(*E*)-Ethyl 4-(3′,4′-dimethoxyphenyl)but-2-enoate **16**

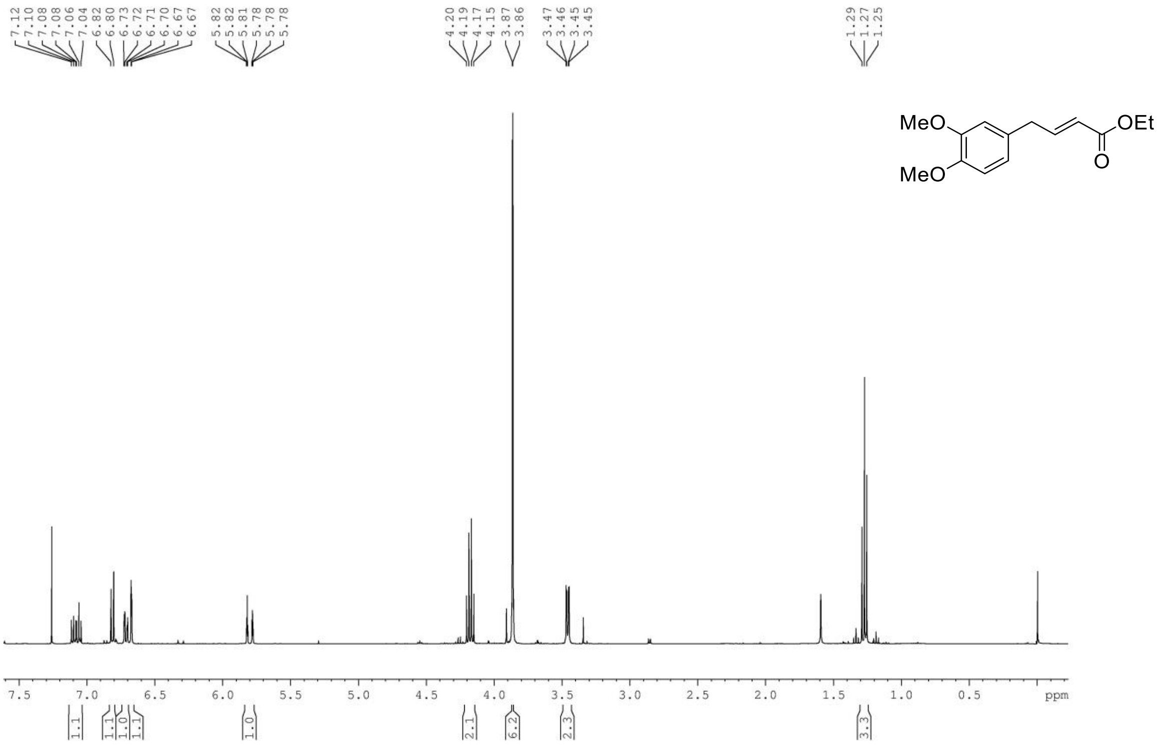

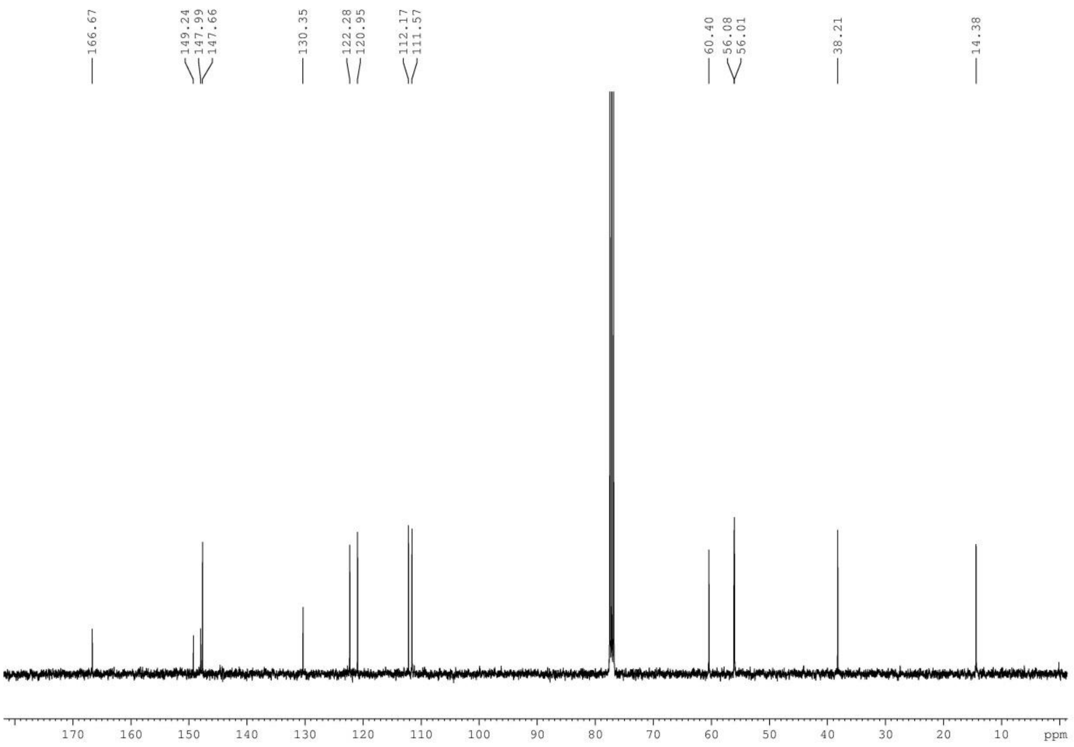

(*E*)-4-(3′,4′-Dimethoxyphenyl)but-2-en-1-ol **18**

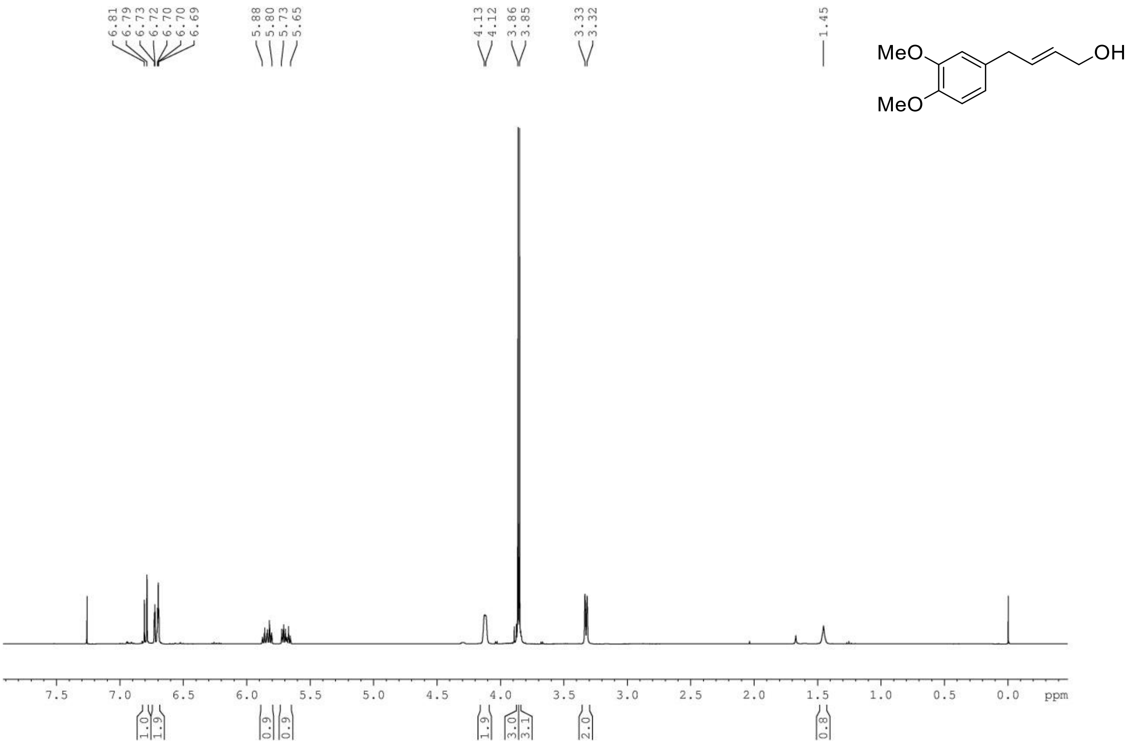

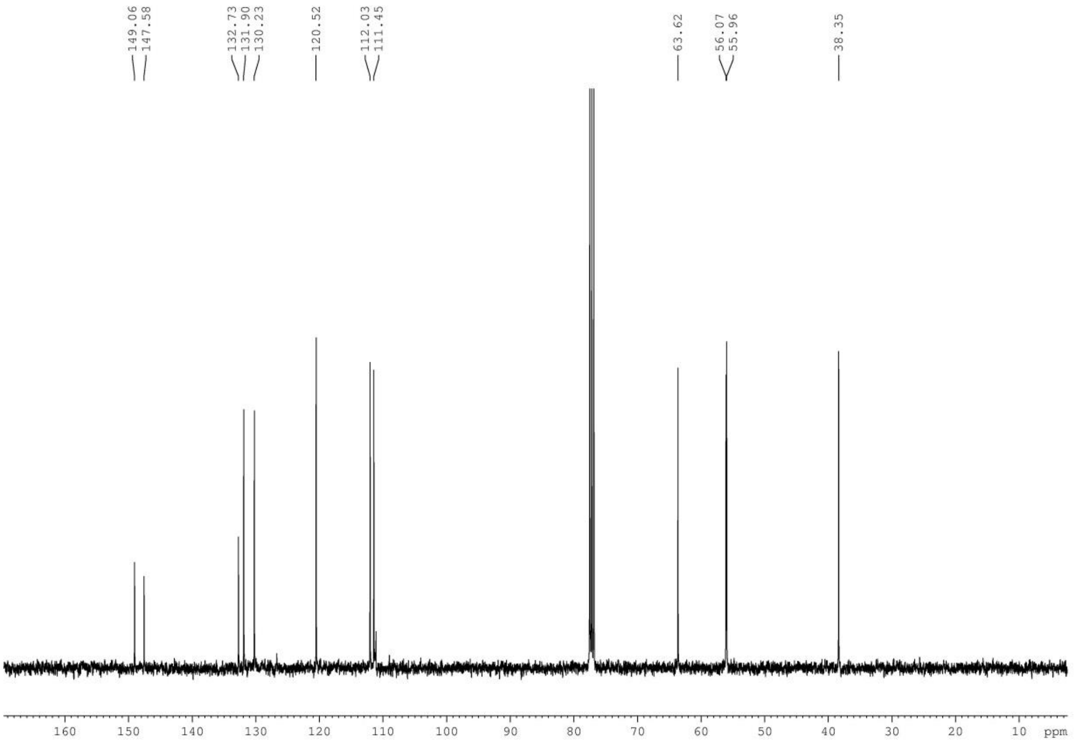

(*E*)-4-(4-(3′,4′-Dimethoxyphenyl)but-2-en-1-yl)morpholine **9a**

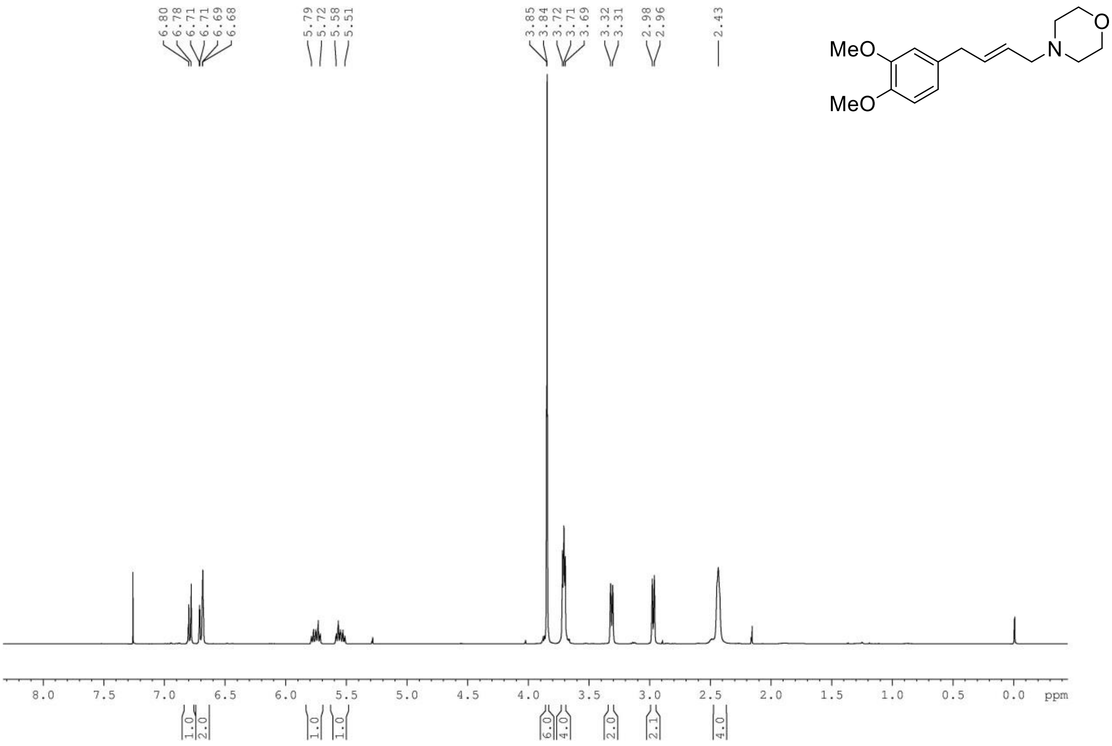

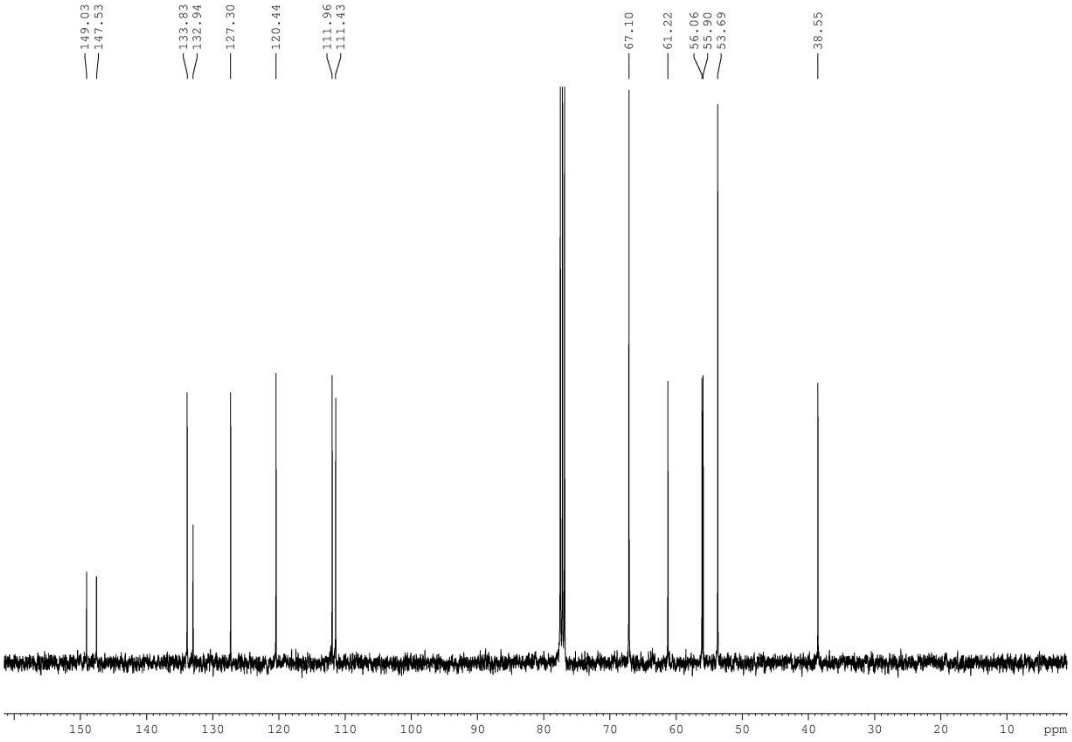

(*E*)-Ethyl 4-(3′,4′-methylenedioxyphenyl)but-2-enoate **17**

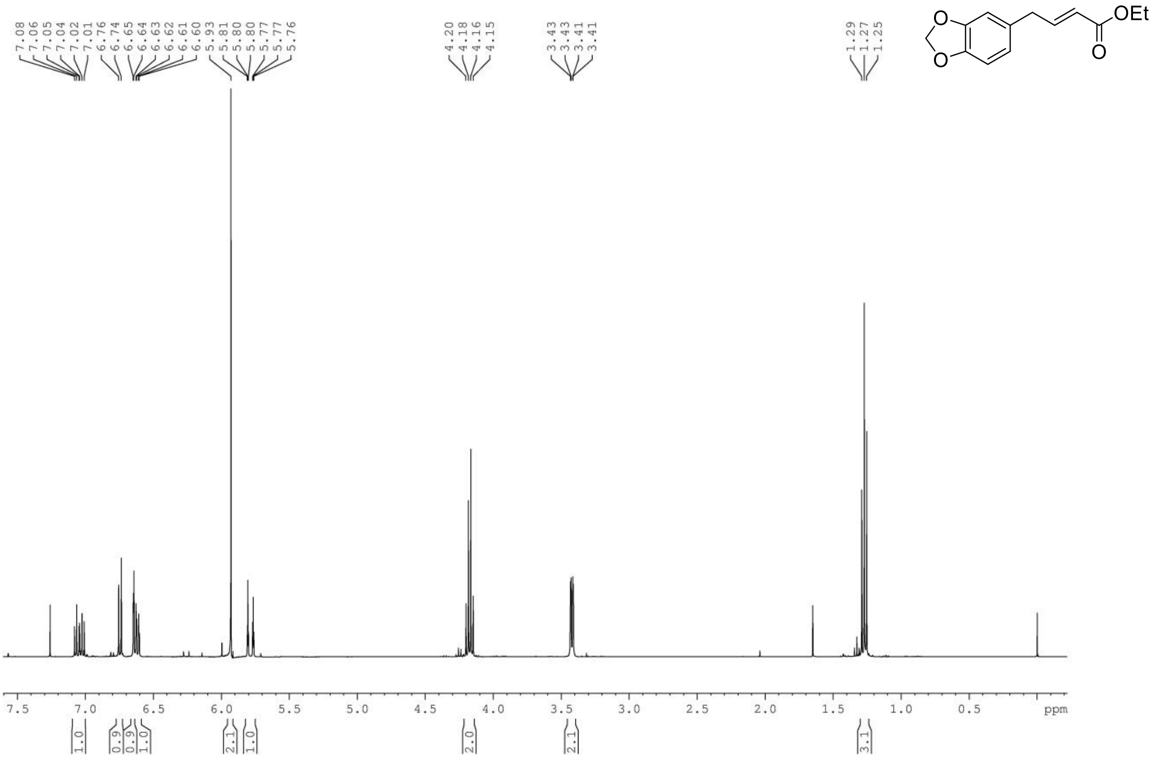

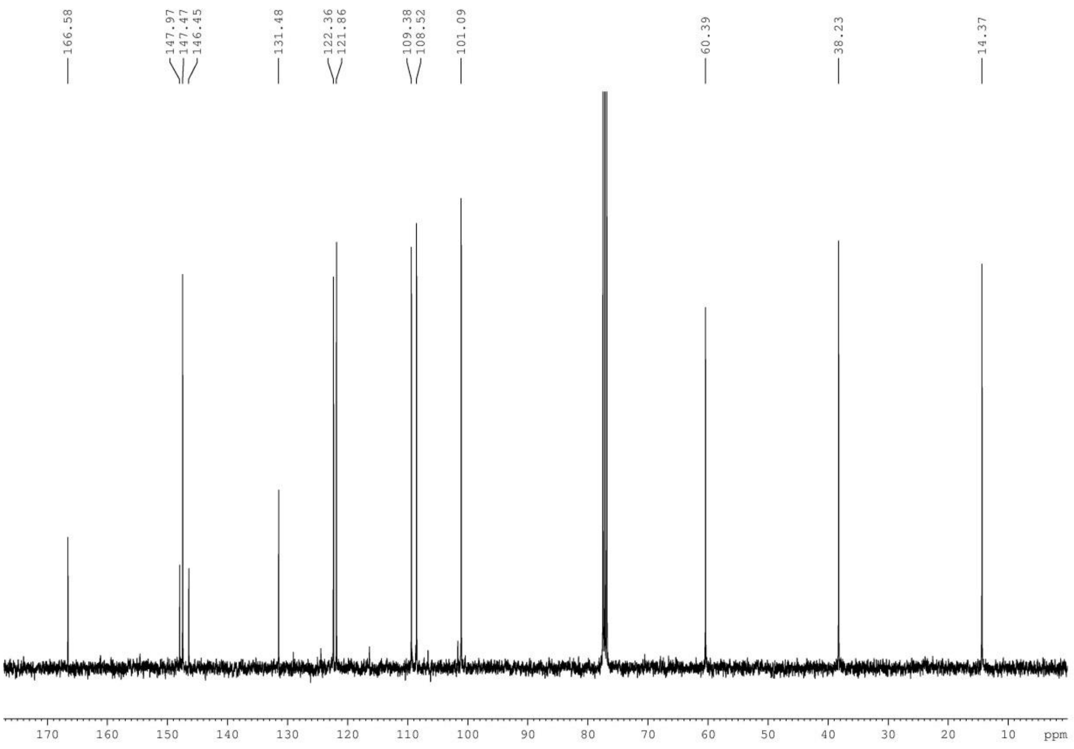

(*E*)-4-(3′,4′-Methylenedioxyphenyl)but-2-en-1-ol **19**

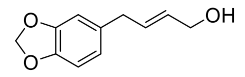

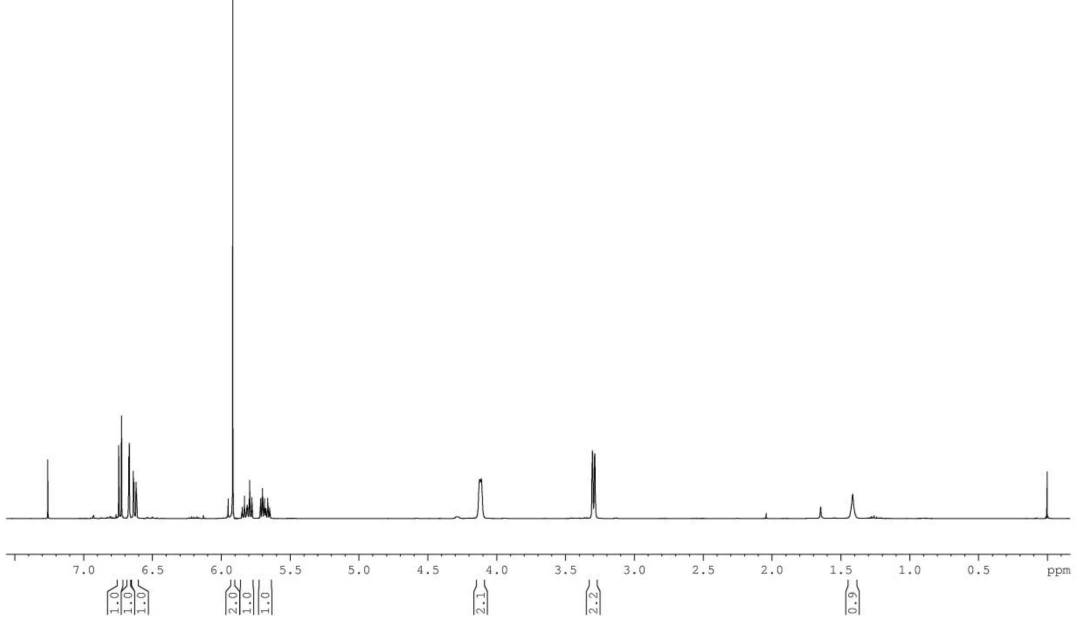

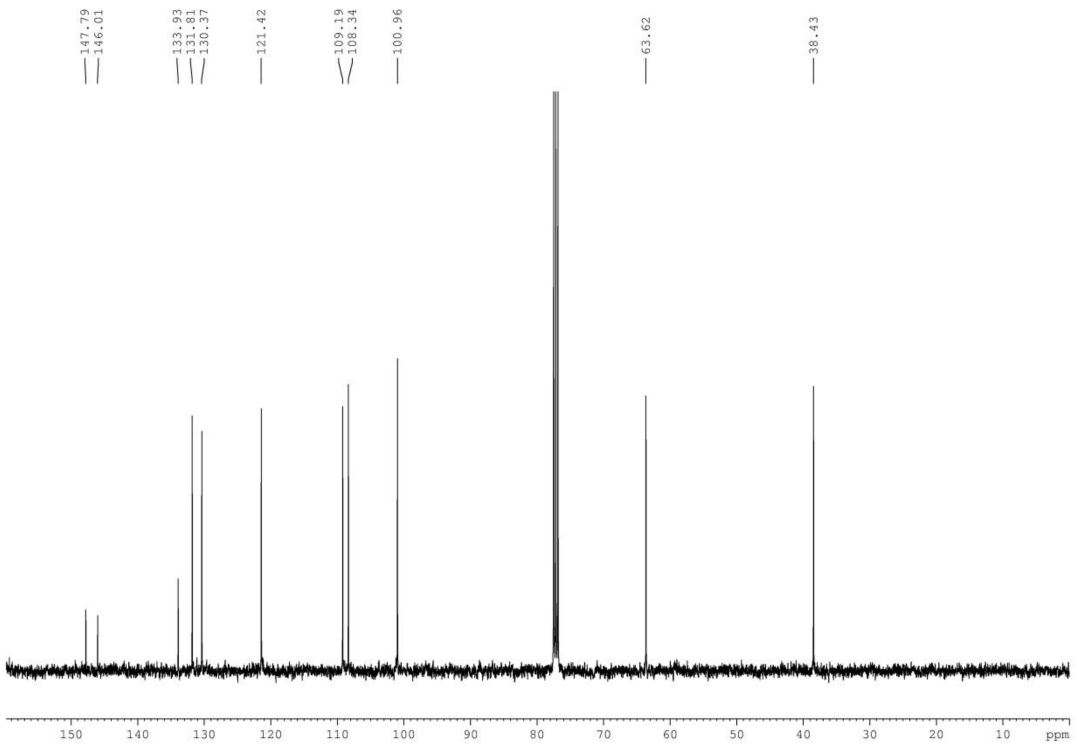

(*E*)-4-(4-(3′,4′-Methylenedioxyphenyl)but-2-en-1-yl)morpholine **9b**

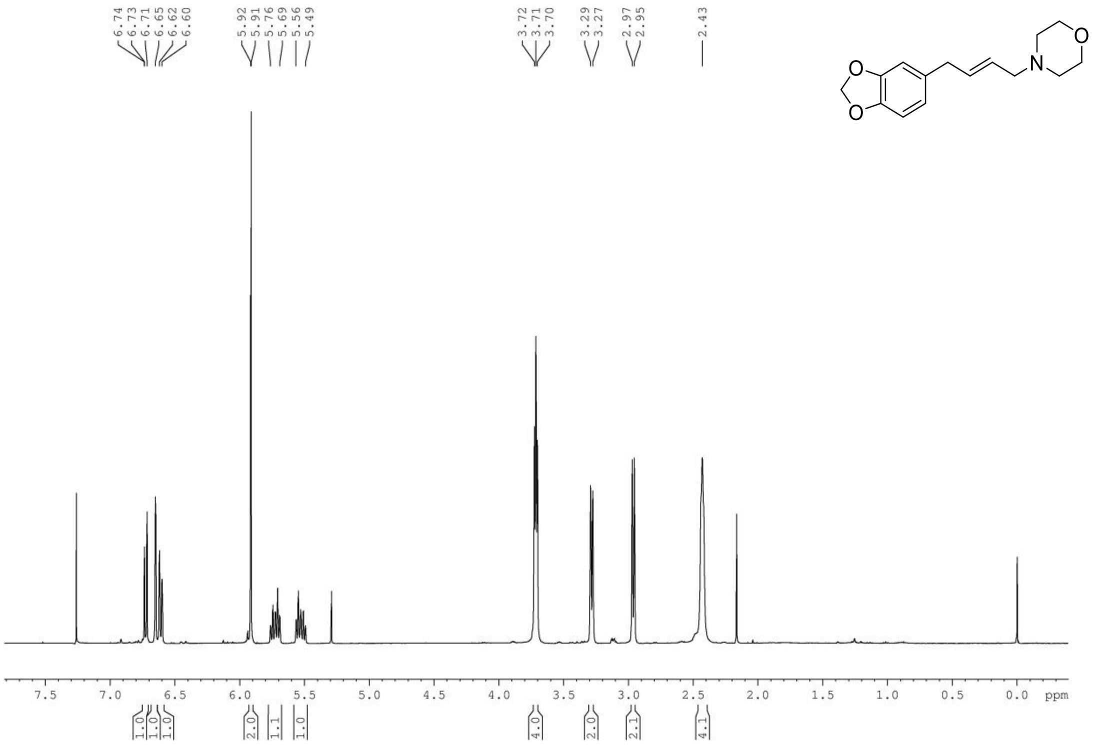

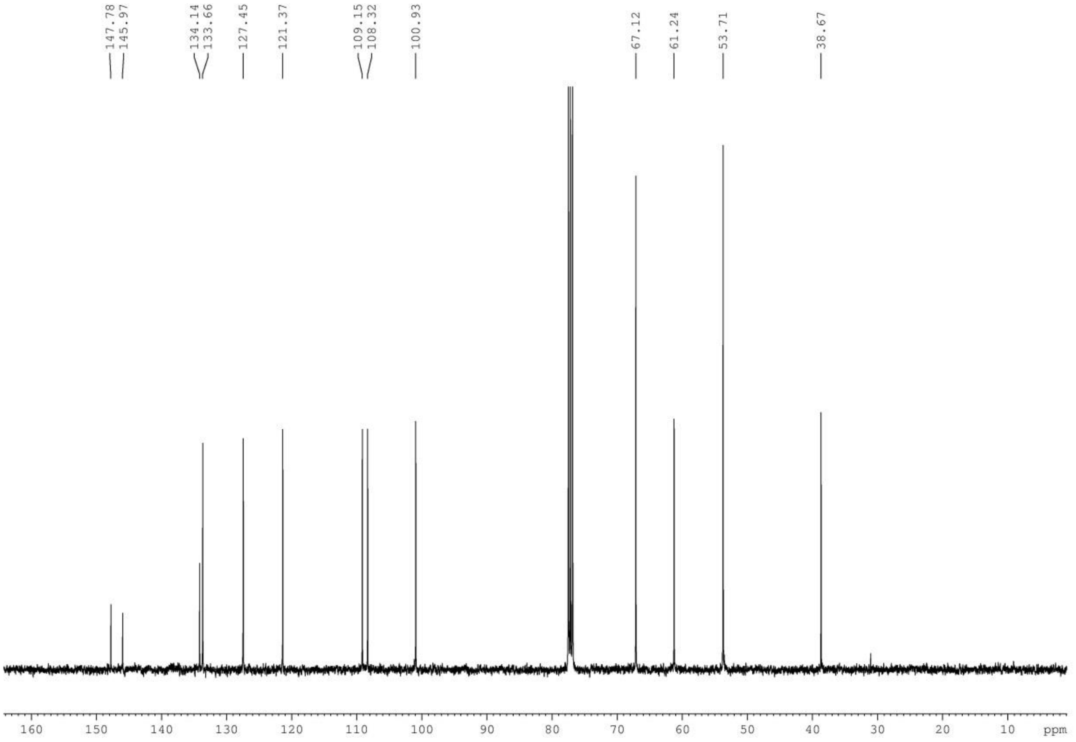

3-(3′,4′,5′-Trimethoxyphenyl)propionic acid **31**

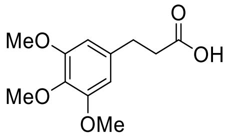

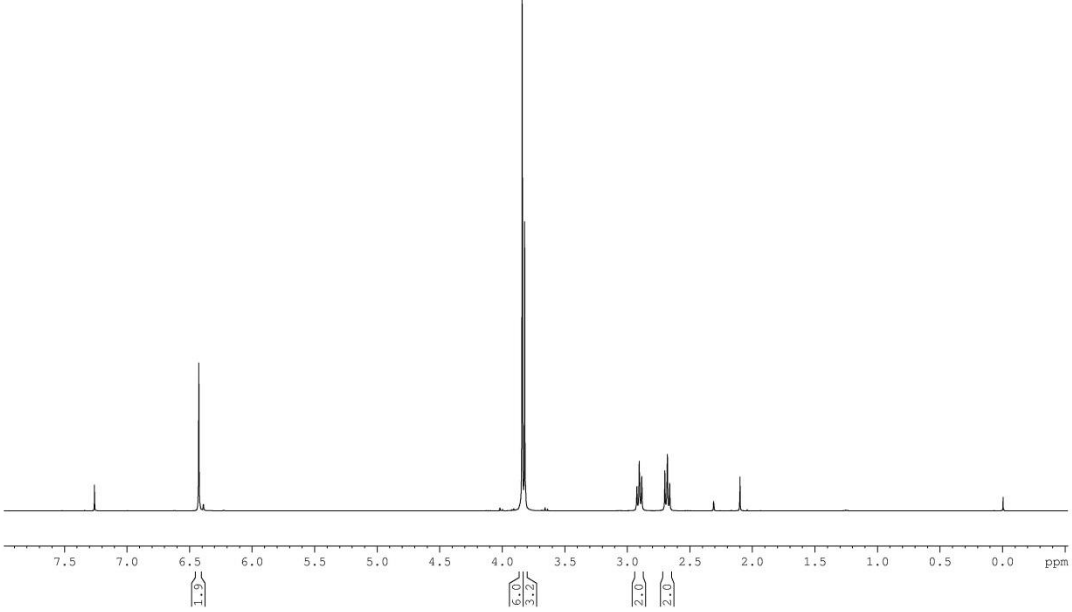

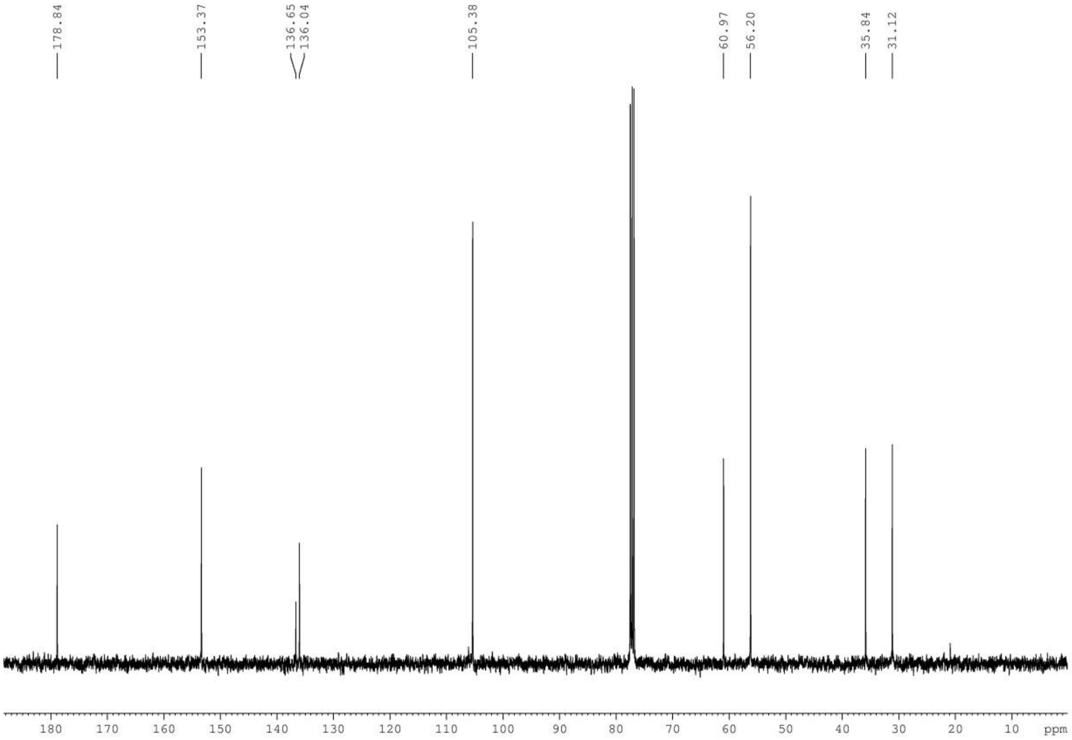

3-(3′,4′-Methylenedioxyphenyl)propionic acid **30**

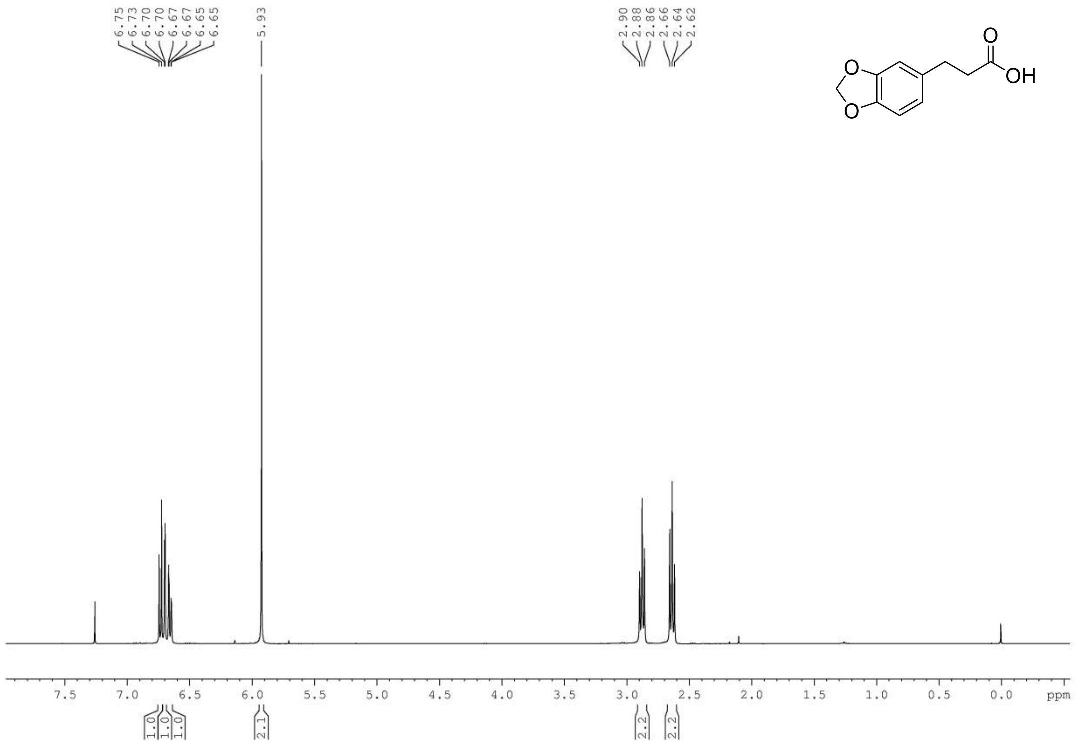

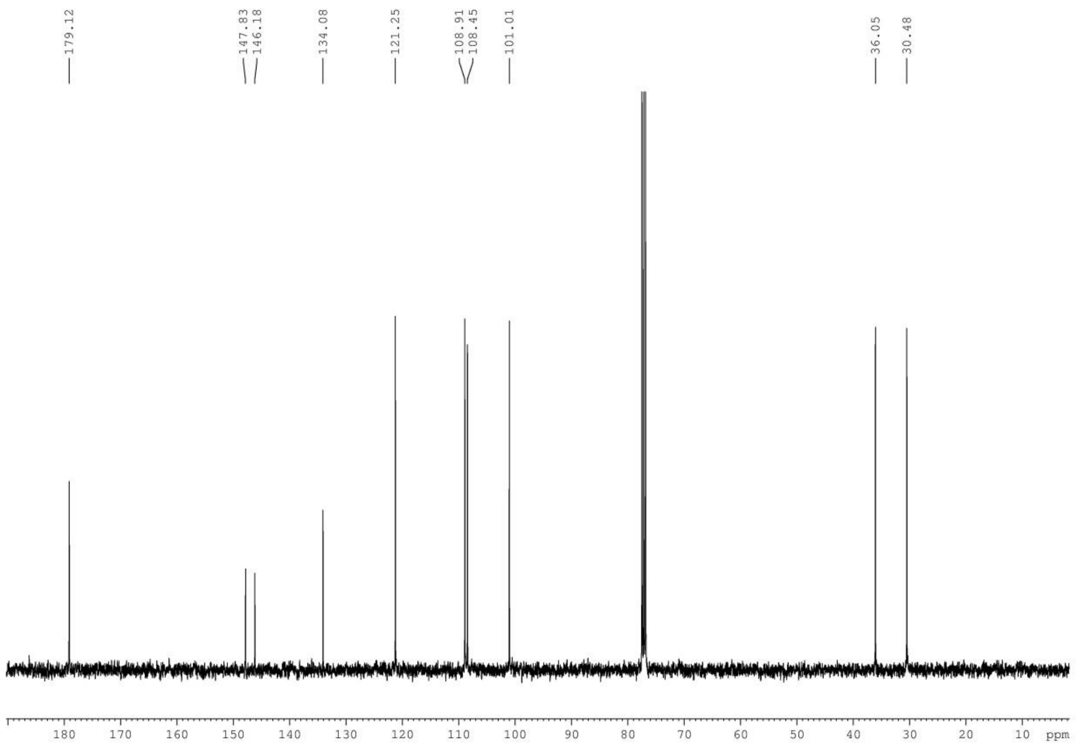

3-(3′-Methoxy-4′-benzyloxyphenyl)propionic acid **32**

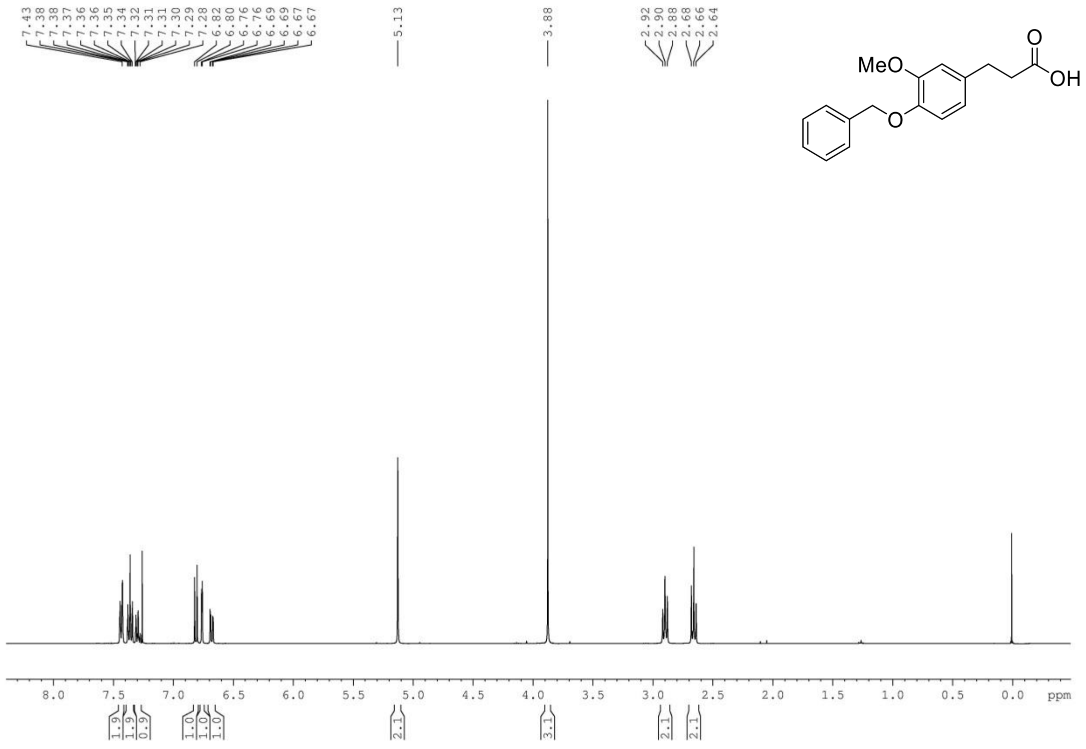

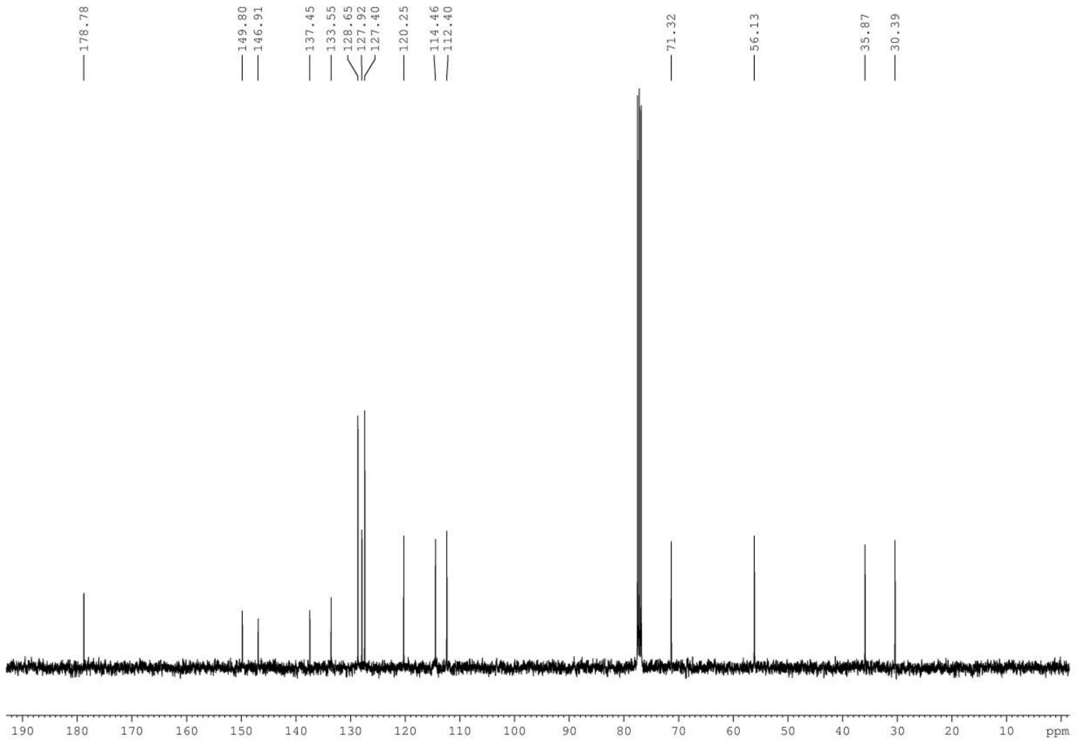

(2*R**,3*S**)-2-(3′,4′-Methylenedioxybenzyl)-3-(3″,4″-dimethoxybenzyl)-1-morpholinopent-4-en-1-one **35ab**

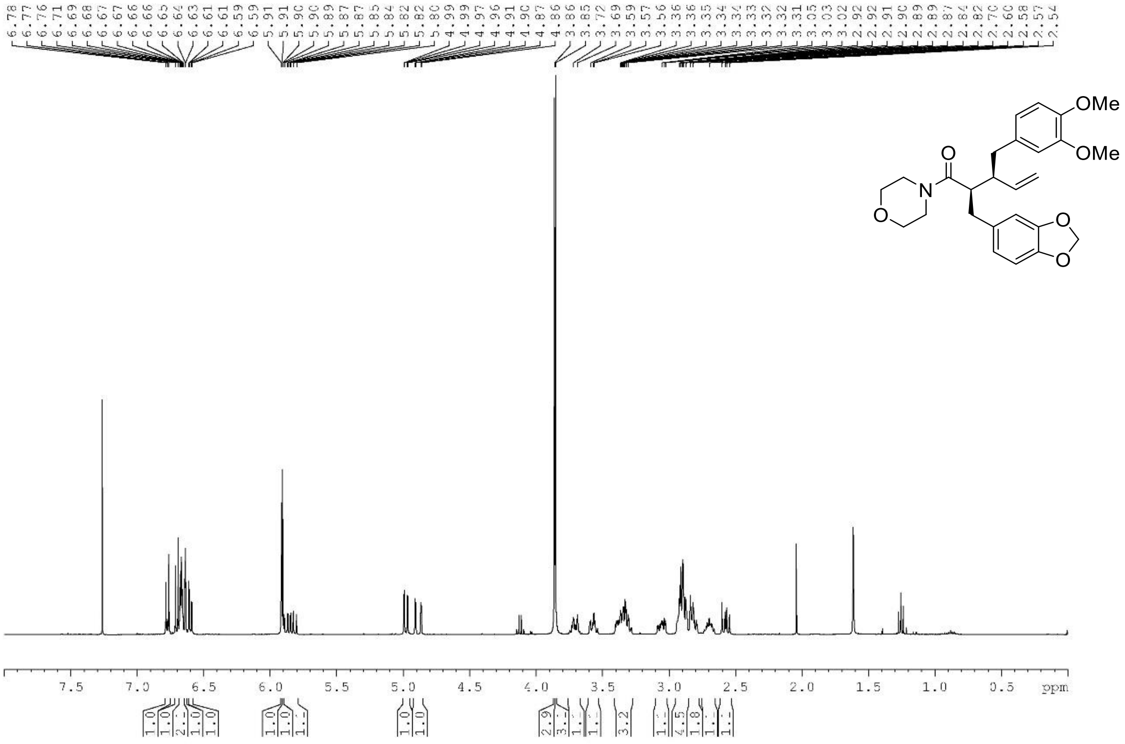

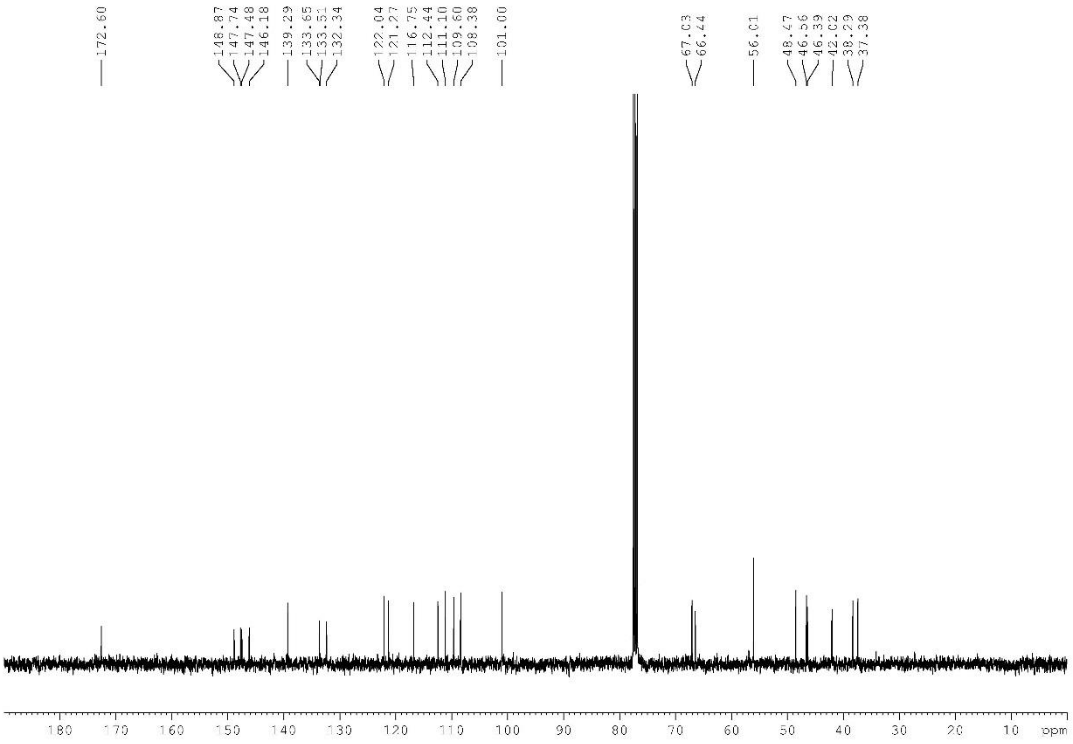

(2*R**,3*S**)-2-(3′,4′,5′-Trimethoxybenzyl)-3-(3″,4″-dimethoxybenzyl)-1-morpholinopent-4-en-1-one **35ac**

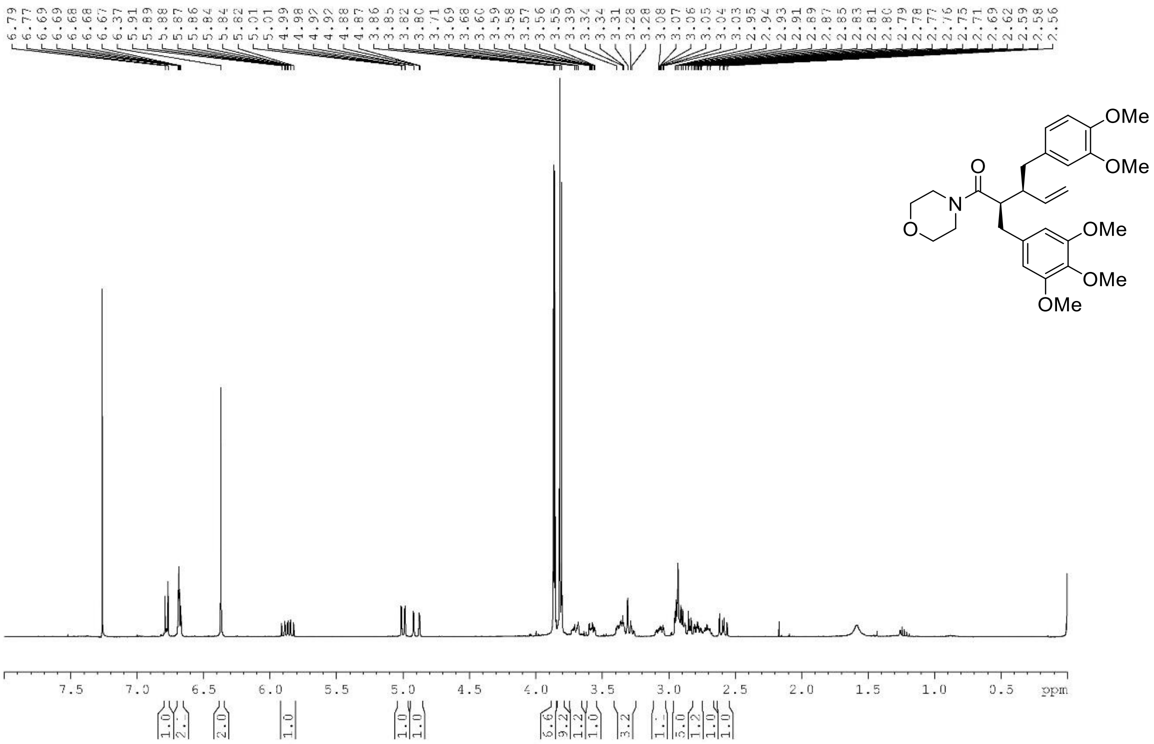

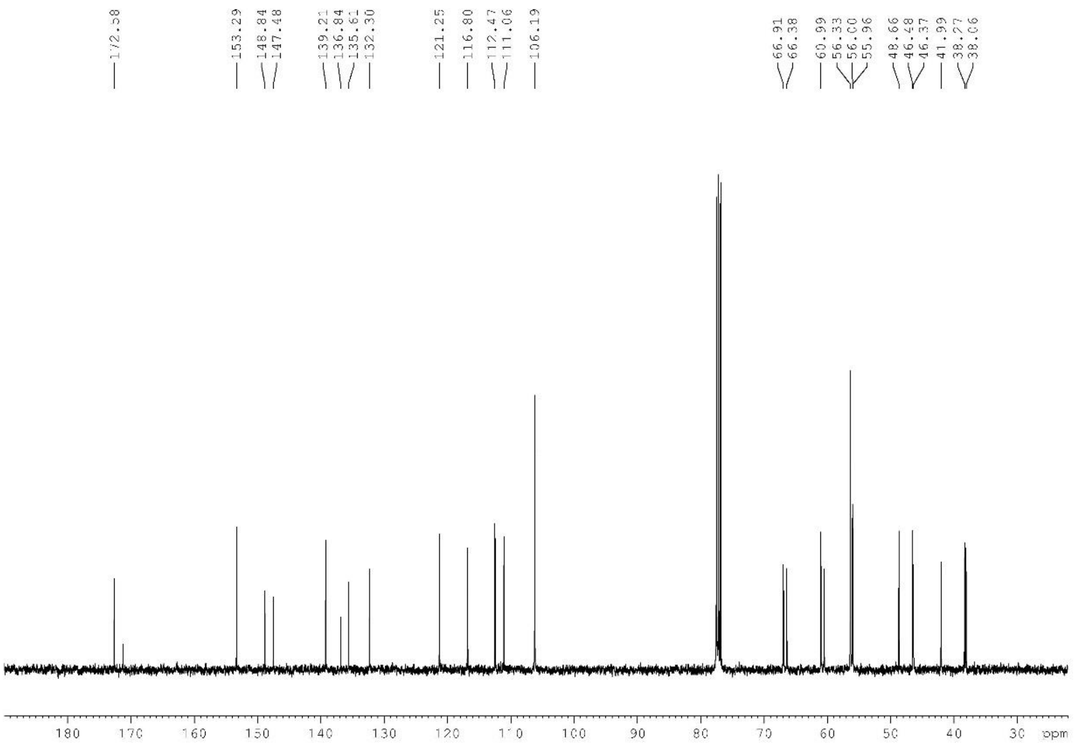

(2*R**,3*S**)-2-(3′,4′-Dimethoxybenzyl)-3-(3″,4″-dimethoxybenzyl)-1-morpholinopent-4-en-1-one **35aa**

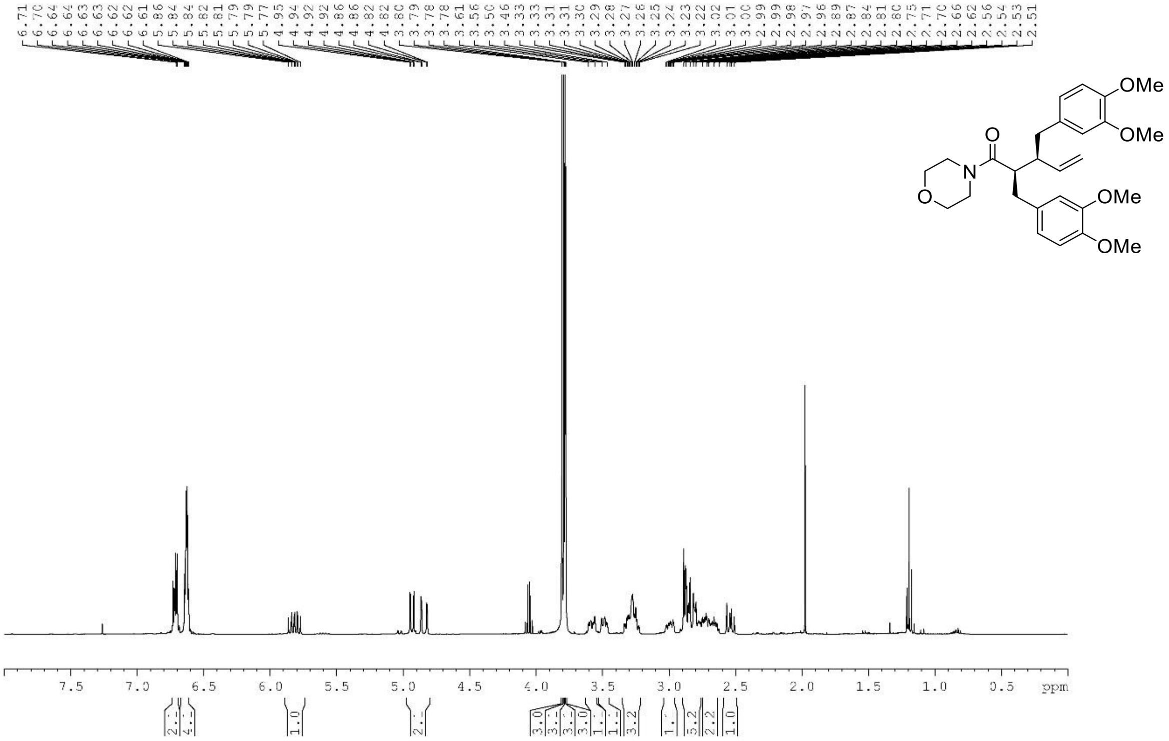

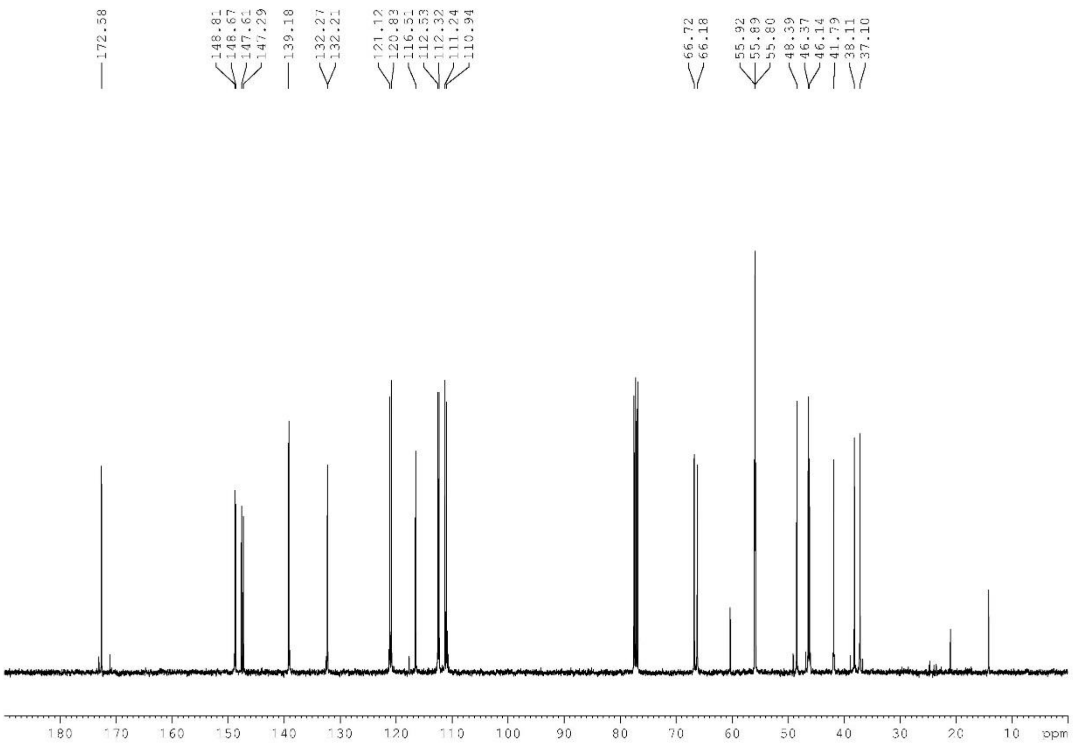

(2*R**,3*S**)-2-(3′-Methoxy-4′-benzyloxybenzyl)-3-(3″,4″-dimethoxybenzyl)-1-morpholino-pent-4-en-1-one **35ad**

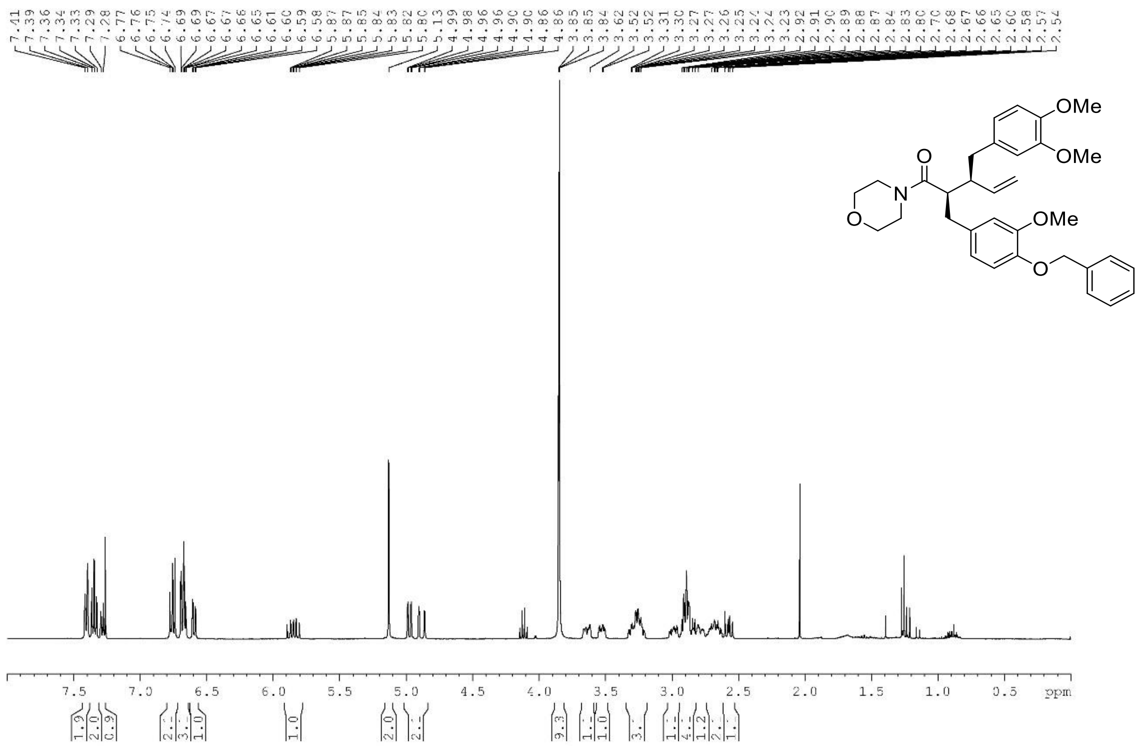

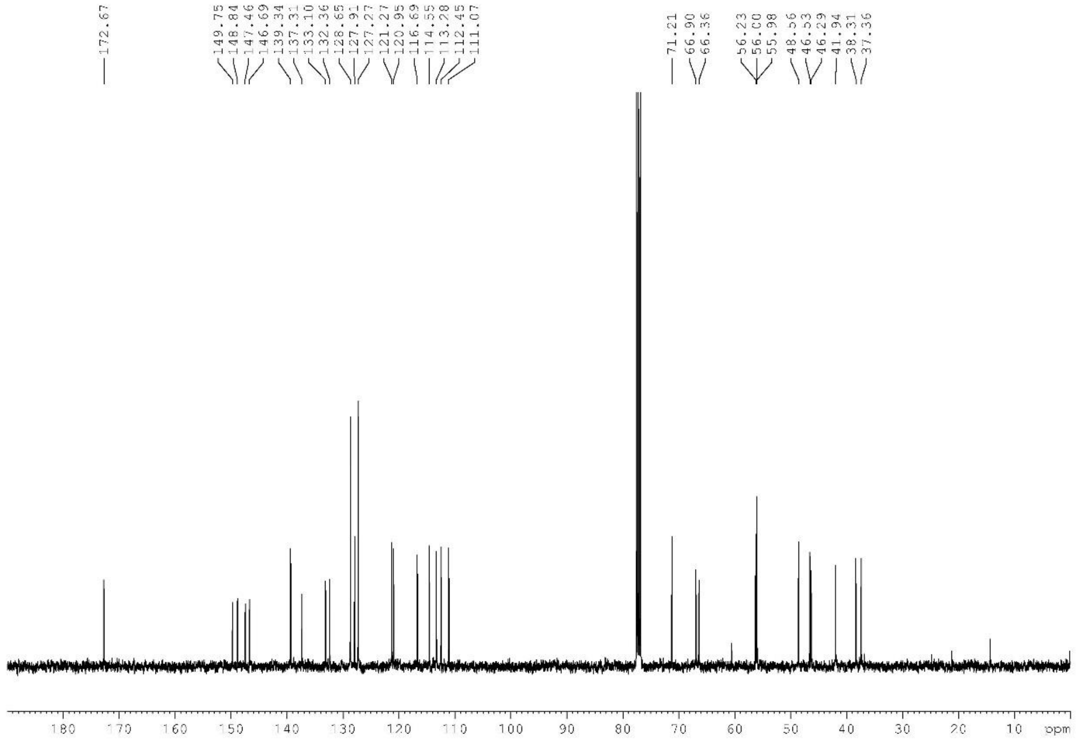

(2*R**,3*S**)-2-(3′,4′-Dimethoxybenzyl)-3-(3″,4″-methylenedioxybenzyl)-1-morpholinopent-4-en-1-one **35ba**

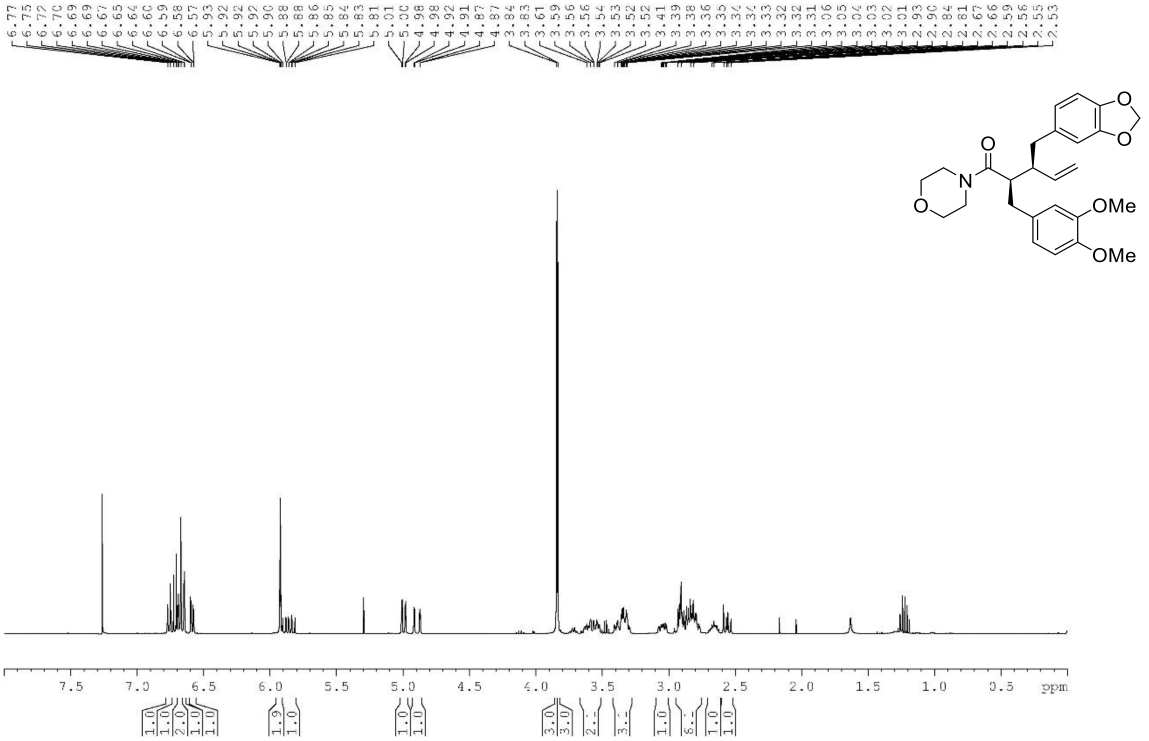

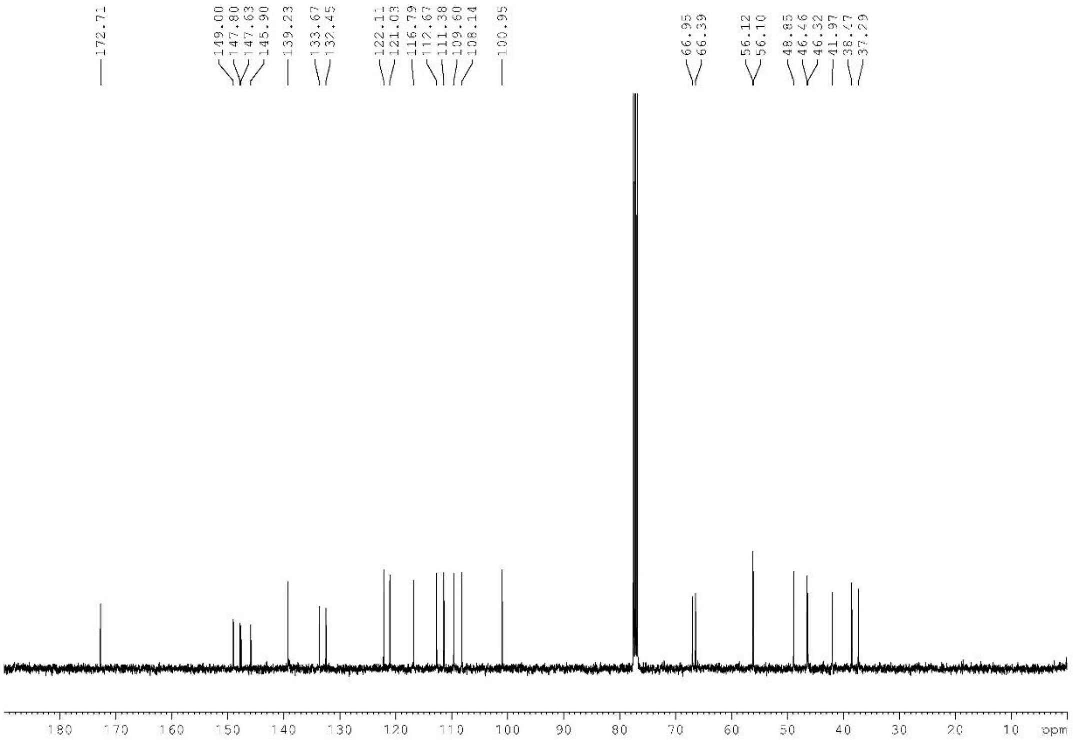

(2*R**,3*S**)-2-(3′,4′-Methylenedioxybenzyl)-3-(3″,4″-methylenedioxybenzyl)-1-morpholinopent-4-en-1-one **35bb**

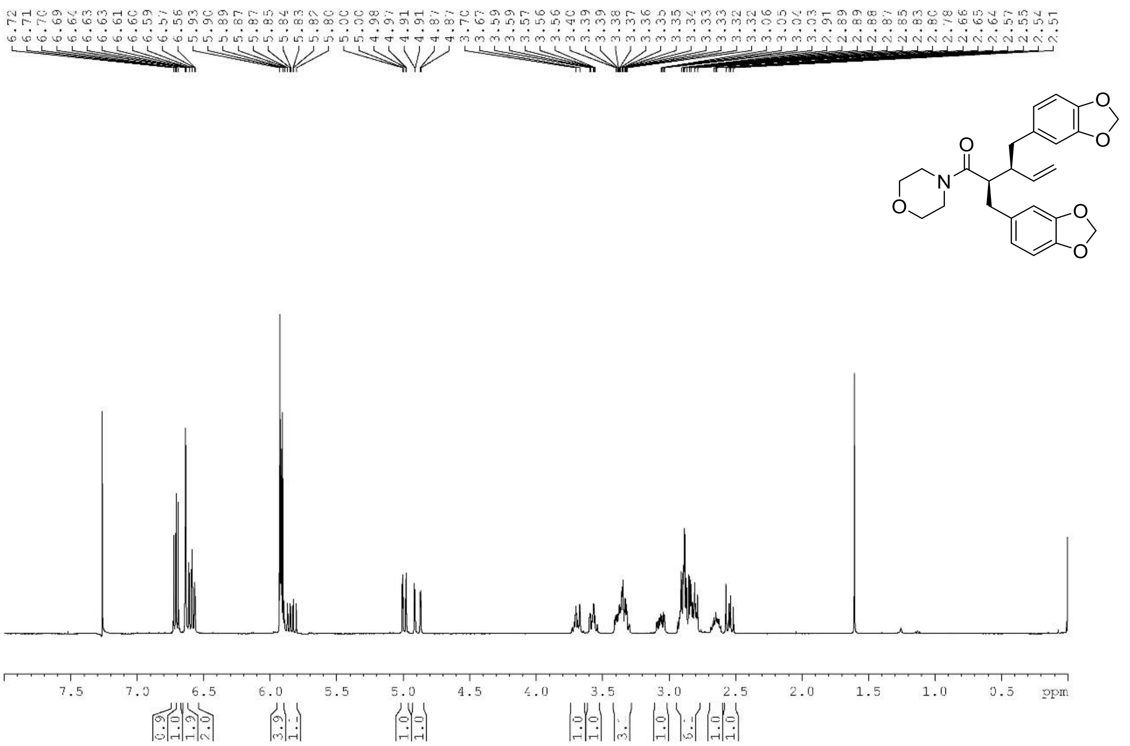

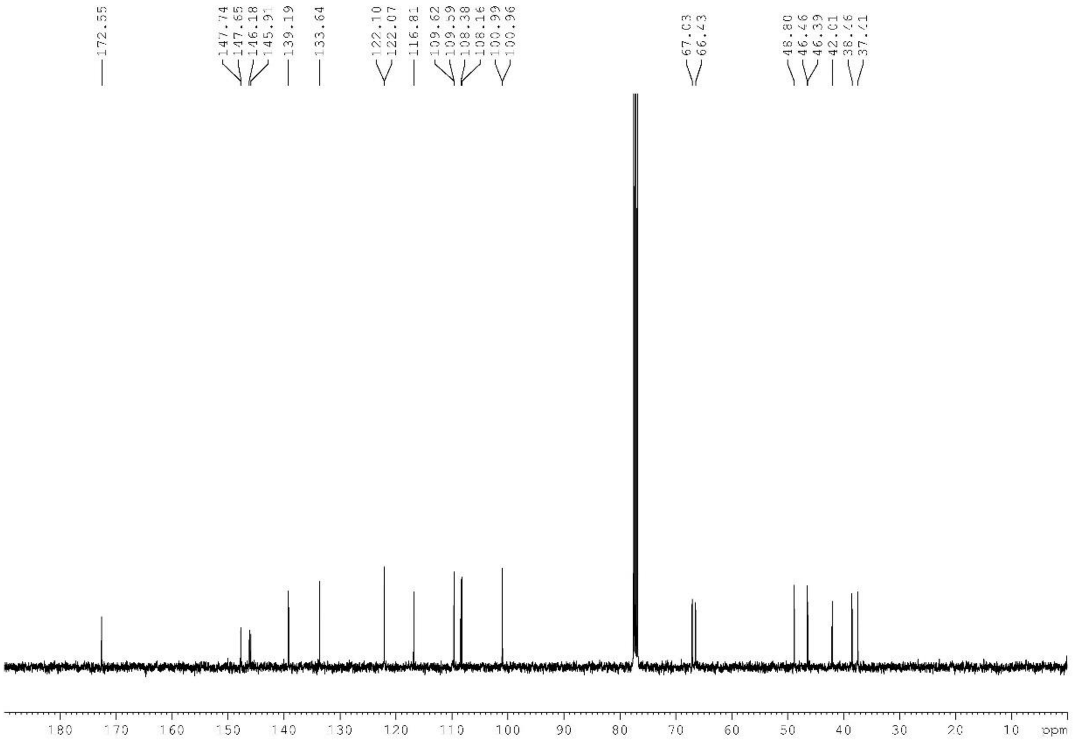

(2*R**,3*S**)-2-(3′,4′,5′-Trimethoxybenzyl)-3-(3″,4″-methylenedioxybenzyl)-1-morpholinopent-4-en-1-one **35bc**

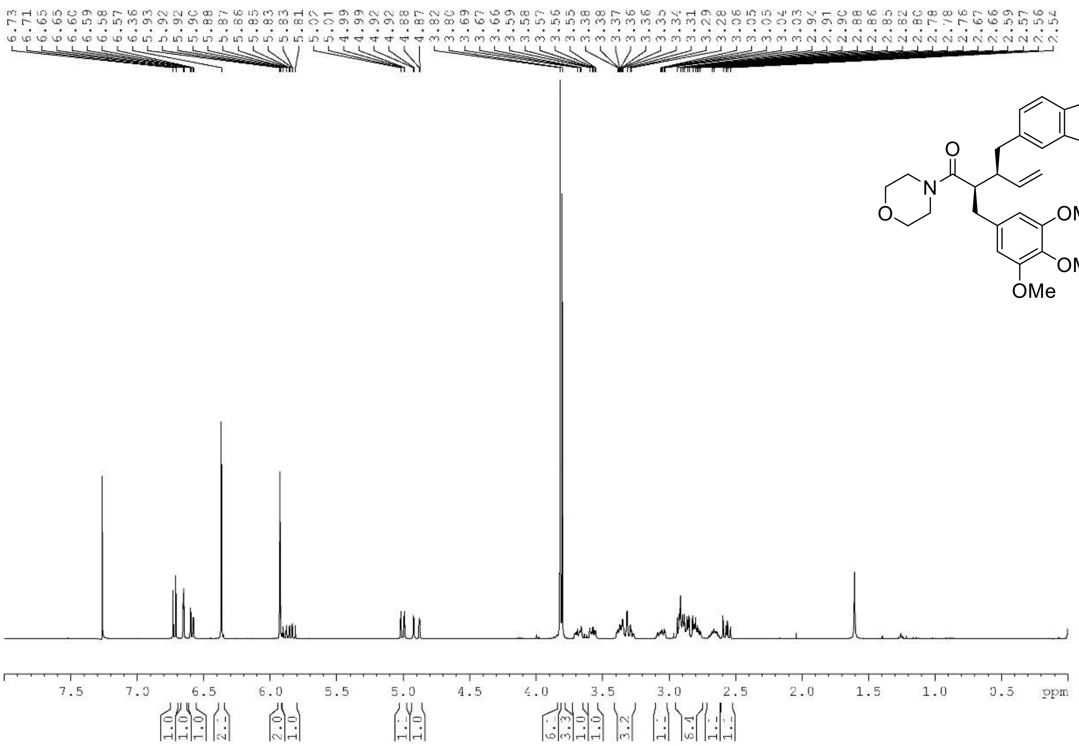

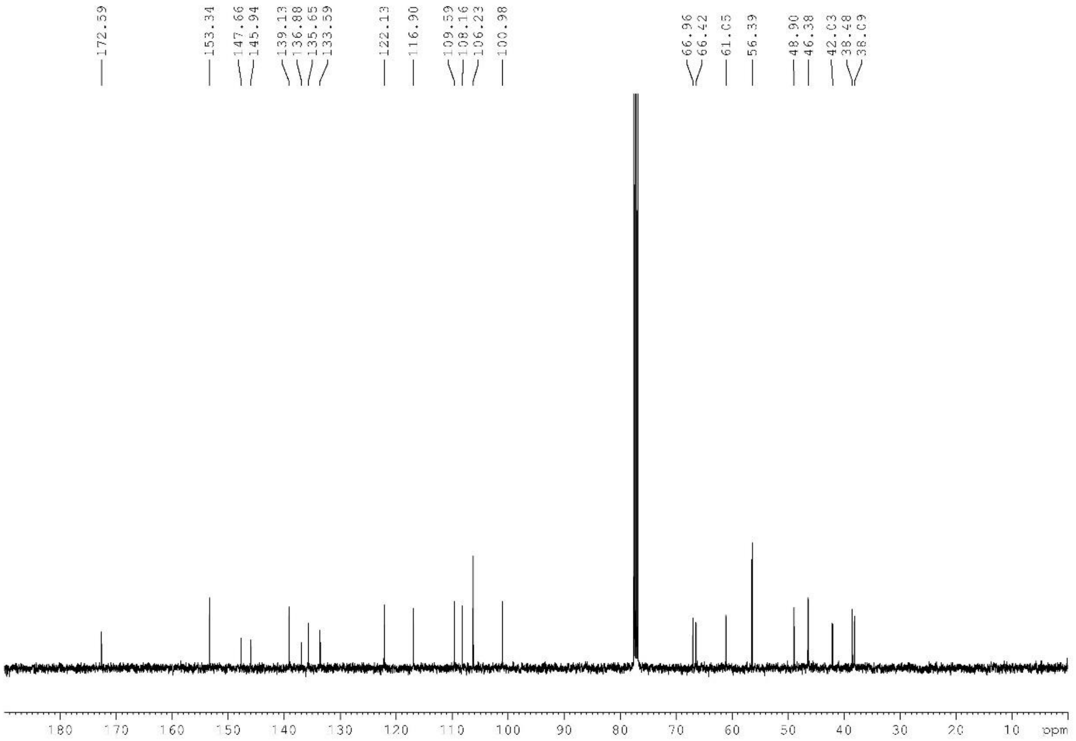

(2*R**,3*S**)-2-(3′-Methoxy-4′-benzyloxybenzyl)-3-(3″,4″-methylenedioxybenzyl)-1-morpholinopent-4-en-1-one **35bd**

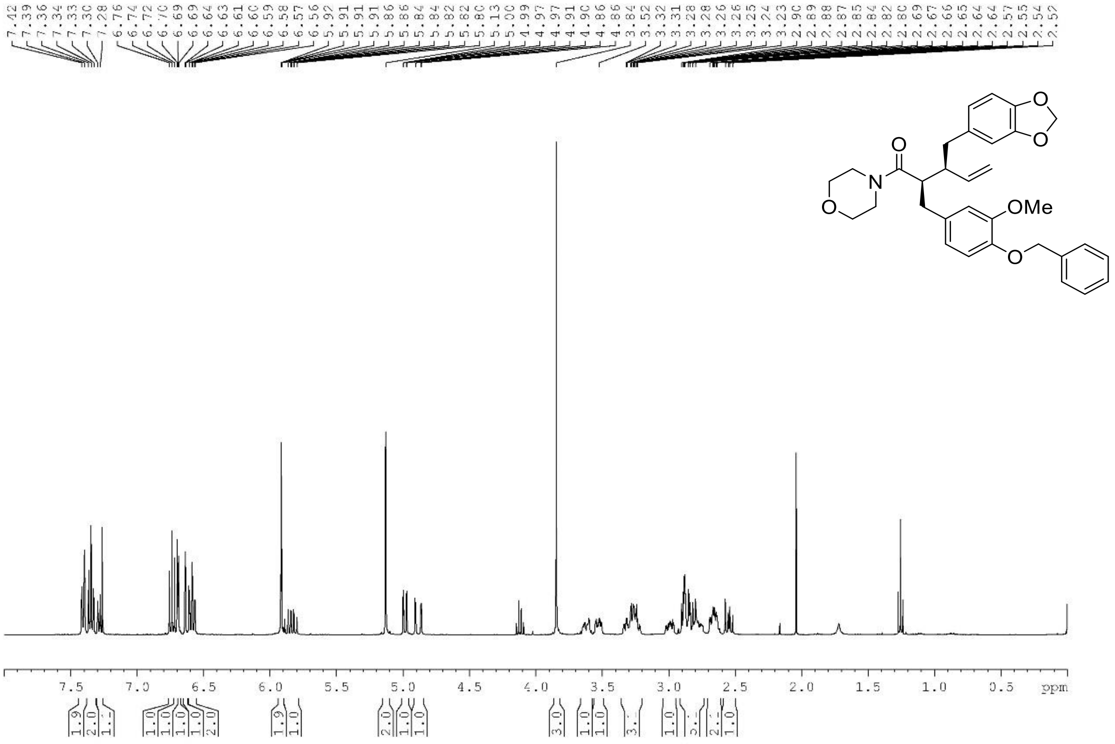

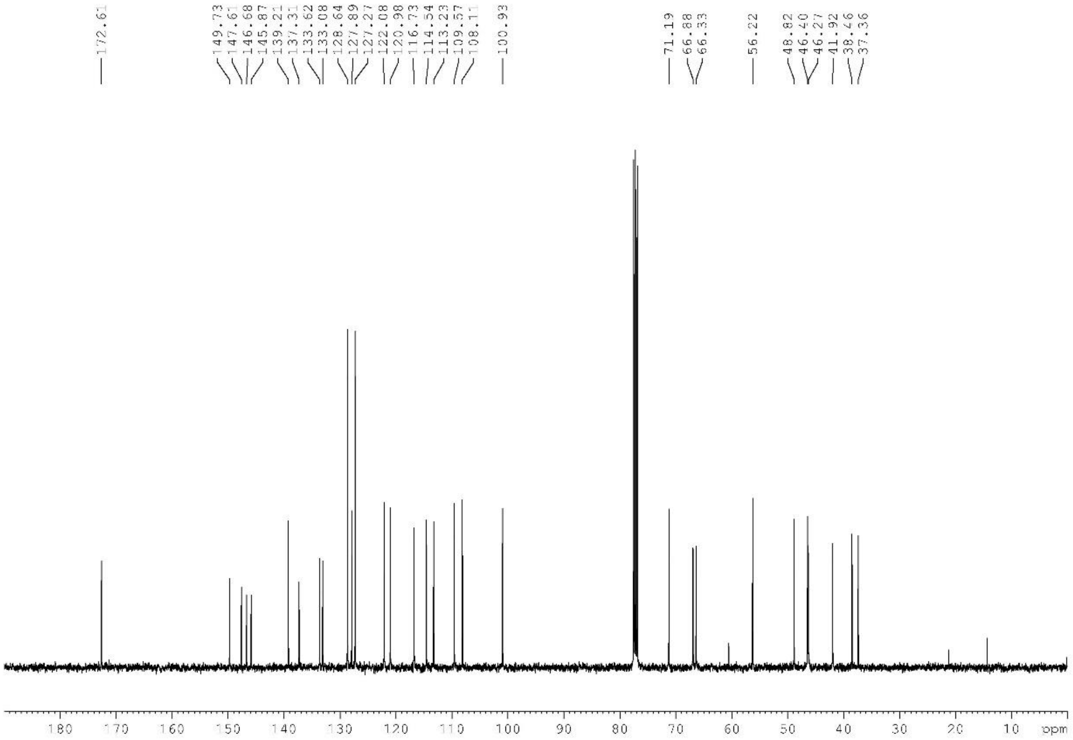

(3*R**,4*R**)-3-(3′,4′-Methylenedioxybenzyl)-4-(3″,4″-dimethoxybenzyl)-5-(hydroxymethyl) dihydrofuran-2(3*H*)-one **4ab**

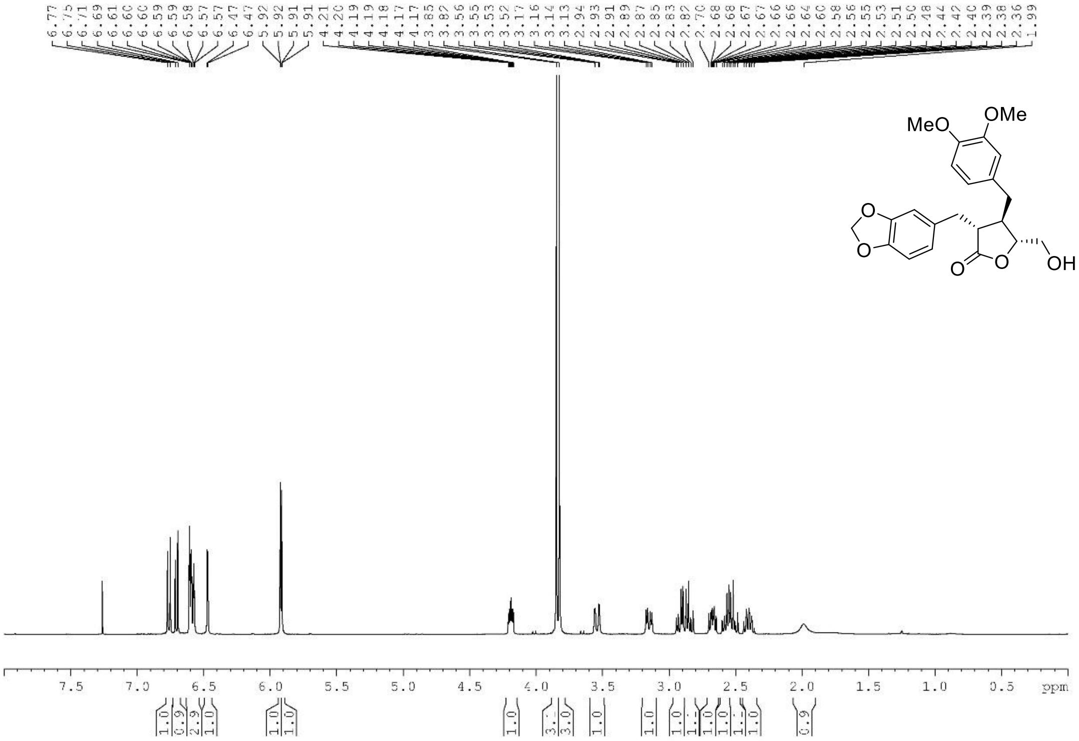

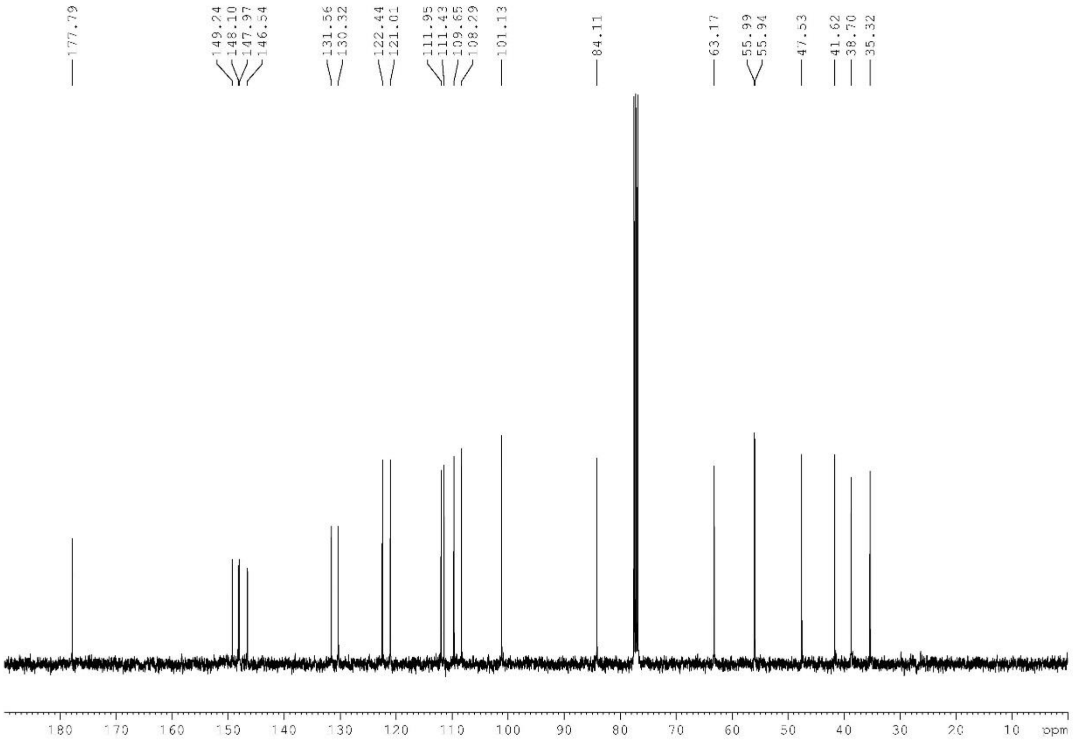

(3*R**,4*R**)-3,4-bis(3′,4′-Dimethoxybenzyl)-5-(hydroxymethyl)dihydrofuran-2(3*H*)-one **4aa**

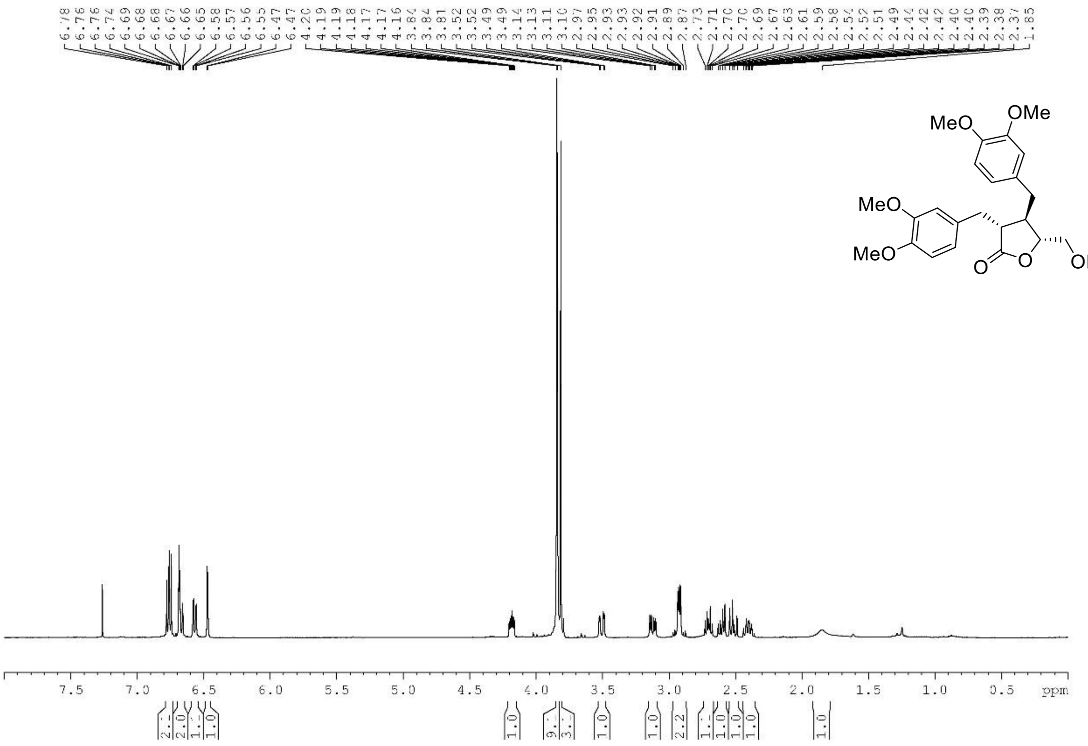

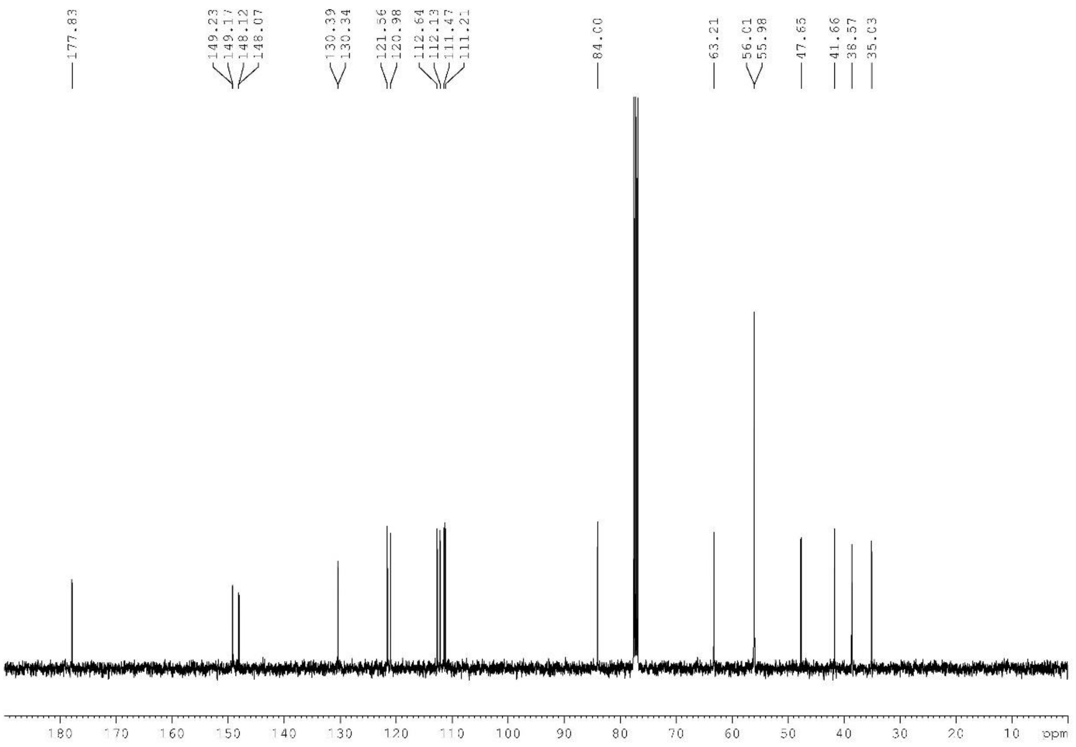

(3*R**,4*R**)-3-(3′,4′,5′-Trimethoxybenzyl)-4-(3″,4″-dimethoxybenzyl)-5-(hydroxymethyl) dihydrofuran-2(3*H*)-one **4ac**

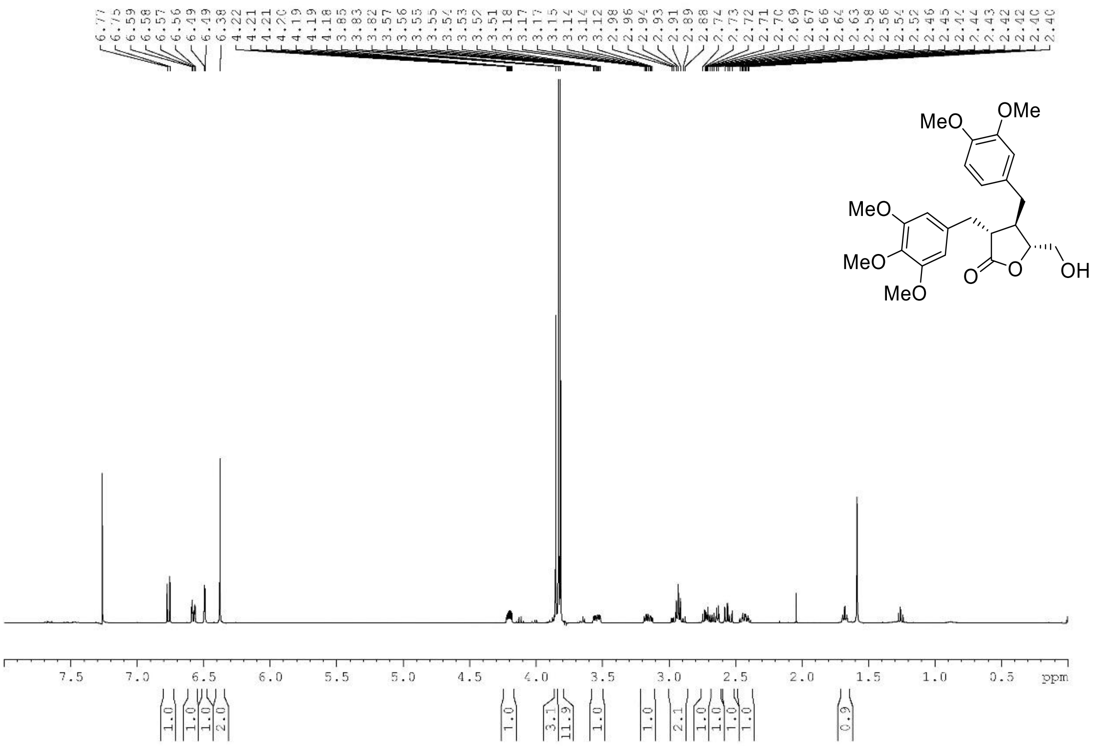

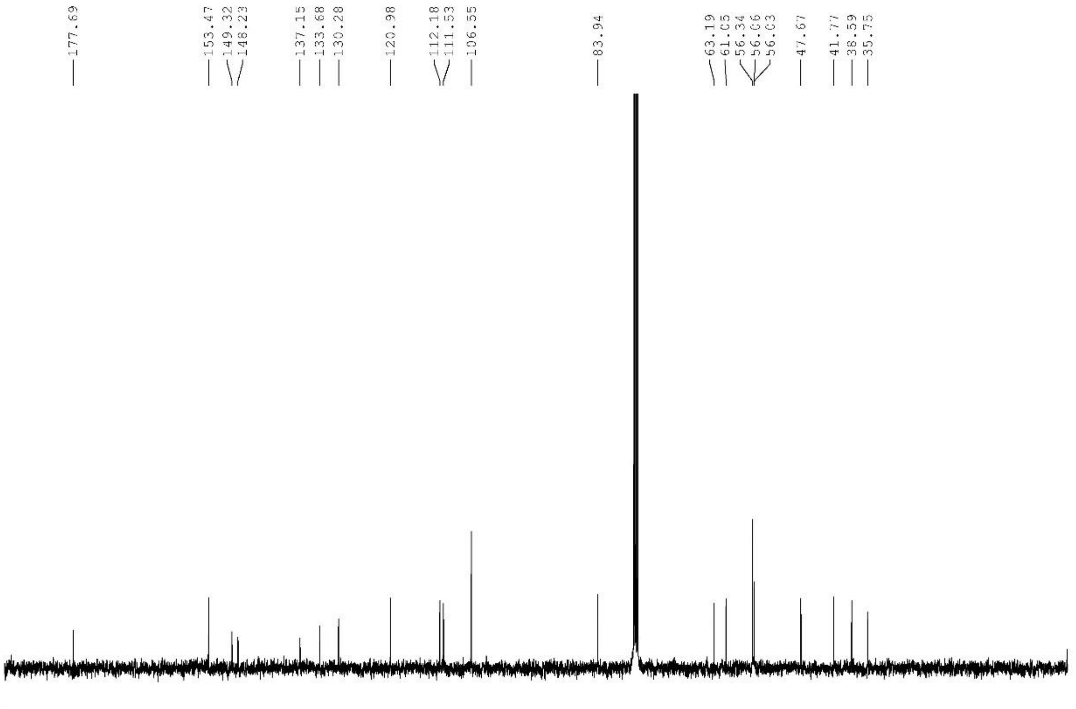

(3*R**,4*R**)-3-(3′-Methoxy-4′-benzyloxybenzyl)-4-(3″,4″-dimethoxybenzyl)-5-(hydroxymethyl) dihydrofuran-2(3*H*)-one **4ad**

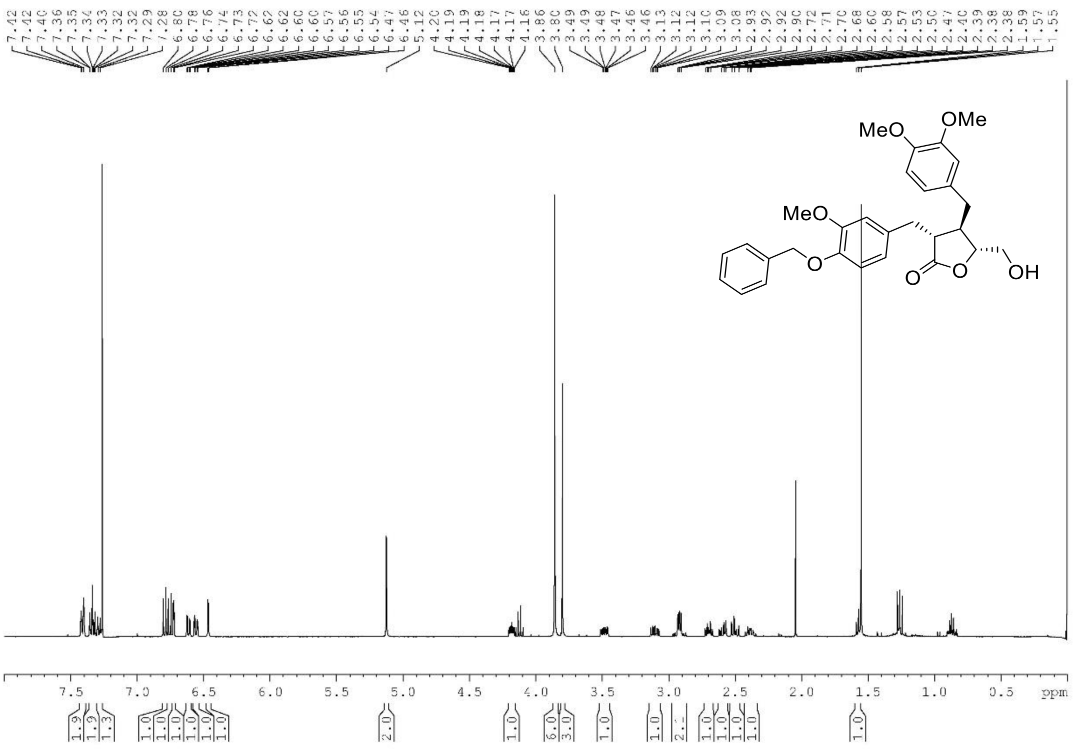

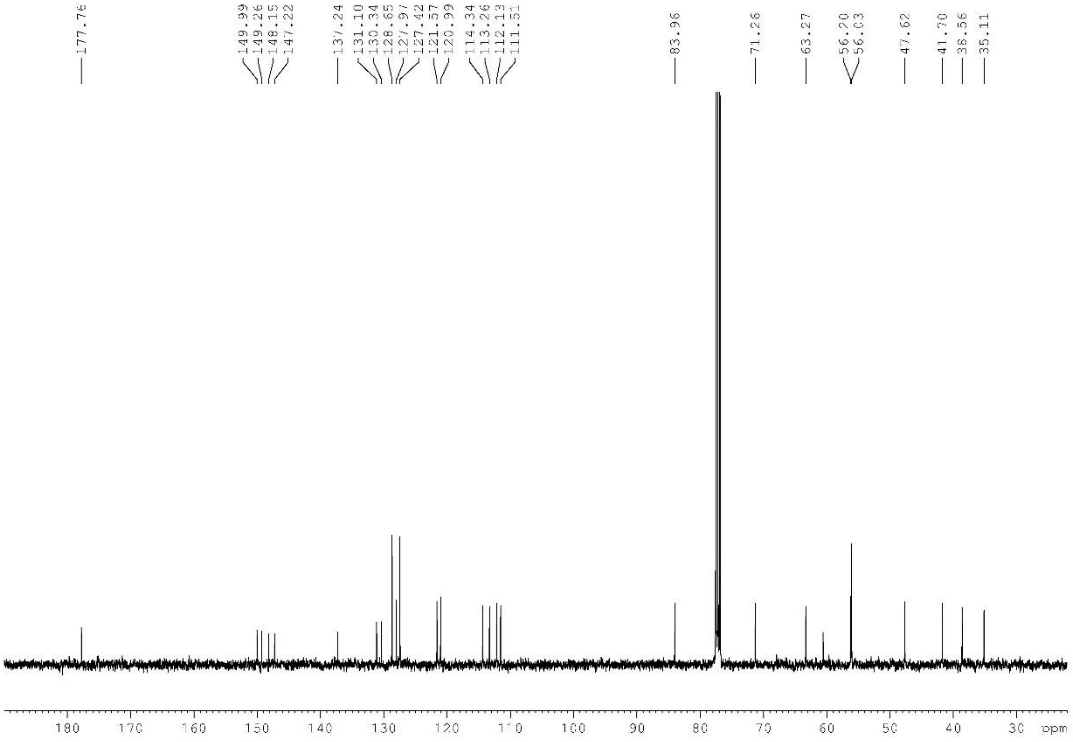

(3*R**,4*R**,5*S**)-4-(3″,4″-Dimethoxybenzyl)-3-(4′-hydroxy-3′-methoxybenzyl)-5-(hydroxymethyl)dihydrofuran-2(3H)-one **4ae**

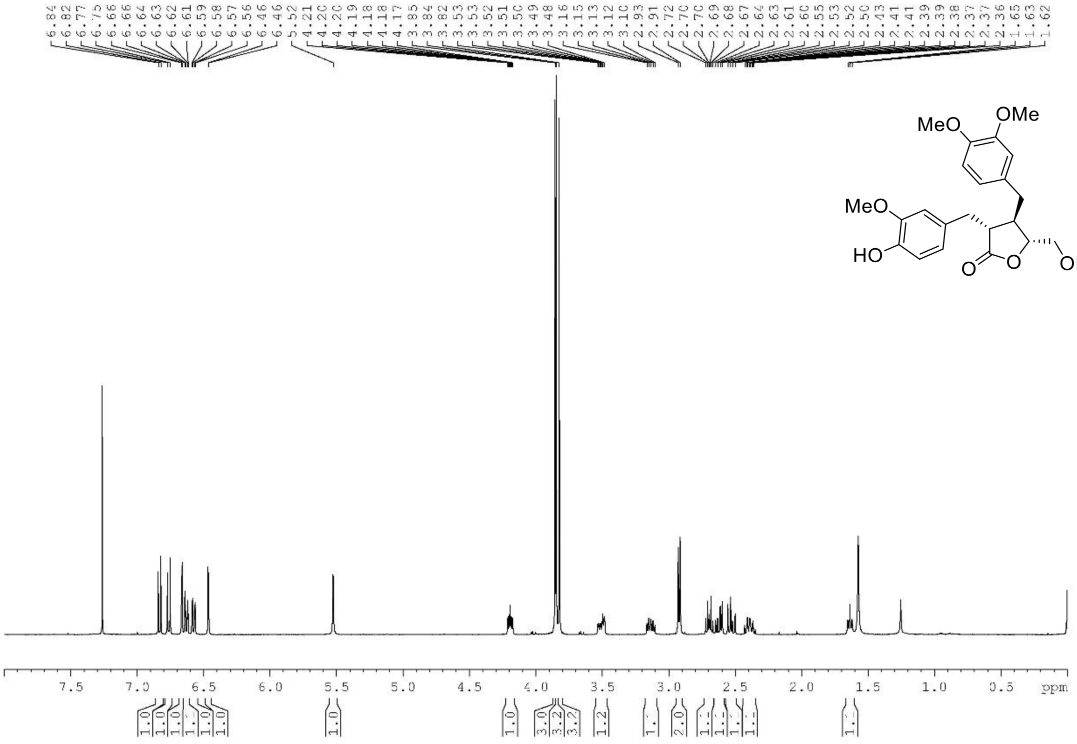

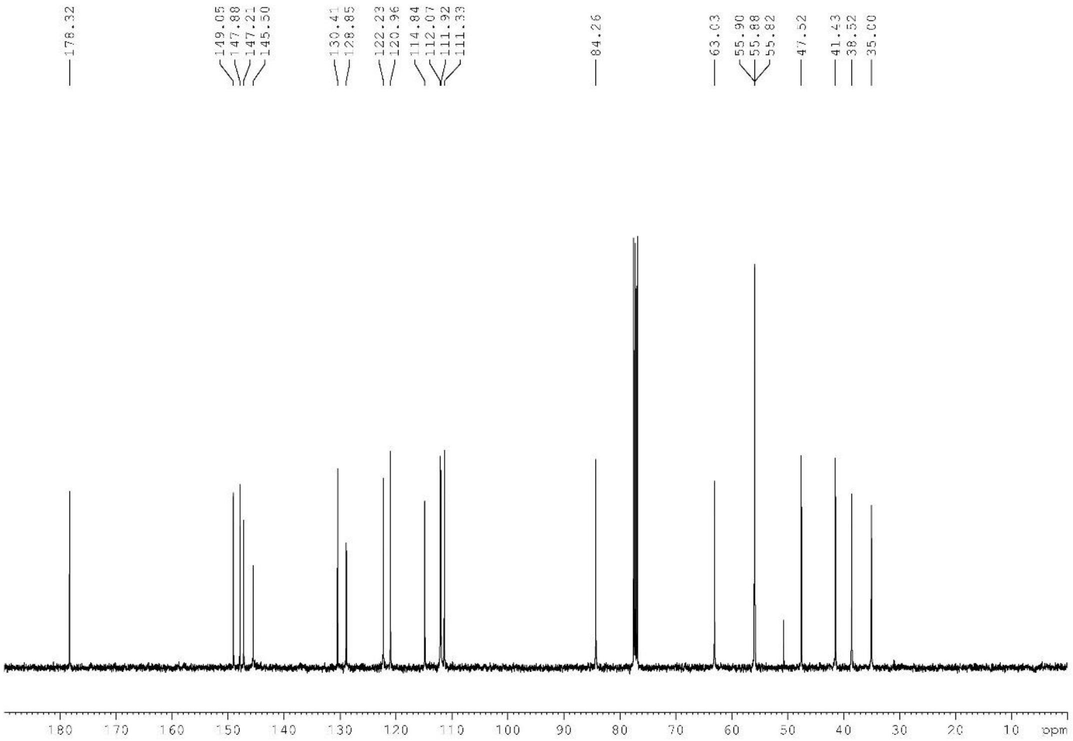

(3*R**,4*R**)-3,4-bis(3′,4′-Methylenedioxybenzyl)-5-(hydroxymethyl)dihydrofuran-2(3*H*)-one **4bb**

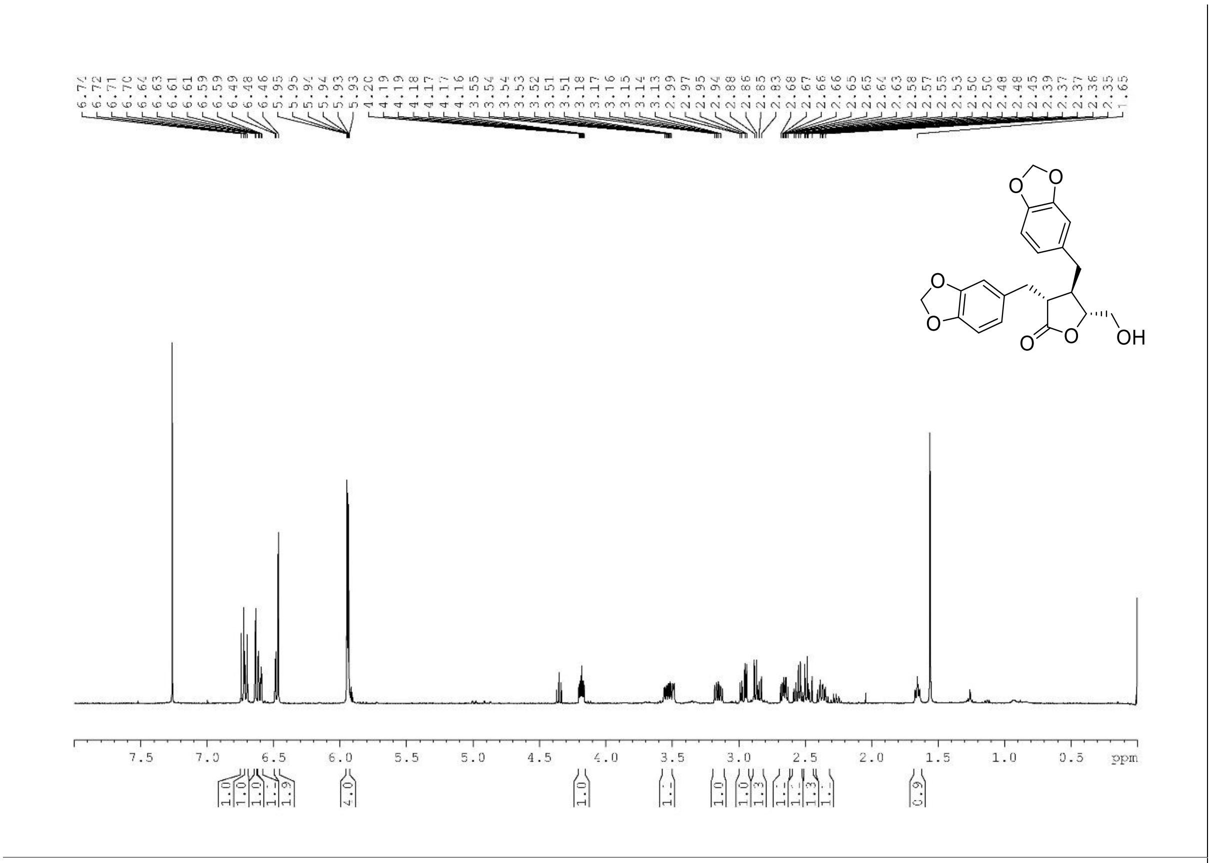

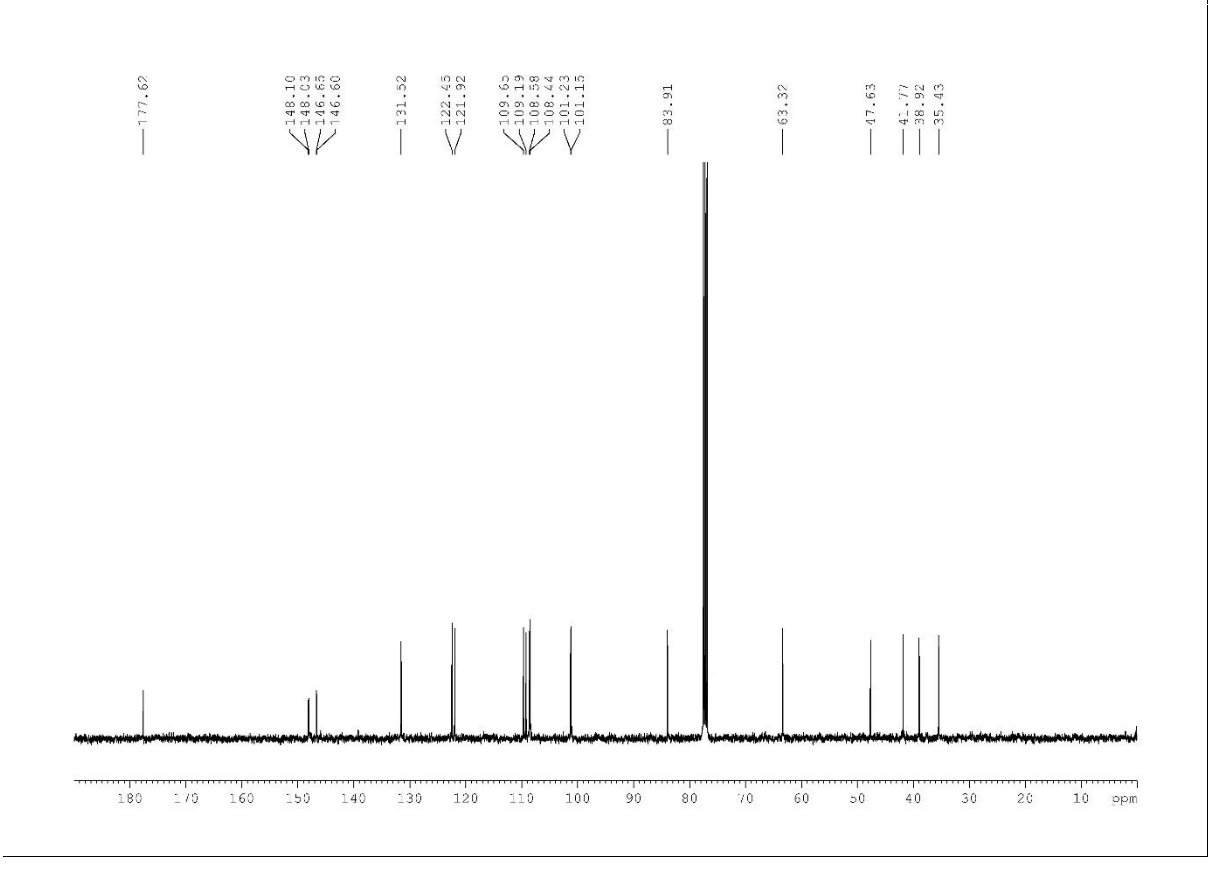

(3*R**,4*R**)-3,4-bis(3′,4′-Methylenedioxybenzyl)-5-(hydroxymethyl)dihydrofuran-2(3*H*)-one (*epi*-**4bb**)

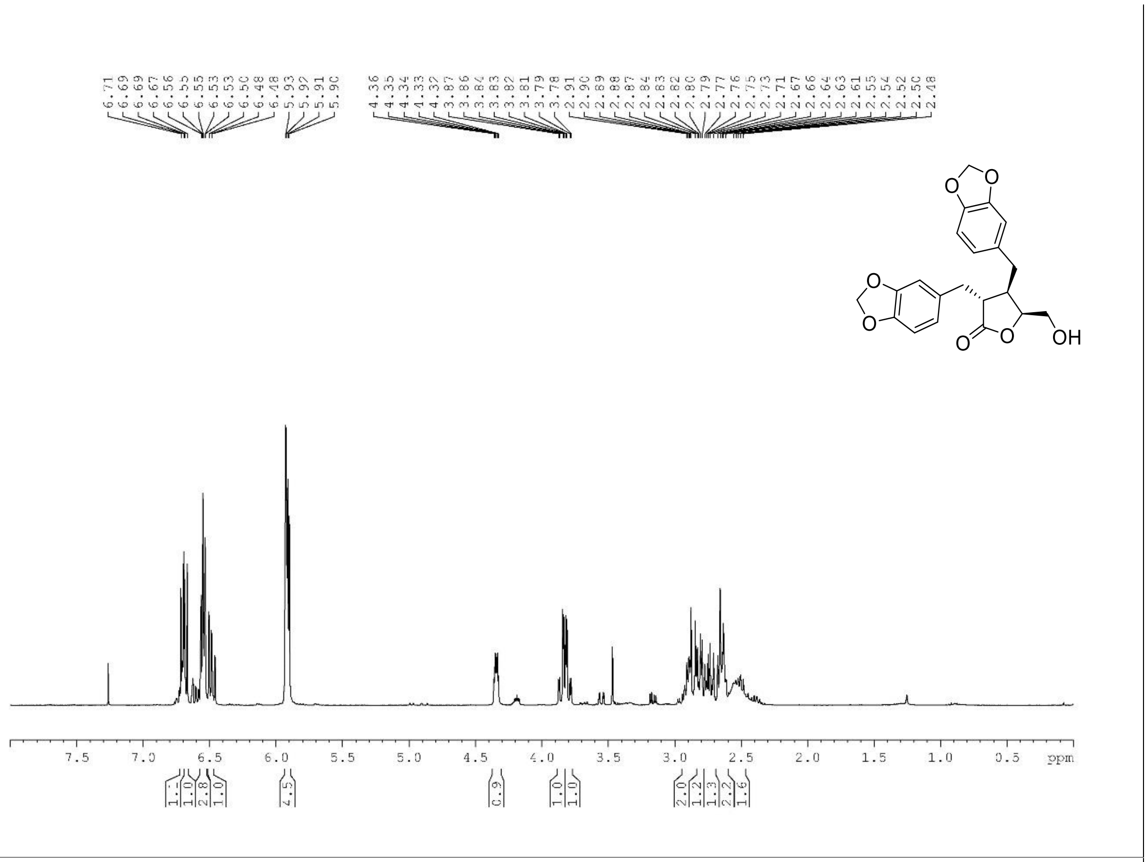

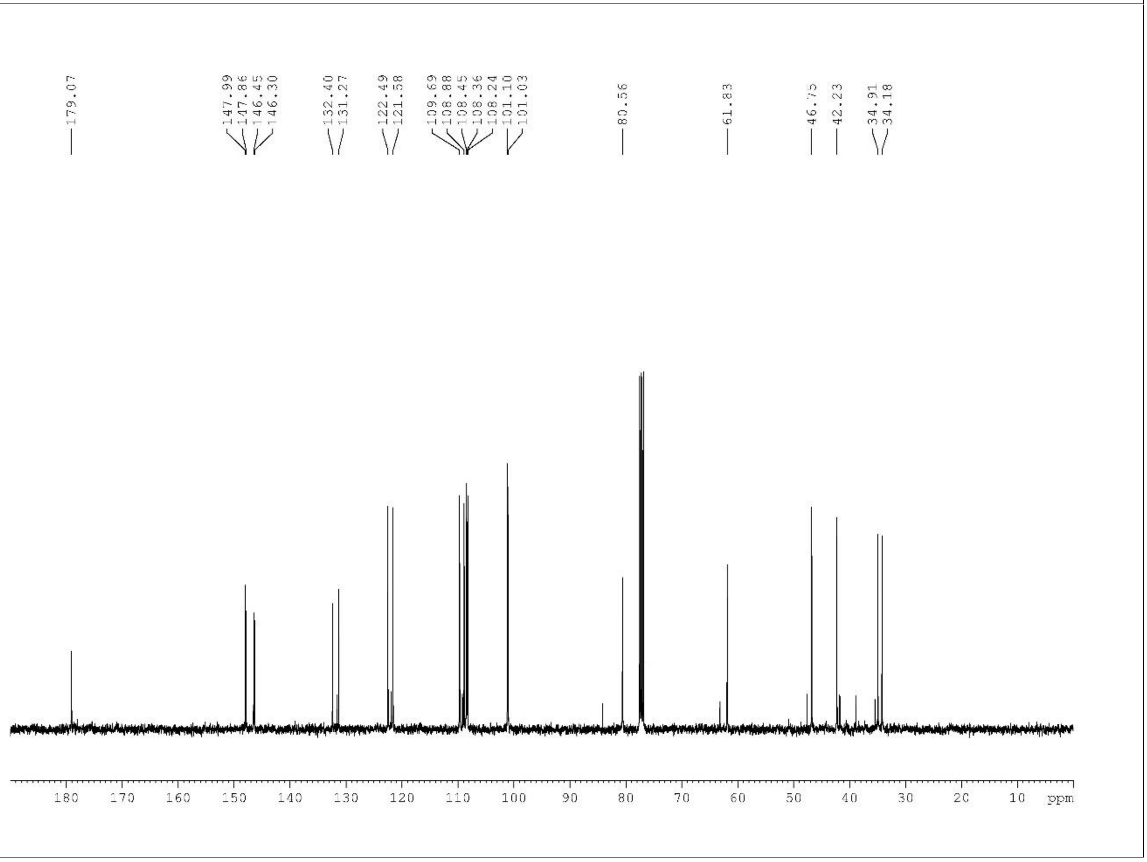

(3*R**,4*R**)-3-(3′,4′-Dimethoxybenzyl)-4-(3″,4″-methylenedioxybenzyl)-5-(hydroxymethyl)dihydrofuran-2(3*H*)-one **4ba**

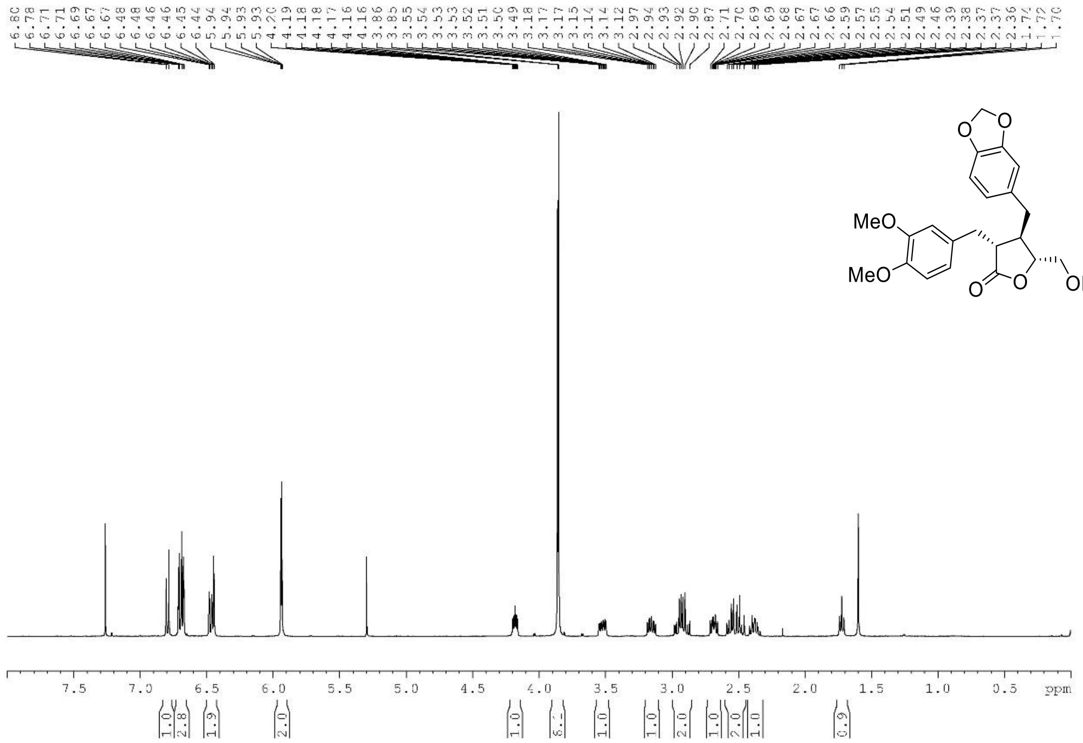

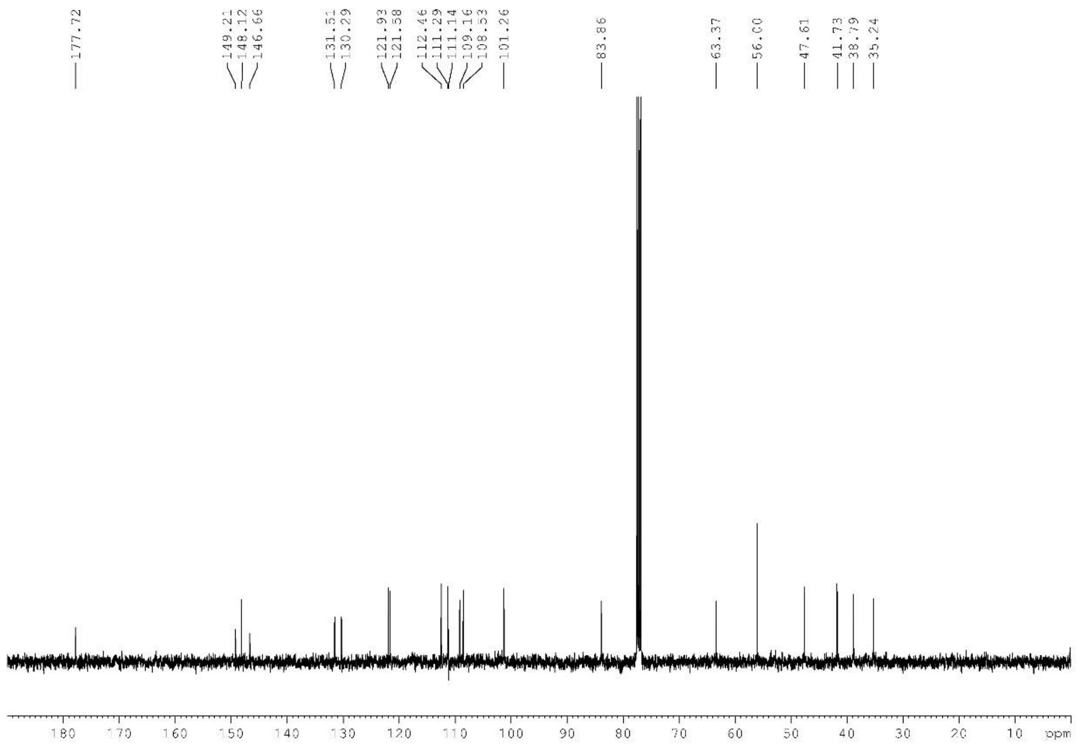

(3*R**,4*R**)-3-(3′,4′,5′-Trimethoxybenzyl)-4-(3″,4″-methylenedioxybenzyl)-5-(hydroxymethyl) dihydrofuran-2(3*H*)-one **4bc**

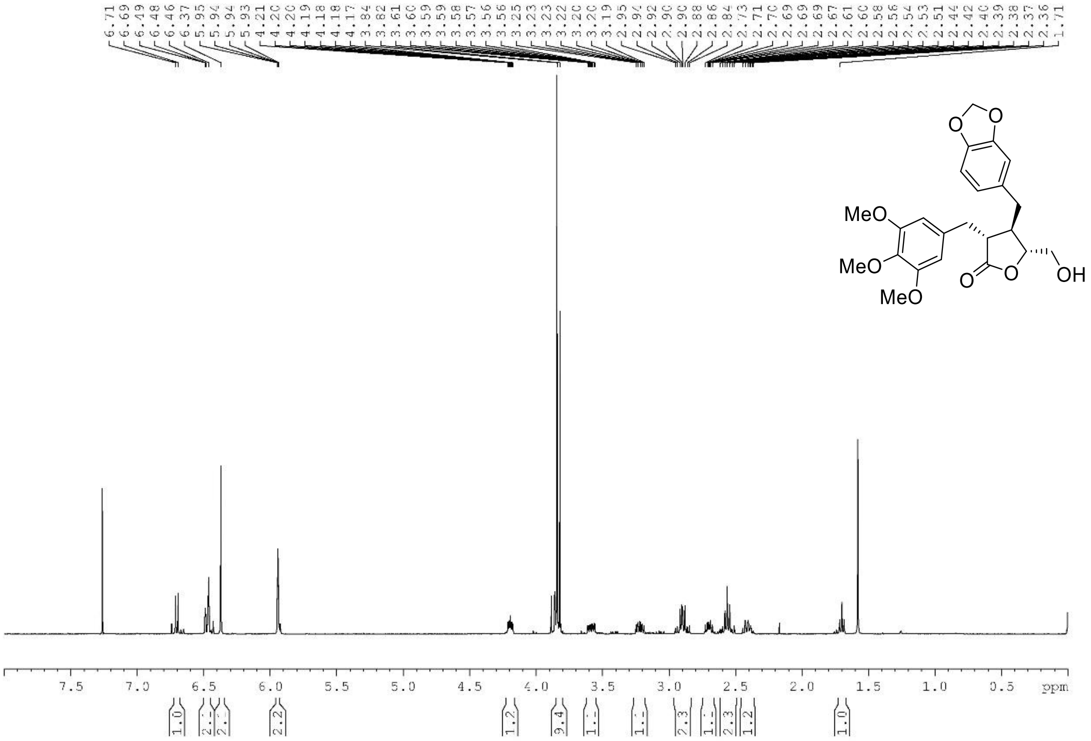

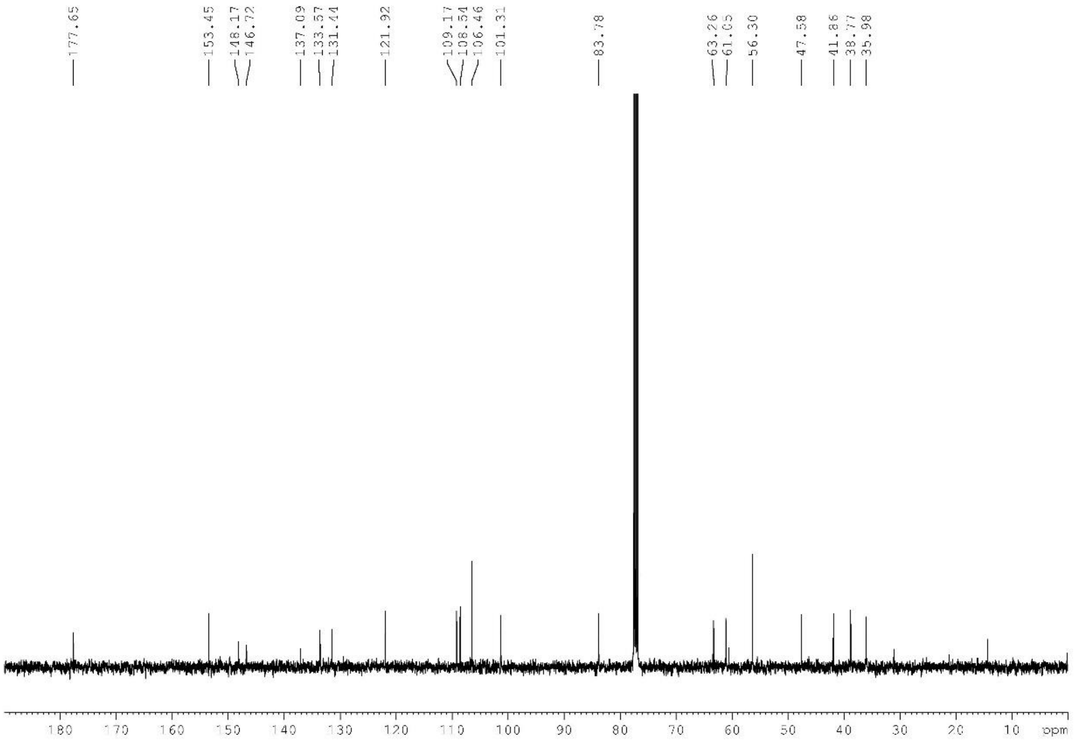

(3*R**,4*R**)-3-(3′-Methoxy-4′-benzyloxybenzyl)-4-(3″,4″-methylenedioxybenzyl)-5-(hydroxymethyl) dihydrofuran-2(3*H*)-one **4bd**

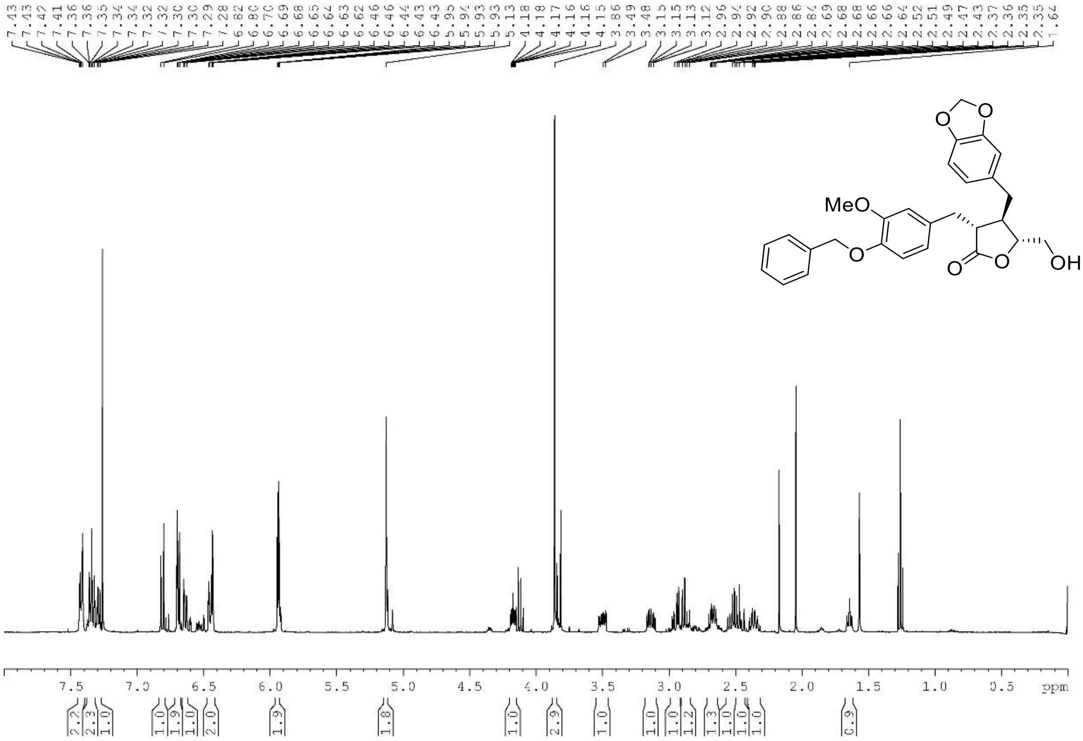

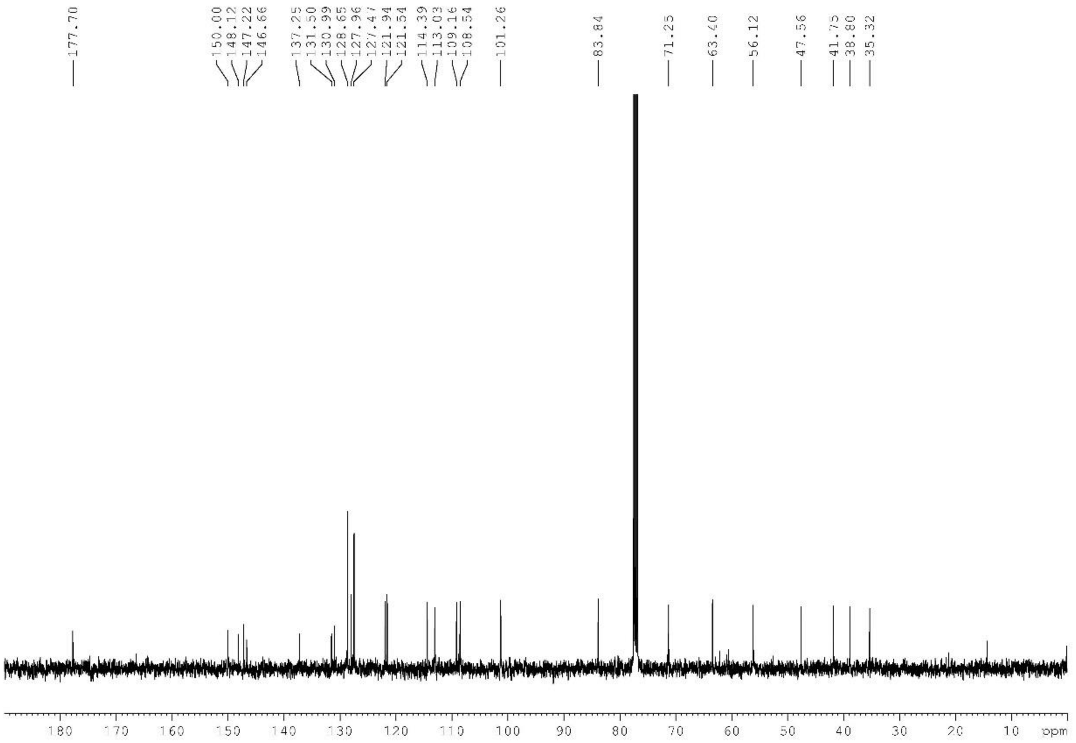

(3*R**,4*R**,5*S**)-4-(3″,4″-Methylenedioxybenzyl)-3-(4′-hydroxy-3′-methoxybenzyl)-5-(hydroxymethyl)dihydrofuran-2(3H)-one **4be**

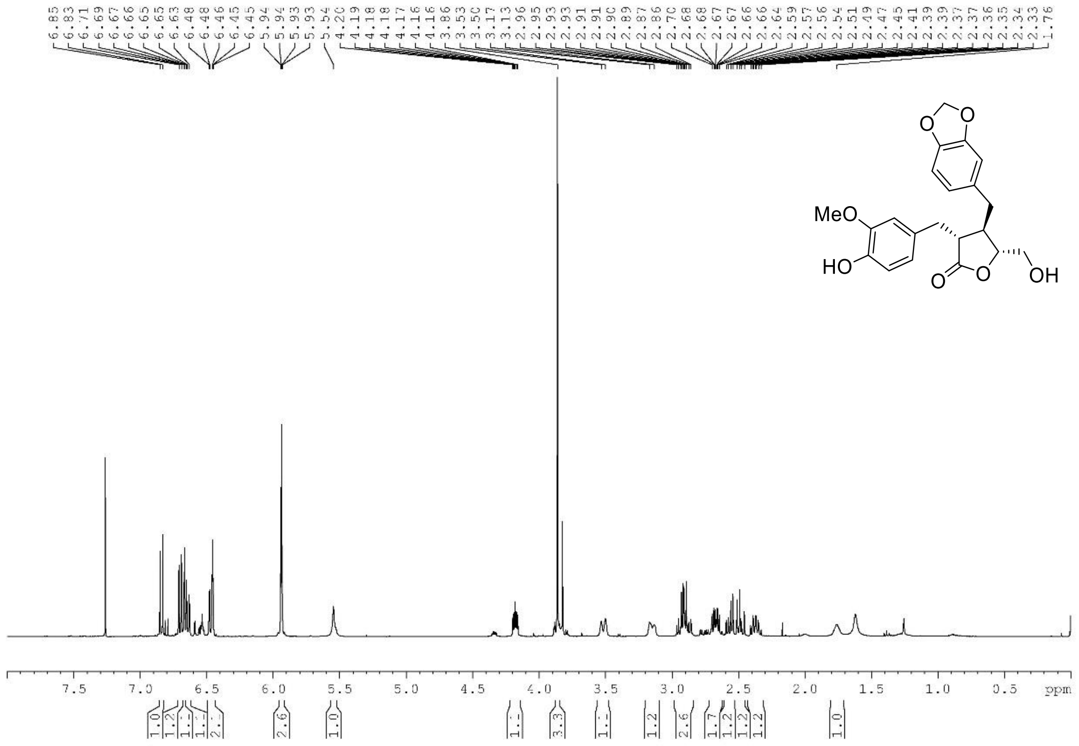

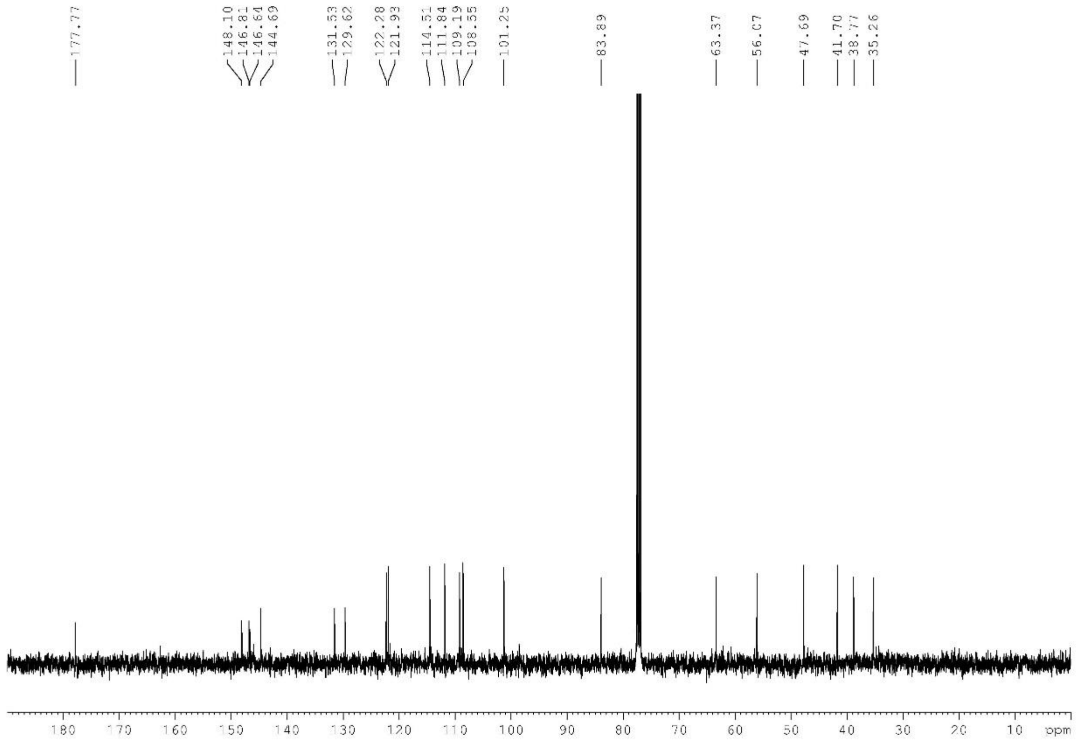

(±)-Bursehernin **1ab**

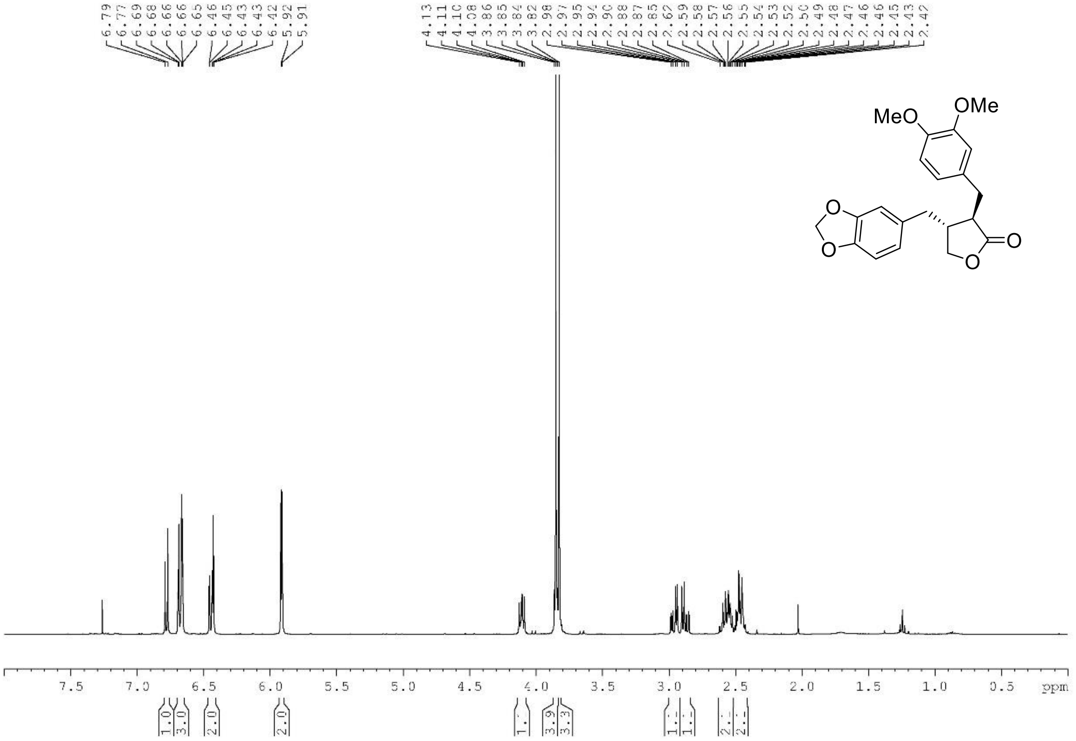

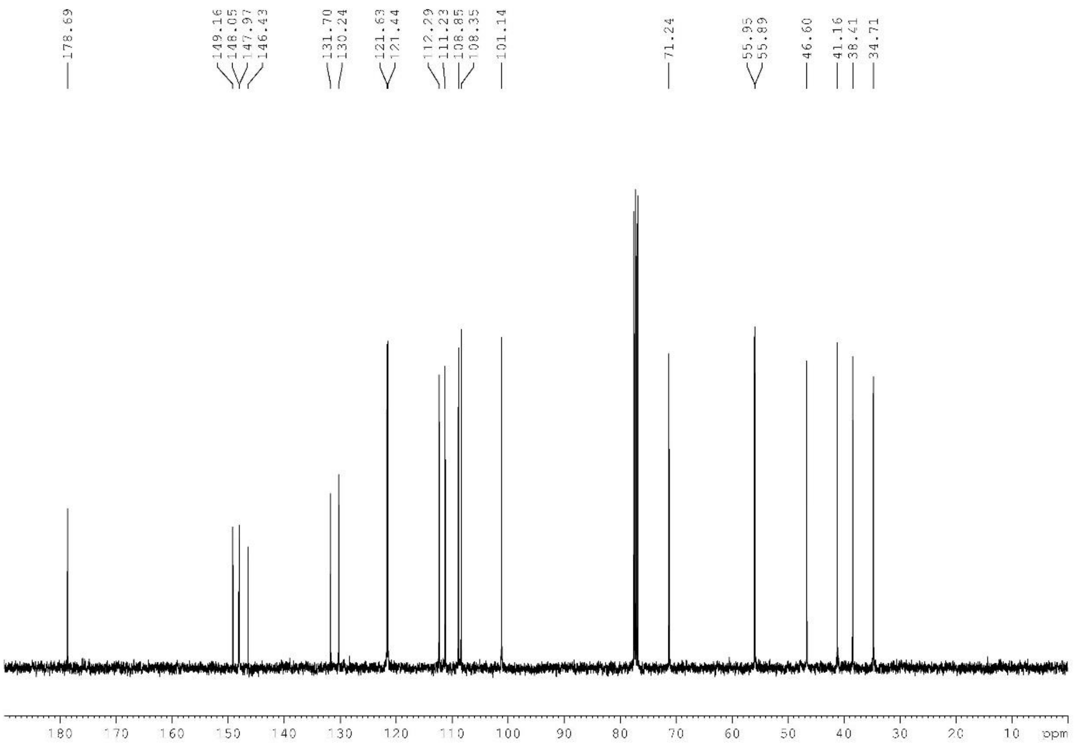

(±)-Arcitin **1aa**

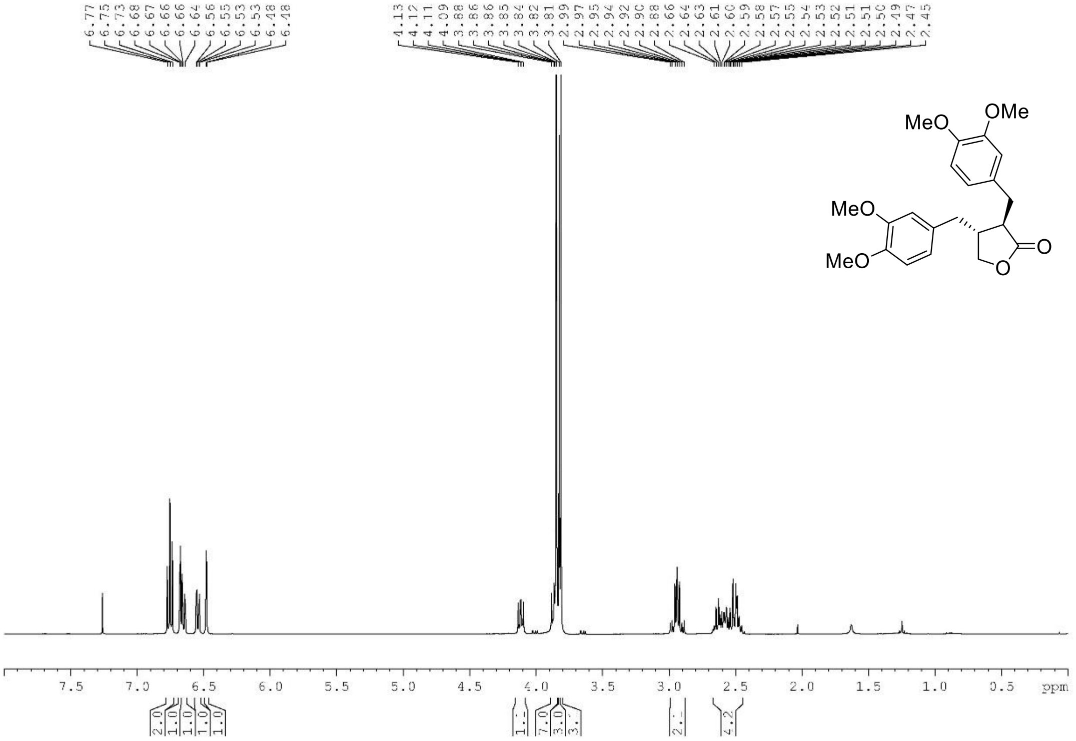
6.77 6.75 6.73 6.68 6.67 6.66 6.66 6.64 6.56 6.55 6.53 6.53 6.48 6.48
4.13 4.12 4.11 4.09 3.88 3.86 3.86 3.85 3.84 3.82 3.81 2.99 2.97 2.95 2.94 2.92 2.90 2.88 2.66 2.64 2.63 2.61 2.60 2.59 2.58 2.57 2.55 2.54 2.53 2.52 2.51 2.51 2.50 2.49 2.47 2.45
MeO
OMe
MeO
MeO
O
O
7.5 7.0 6.5 6.0 5.5 5.0 4.5 4.0 3.5 3.0 2.5 2.0 1.5 1.0 0.5 ppm
2.0 1.0 1.0 1.0 1.0
7.0 3.0
4.2

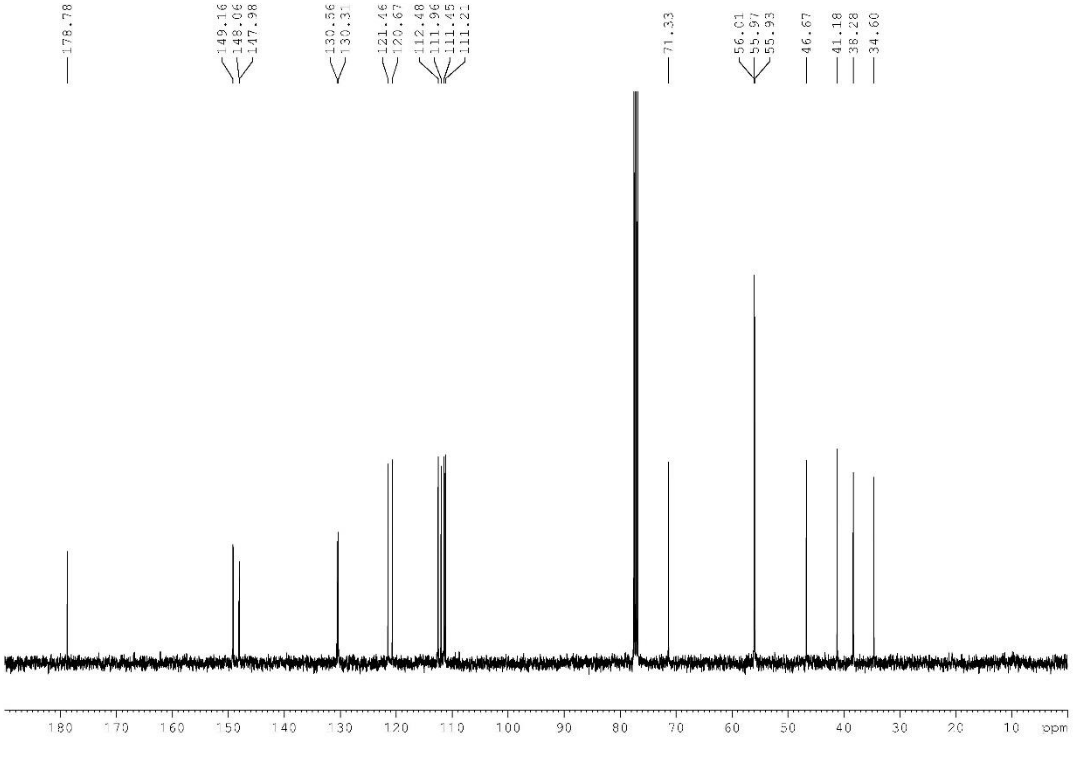
178.78
149.16 148.06 147.98
130.56 130.31
121.46 120.67
112.48 111.96 111.45 111.21
71.33
56.01 55.97 55.93
46.67
41.18
38.28
34.60
180 170 160 150 140 130 120 110 100 90 80 70 60 50 40 30 20 10 ppm

(3*R**,4*R**)-3-(3″,4″-Dimethoxybenzyl)-4-(3′,4′,5′-trimethoxybenzyl)dihydrofuran-2(3H)-one **1ac**

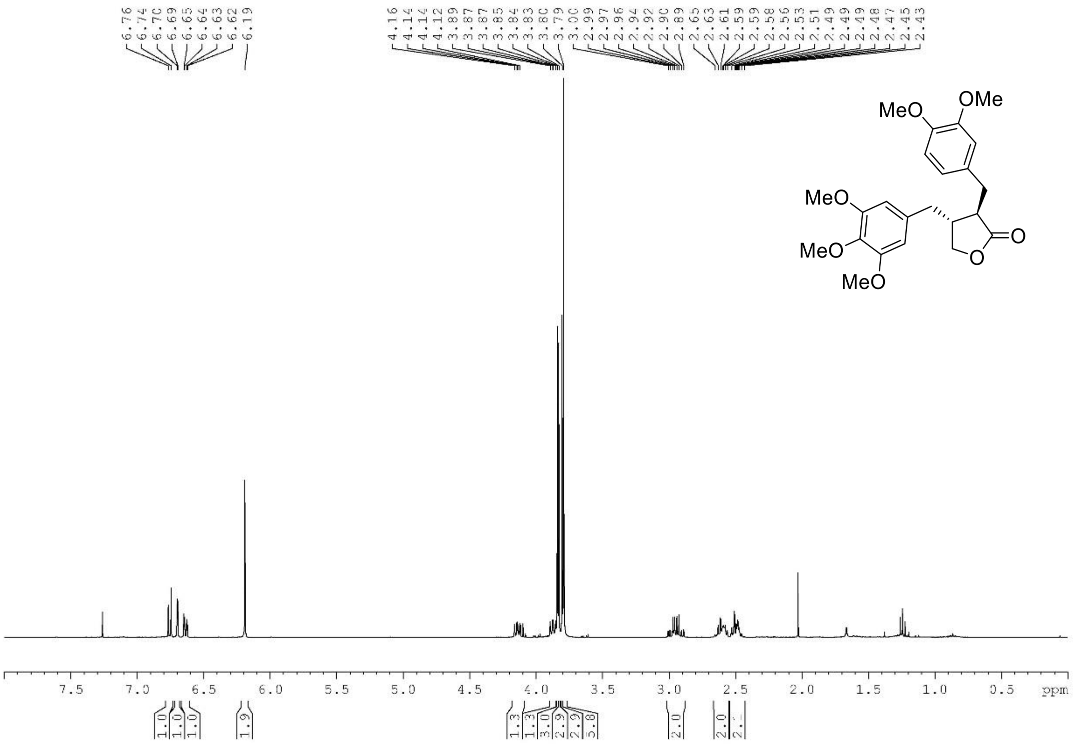

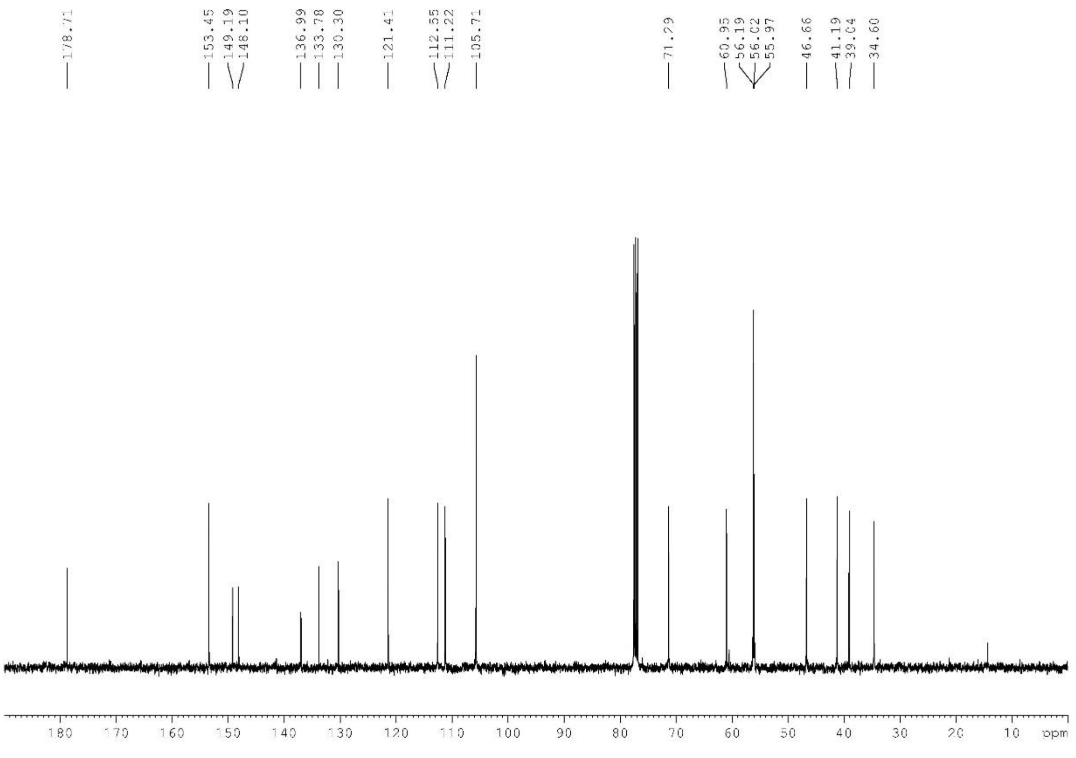

(±)-4′-*O*-Benzyl buplerol **1ad**

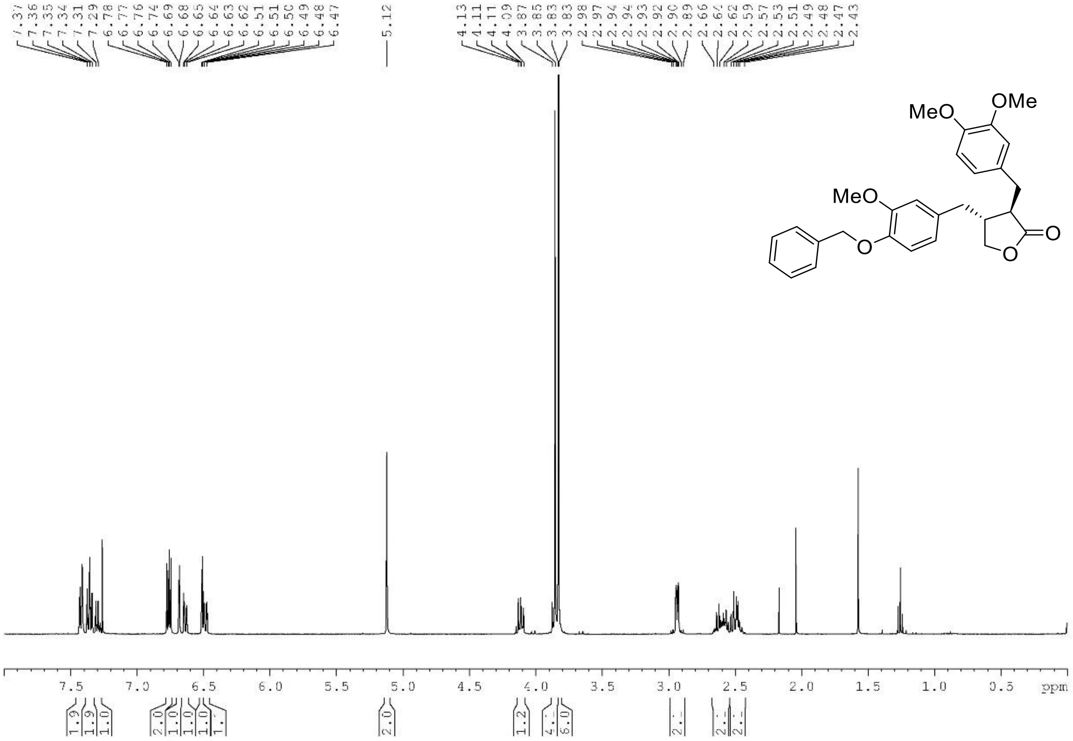

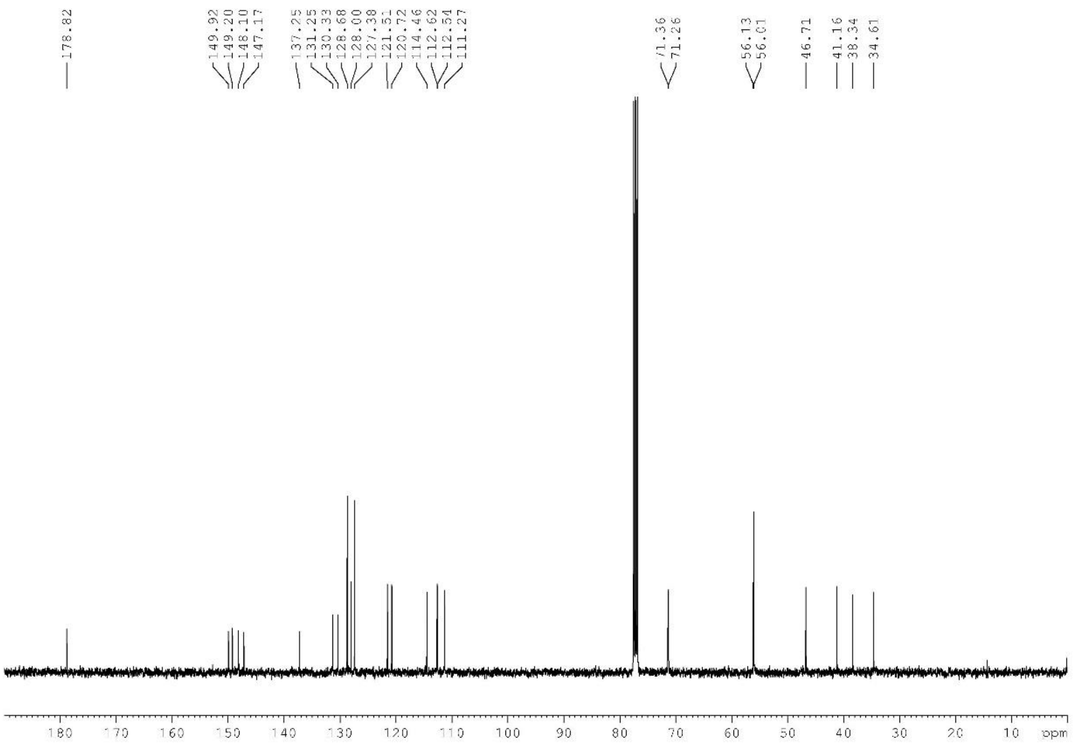

(±)-Buplerol **1ae**

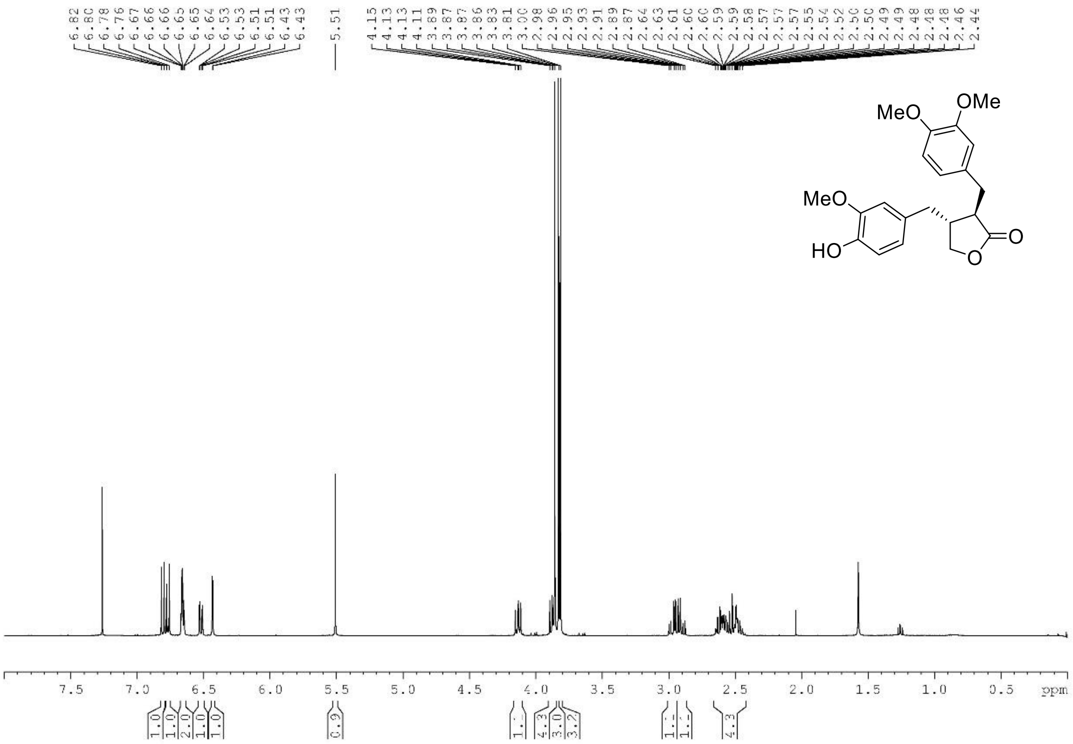

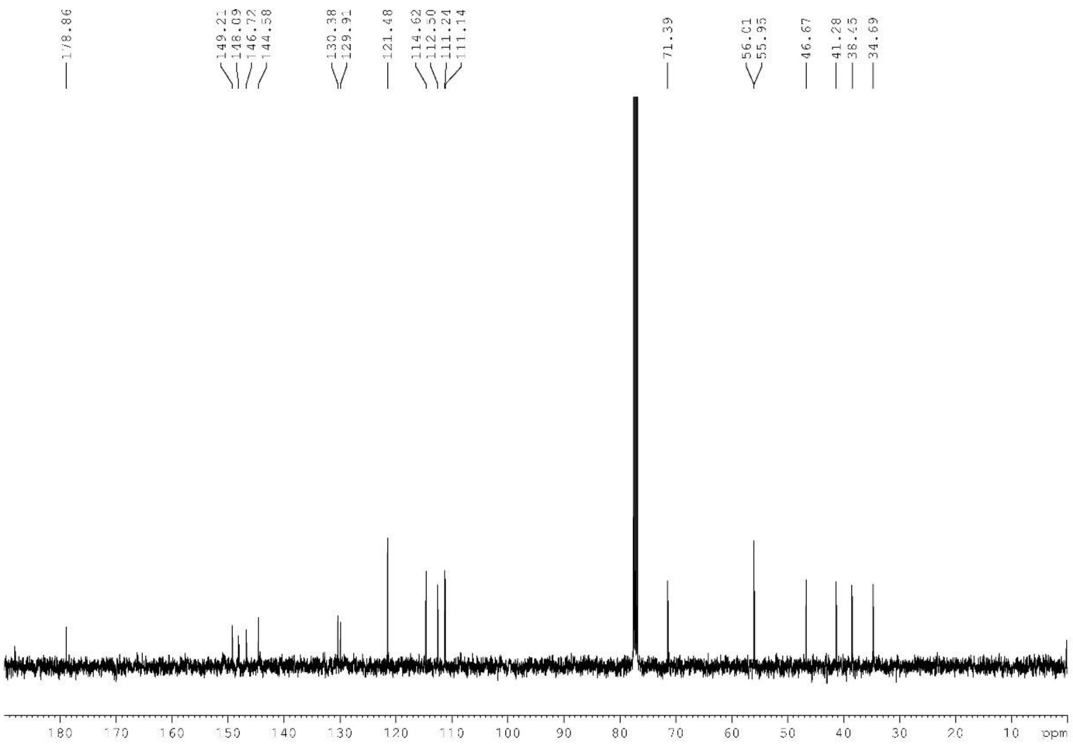

(±)-Hinokinin **1bb**

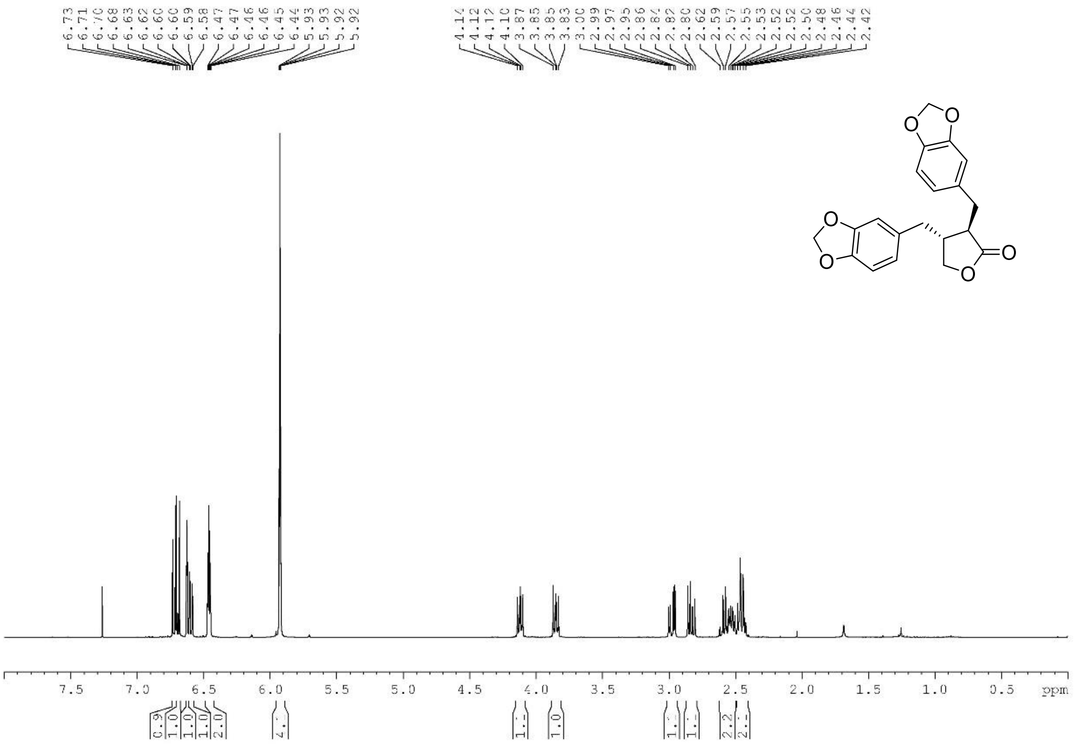

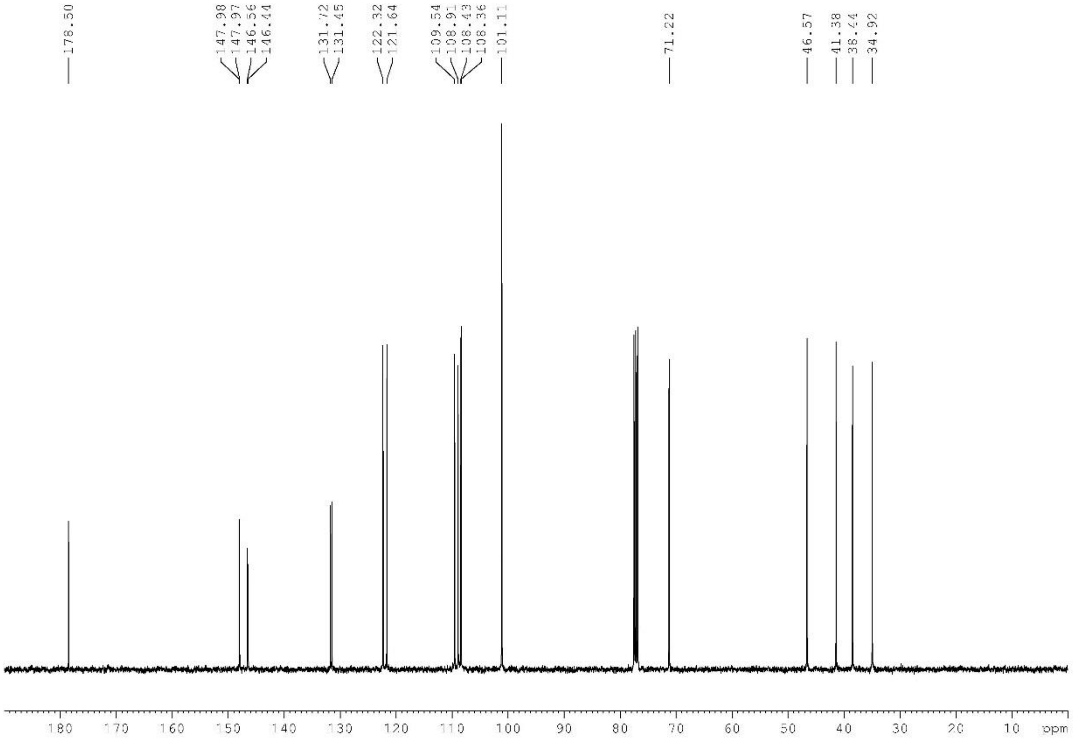

(±)-Kusunokinin **1ba**

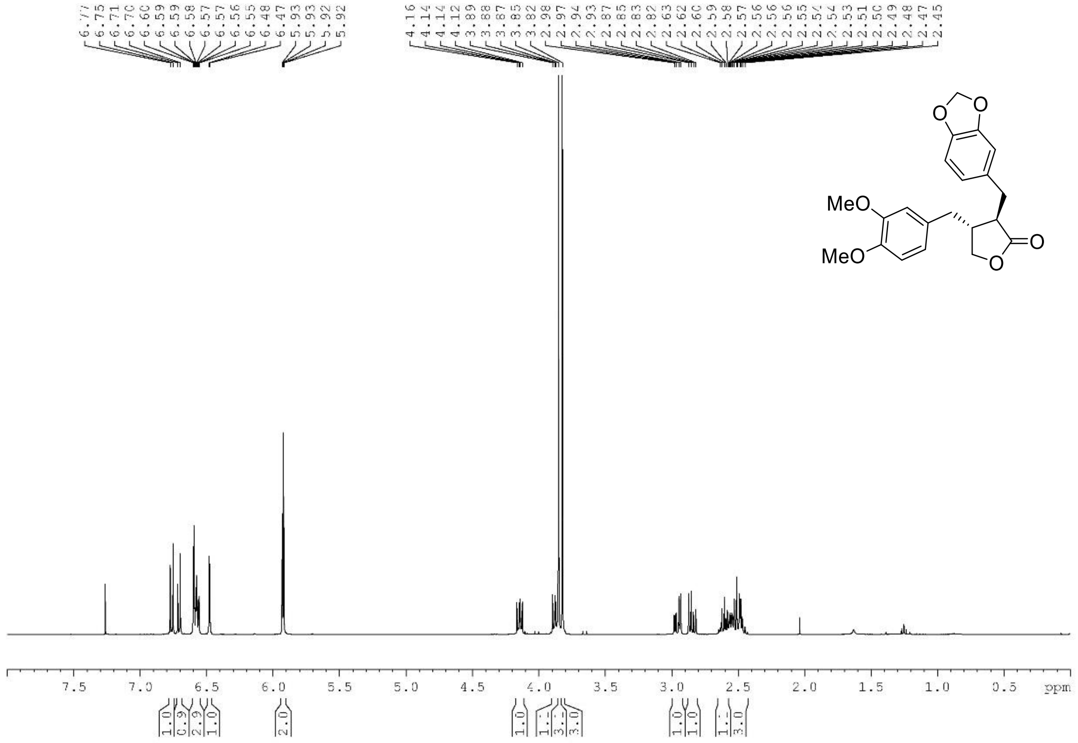

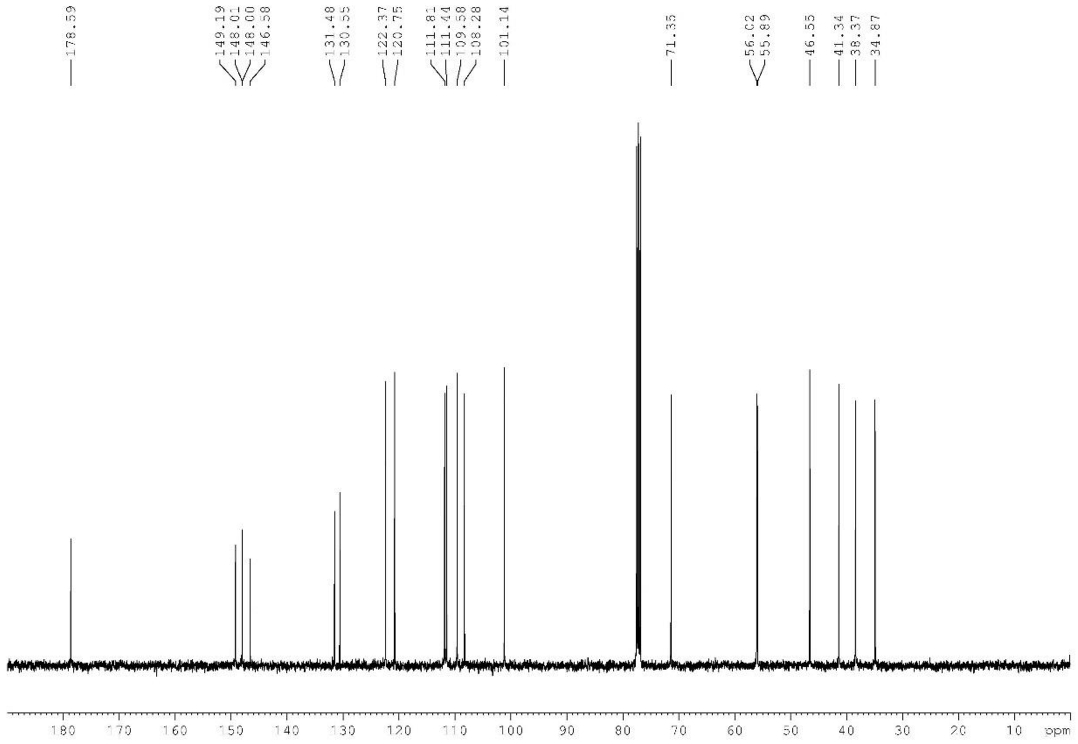

(±)-Isoyatein **1bc**

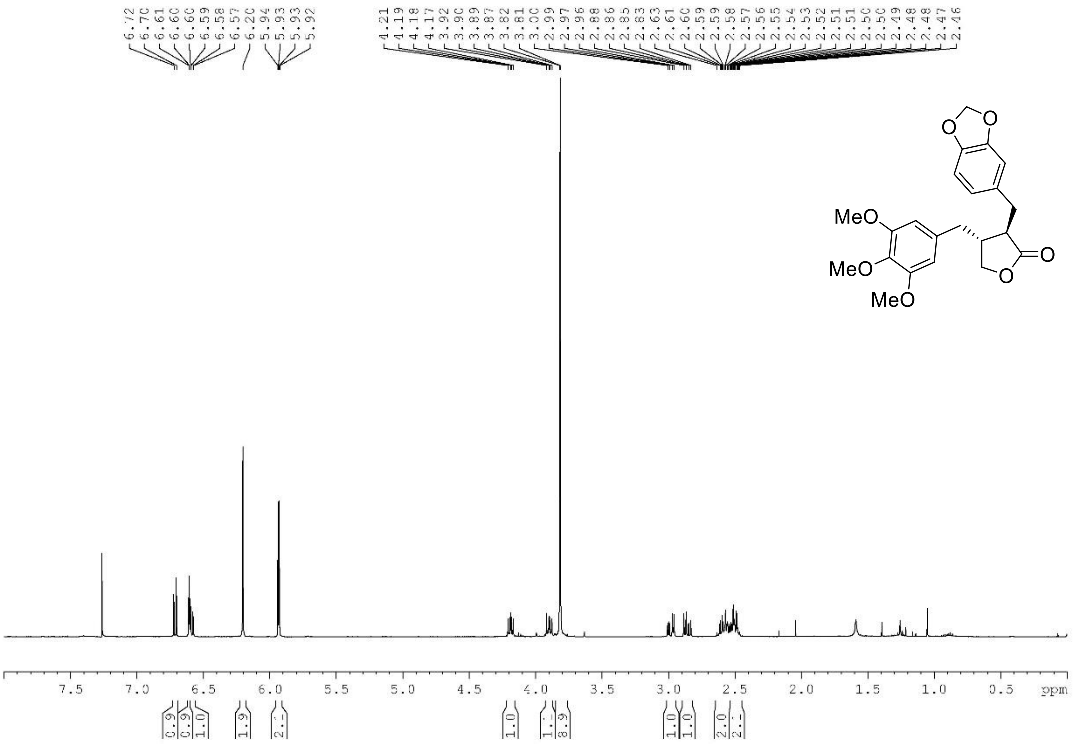

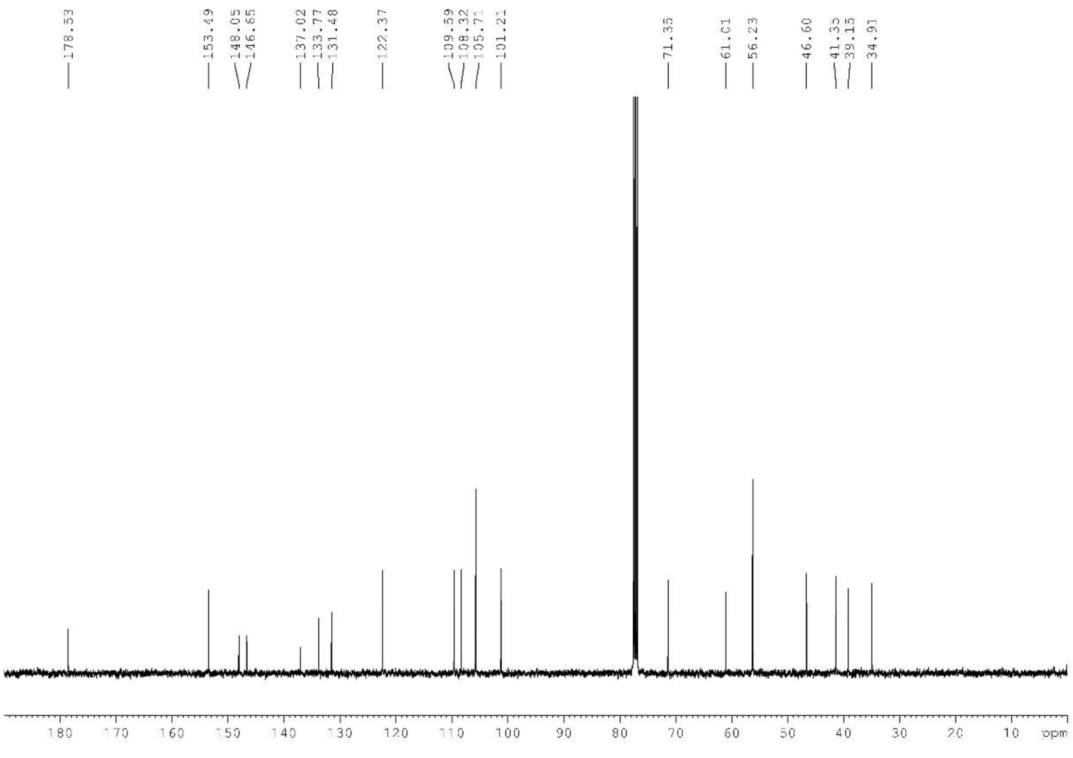

(±)-4′-*O*-Benzyl haplomyrfolin **1bd**

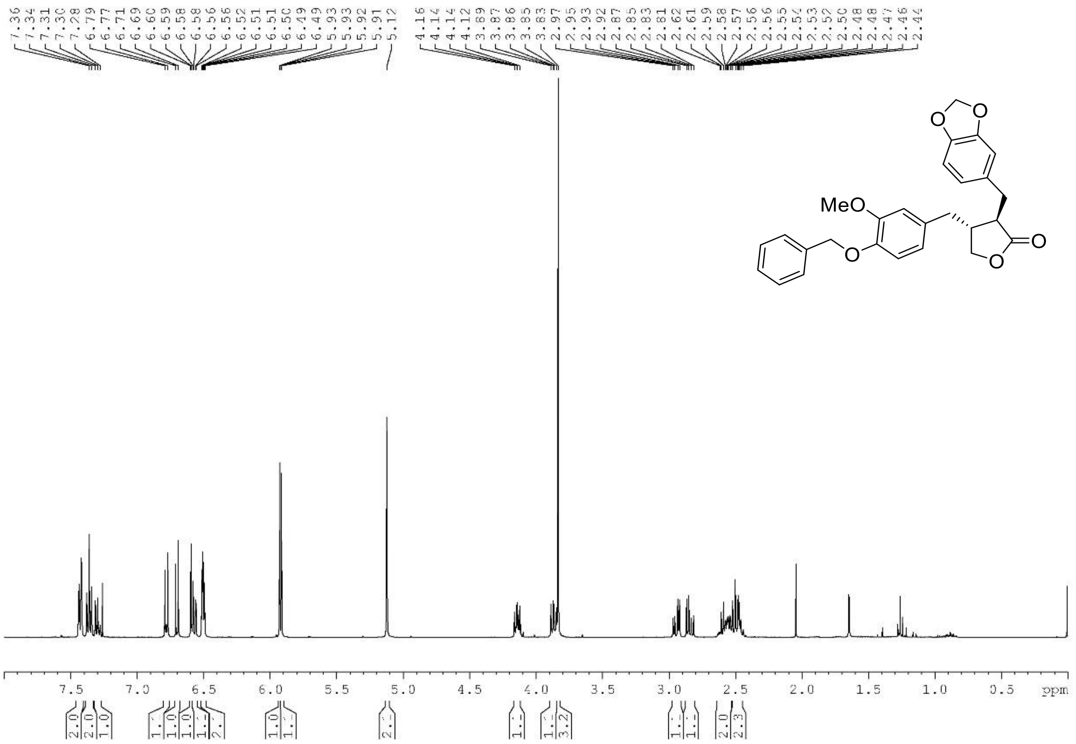

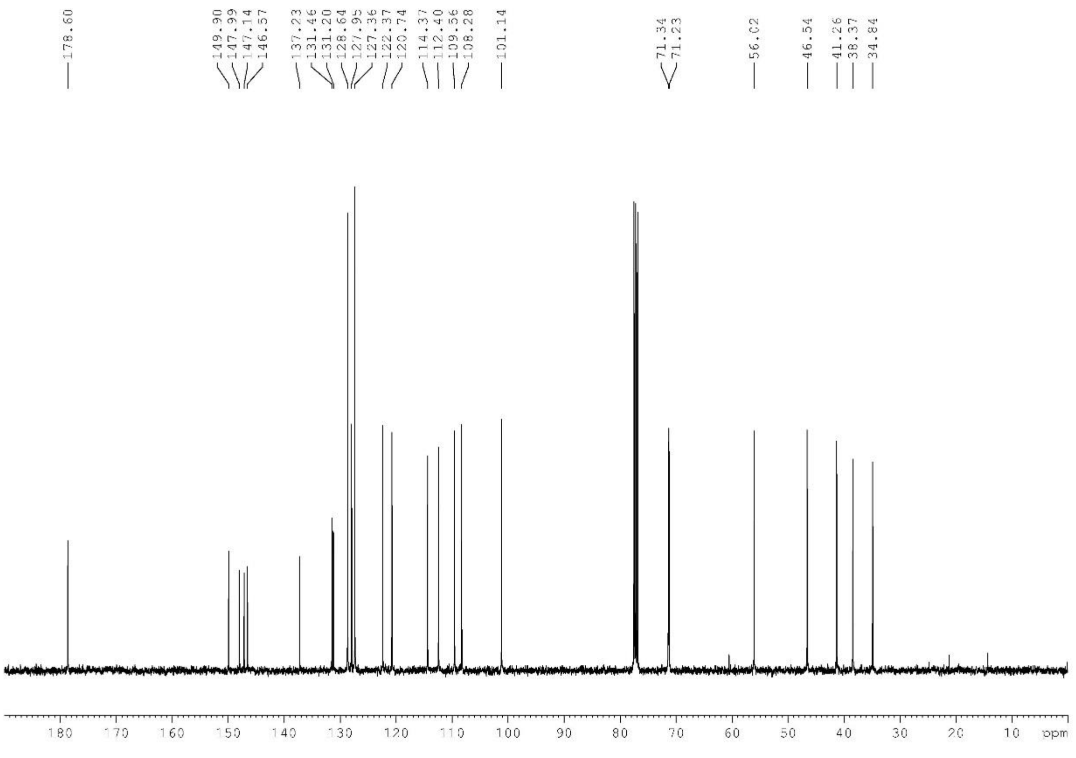

(±)-Haplomyrfolin **1be**

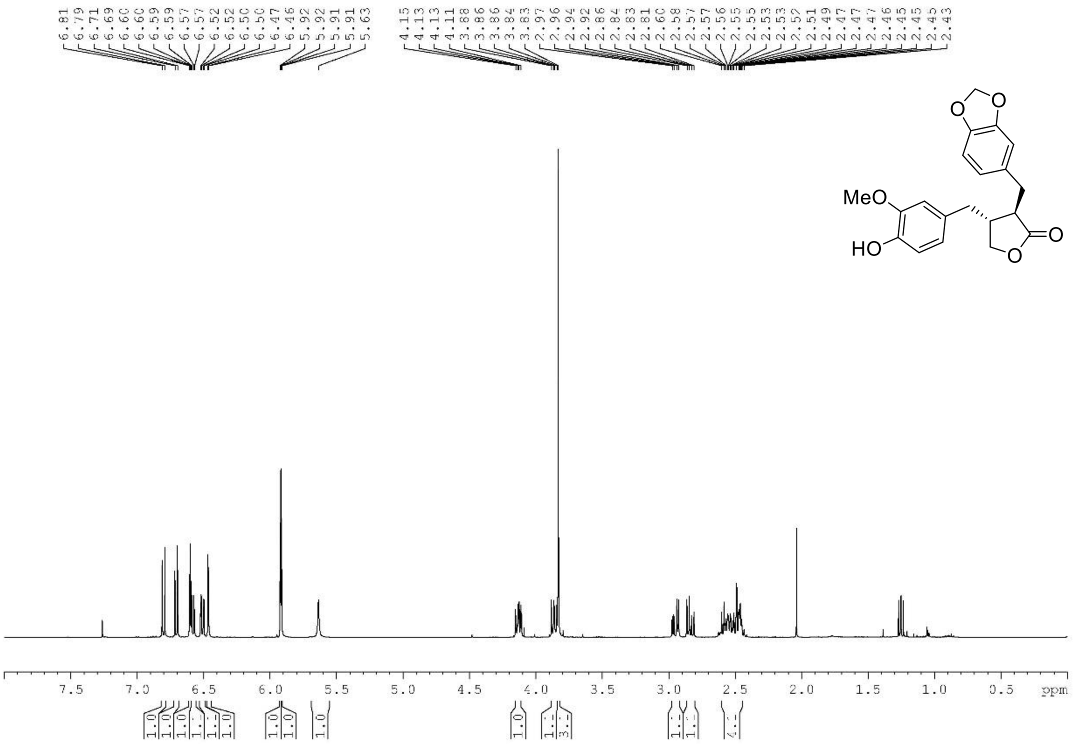

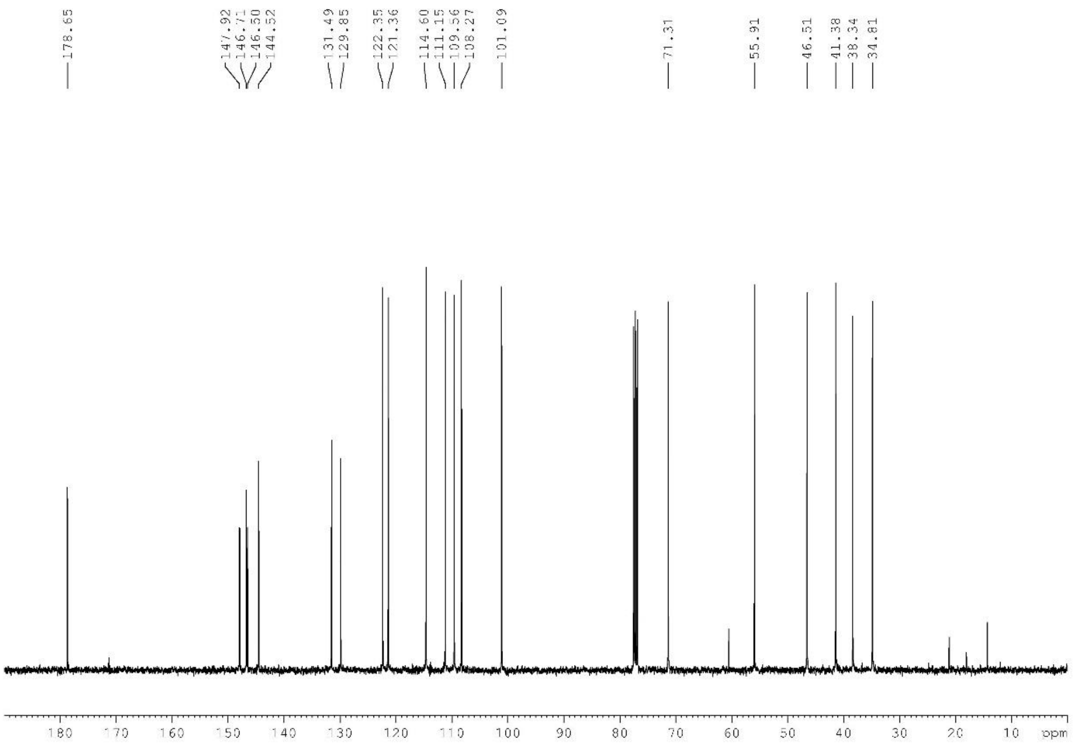

Supplement: Supplementary file 1 [file molecules-23-03057-s001.pdf]
